# The Methylbismuth Dication: Pentagonal Pyramidal Coordination and Ligand‐Induced Lewis Superacidity

**DOI:** 10.1002/anie.202516140

**Published:** 2025-11-17

**Authors:** Johannes Schwarzmann, Tamina Z. Kirsch, Benedikt Narz, Crispin Lichtenberg

**Affiliations:** ^1^ Department of Inorganic Chemistry Philipps‐Universität Marburg Hans‐Meerwein‐Straße 4 35043 Marburg Germany

**Keywords:** Bismuth, Coordination chemistry, Dicationic species, Lewis superacid, Pentagonal pyramidal coordination

## Abstract

A profound understanding of the naturally preferred coordination geometry of molecular complexes is the basis for scientists to rationalize, predict, and design their physico‐chemical properties and reactivity. Gaining access to compounds with an unusual coordination chemistry and developing a fundamental knowledge about their properties represent key challenges in the field. Tackling these questions promises an entry to unexplored chemical space and reactivity patterns that are inaccessible for compounds found in more traditional coordination geometries. Here, we present the synthesis, isolation, and characterization of the simplest organobismuth dication, the methylbismuth dication [BiMe(thf)_5_][SbF_6_]_2_ (**1**), stabilized only by five substitutionally labile THF ligands. The hexa‐coordinate compound shows a rare pentagonal pyramidal coordination geometry around the central atom, which is extremely unusual given the fact that this is an organometallic species with only simple monodentate monoanionic and/or neutral ligands without considerable steric bulk. The detailed investigation of this compound with experimental and theoretical approaches reveals the cause of the unusual coordination chemistry and uncovers multiple Lewis acidic binding sites and ligand‐induced Lewis superacidity.

## Graphical abstract

The simplistic methylbismuth dication has been captured only by the aid of monodentate tetrahydrofuran (thf) ligands, [BiMe(thf)_5_]^2+^. The hexacoordinate species shows a very unusual pentagonal pyramidal coordination geometry. Unparalleled Lewis acidic and Lewis superacidic properties have been revealed by exploiting the coordination chemical properties of the “BiMe^2+^” complex fragment and the substitutional lability of the thf ligands.

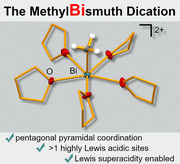

Alfred Werner's fundamental insights have taught chemists about the paramount importance of the three‐dimensional shape of molecular coordination entities: it essentially determines their physical and chemical characteristics.^[^ ^1^, ^2^, ^3^, ^4^ ^]^ Coordination numbers (CNs) ranging all the way from one at least up to 12 have been reported for metal complexes with monodentate ligands.^[^ ^5^, ^6^, ^7^, ^8^, ^9^ ^]^ Hexacoordinate species (CN = 6) strongly favor the octahedral coordination geometry,^[^ ^10^ ^]^ which typically represents the text book example when teaching first lessons in coordination chemistry. Other coordination geometries for hexacoordinate species include trigonal prismatic,^[^ ^10^, ^11^ ^]^ and rarely pentagonal pyramidal or even hexagonal planar cases.^[^ ^12^, ^13^, ^14^, ^15^, ^16^, ^17^, ^18^ ^]^ Oftentimes, these examples do not consider secondary interactions with (weakly) coordinating counter ions, which would lead to a higher coordination number, or their structure in solution cannot be identified unambiguously. Strategies to willingly generate uncommon coordination geometries exploit the use of multidentate ligands,^[^ ^19^, ^20^, ^21^, ^22^, ^23^, ^24^, ^25^, ^26^, ^27^, ^28^, ^29^, ^30^, ^31^, ^32^, ^33^, ^34^ ^]^ ligand‐induced geometric constraints,^[^ ^35^, ^36^, ^37^, ^38^, ^39^, ^40^, ^41^ ^]^ steric protection of an open coordination site,^[^ ^42^, ^43^, ^44^, ^45^, ^46^, ^47^ ^]^ or a combination thereof. The influence of a stereochemically active lone pair has also been discussed in some cases.^[^ ^48^, ^49^ ^]^ These strategies are extremely valuable for the design and tuning of molecular shapes and properties. On the contrary, it is fundamentally important to understand, which coordination geometry is naturally preferred for a central atom under given conditions. In this context, the following points promise robust and reliable insights into this first‐principle question of coordination chemistry: i) monodentate ligands should be used, ii) anionic ligands in the first coordination sphere of the central atom should be simple, prototypical representatives such as (pseudo‐)halides and alkyl groups, iii) there should be no excessive steric bulk, iv) inter‐ligand interactions should be avoided, v) there should be no significant inter‐molecular interactions. The latter point explicitly includes charged molecular entities to be investigated, if sufficiently weakly coordinating counterions are utilized.

When aiming at naturally preferred unusual coordination geometries with high coordination numbers of CN ≥ 6 for main group compounds, the investigation of charged species, the use of moderately σ‐donating ligands, and the focus on central atoms with large atomic radii have been valuable approaches.^[^ ^13^, ^15^, ^50^, ^51^, ^52^, ^53^, ^54^ ^]^ In this context, we hypothesized that a simple charged building block [M–R]^n+^, held together by covalent bonding schemes, should electronically disfavor bonding interactions in *trans*‐position of R (due to the σ*(M–R) orbital being high in energy), while electrostatically attracting a larger number of neutral ligands via dative bonding in the equatorial plane. For literature‐known motifs [Bi–R]^2+^ (R = Cl, Ph), high coordination numbers of five to nine have been reported (Figure 1a–d).^[^ ^55^, ^56^, ^57^, ^58^ ^]^ However, the criteria discussed above are not met due to the use of multidentate ligands and/or significant bonding interactions with counteranions.

**Figure 1:**
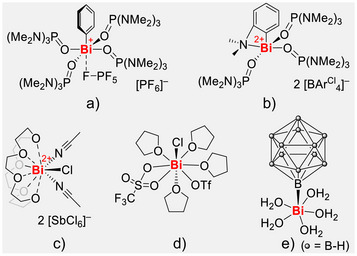
Literature examples of dicationic bismuth complexes a)–d) and related compound e), for a more detailed overview see Supporting Information.

Here, we report the synthesis, isolation, and full characterization of the methylbismuth dication, [BiMe]^2+^, stabilized by five labile thf ligands, resulting in the extremely unusual pentagonal pyramidal coordination geometry and Lewis acidic properties associated with the availability of multiple binding sites in close proximity to each other.

The target compound [BiMe(thf)_5_][SbF_6_]_2_ (**1**) was readily obtained in 62% yield from BiMeCl_2_ and AgSbF_6_ in a simple salt elimination reaction (Scheme 1a). ^1^H NMR spectroscopic analysis in CD_2_Cl_2_ shows one singlet at *δ* = 2.40 ppm as well as two multiplets at *δ* = 2.04 and 4.06 ppm with relative integrals of 3:20:20. The singlet is assigned to the bismuth‐bound methyl group and experiences a significant downfield‐shift compared to BiMe_3_ (*δ* = 1.11 ppm) and [BiMe_2_(SbF_6_)] (*δ* = 2.28 ppm), indicating the pronounced electron‐withdrawing character of the metal atom in the dicationic complex ion, despite the presence of five neutral ligands.^[^ ^59^ ^]^ This is further supported by a downfield‐shift of the multiplets (assigned to the bismuth‐bound thf ligands) by 0.22–0.37 ppm relative to free THF.^[^ ^60^ ^]^ The ^13^C NMR spectrum corroborates this scenario, as the resonance for the methyl group is detected at *δ* = 88.7 ppm (*δ* = 64.36 for [BiMe_2_(SbF_6_)])^[^ ^59^ ^]^ and those for the THF ligands appear at *δ* = 25.95 and 71.37 ppm (*δ* = 25.98 and 68.16 ppm for free THF).^[^ ^60^ ^]^ The ^19^F NMR spectrum shows a complex signal of a sextet (due to ^1^ *J*_SbF_ coupling with ^121^Sb (natural abundancy: 57%, *I* = 5/2)) overlapping with an octet (due to ^1^ *J*_SbF_ coupling with ^123^Sb (natural abundancy: 43%, *I* = 7/2)), which is typical for [SbF_6_]^–^ anions that do not show significant directional bonding interactions with counterions.^[^ ^61^ ^]^

**Scheme 1:**
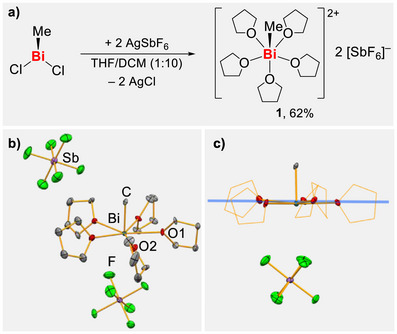
a) Synthesis of [BiMe(thf)_5_][SbF_6_]_2_ (**1**). b) Molecular structure of **1**: Displacement ellipsoids are drawn at 50% probability level. Hydrogen atoms are omitted for clarity. Selected bond lengths [Å] and angles [°]: Bi─C 2.192(6), Bi─O1‐5 2.443(4)‐2.485(5), Bi⋅⋅⋅F 3.593(6), C─Bi─O1 84.6(2), O1─Bi─O2 71.61(17). c) Molecular structure of **1** viewed along the plane (marked in blue) set up by the coordinating oxygen atoms of the thf ligands, the second SbF_6_^–^ unit was omitted for clarity.

NMR spectroscopy and elemental analysis indicated the presence of five thf ligands–a unique scenario for the rare examples of simple organobismuth dications, which commonly accommodate four neutral ligands.^[^ ^56^, ^62^ ^]^ This motivated more detailed investigations by single‐crystal X‐ray analysis. **1** crystallizes in the monoclinic space group *P2_1_/c* with *Z* = 4 (Scheme 1b). The diffraction experiment confirmed the presence of five thf ligands in the coordination sphere of the bismuth atom in addition to the methyl group. Remarkably, all the neutral ligands are accommodated in the equatorial plane (angle sum O─Bi─O, 359.8°, see Scheme 1c). The first coordination sphere is completed by the methyl group in the apical position. Importantly, no directional bonding interactions between the bismuth atom and the [SbF_6_]^–^ counteranions can be detected based on distance criteria, as the shortest interatomic Bi···F distance (3.593(6) to 3.799(5) Å) exceed the sum of the van der Waals radii (3.54 Å),^[^ ^63^ ^]^ when applying three‐sigma‐limits. Thus, neither in DCM solution nor in the solid state, are there indications of significant directional bonding interactions between the bismuth center and the [SbF_6_]^–^ counteranions. This leads to an extremely unusual pentagonal pyramidal coordination geometry around the bismuth center without additional weak bonding interactions. Exploiting only simple monodentate ligands, this is – to the best of our knowledge – an unprecedented case in the organometallic chemistry of main group compounds.^[^ ^14^, ^15^, ^16^, ^23^, ^64^, ^65^, ^66^, ^67^, ^68^ ^]^ An example of an intriguing bismuth compound with a pentagonal pyramidal coordination geometry has been introduced to the literature (Figure 1,e), but the use of a bulky, rather uncommon inorganic *closo*‐borate cluster [B_12_H_11_]^3−^ as a bulky *tri*anionic ligand was necessary to get a first glimpse at this compound, which could not be isolated in pure form and for which a yield has not been reported.^[^ ^69^ ^]^ In other cases multidentate ligands have been used and oligonuclear structures have been obtained.^[^ ^70^, ^71^, ^72^, ^73^, ^74^ ^]^

The bismuth–carbon bond in **1** measures 2.192(6) Å and it is slightly shorter than the analogous bonds in the monocationic [BiMe_2_(SbF_6_)] and adducts thereof (2.215(5) to 2.223(5) Å).^[^ ^59^, ^61^ ^]^ In comparison with aryl bismuth dications, the Bi─C bond in **1** falls within the broad range of values that has previously been reported (2.158(9) to 2.216(8) Å),^[^ ^56^, ^62^, ^70^, ^75^, ^76^ ^]^ emphasizing the impact of coordination number, chelation, and donor strength on the bonding parameters of these compounds. Despite the large number of five thf ligands populating the two vacant p_x_(Bi) and p_y_(Bi) atomic orbitals of the bismuth center in the basal plane, the Bi─O bond lengths (2.44–2.49 Å) remain in the broad range of those reported for related monocationic bismuth compounds with thf ligands (2.40–2.76 Å).^[^ ^77^, ^78^, ^79^, ^80^ ^]^

In order to gain deeper insights into the unusual coordination chemistry of compound **1**, DFT calculations on the B3LYP‐D3/def2‐TZVP level of theory were performed with a polarizable continuum solvent model for the reaction medium THF. Aiming to understand the naturally preferred coordination number and coordination geometry of the methylbismuth dication in solution, compounds [BiMe(thf)_n_]^2+^ (*n* = 0–6) were analyzed. The subsequent addition of thf ligands to the Lewis acidic bismuth center is exergonic until five thf ligands are coordinated to the central atom (Scheme 2a). This leads to an overall coordination number of six in a pentagonal pyramidal coordination geometry (PPY‐6). Remarkably, the isomer with a distorted octahedral coordination geometry (OC‐6) represents a local minimum on the potential energy hypersurface, but is 9.2 kcal·mol^−1^ higher in energy (Supporting Information). Adding a sixth thf ligand to the [BiMe]^2+^ complex fragment leads to a compound with a pentagonal bipyramidal coordination geometry (PBPY‐7). Importantly, however, the formation of this heptacoordinate species is energetically disfavored by 3.1 kcal·mol^−1^. This is in contrast to the related compounds [BiCl_2_(py)_5_]^+^ and [BiCl(dmso)_6_]^2+^, for which hepta‐coordination is thermodynamically favored. Thus, the suggestion of a stereochemically active lone pair as the key factor for favoring a pentagonal pyramidal coordination geometry of a bismuth(III) compound appears questionable. In order to understand the nature of the bismuth‐centered lone pair in [BiMe(thf)_5_]^2+^, an extensive molecular orbital analysis was performed. Molecular orbitals (MOs) with a significant bismuth‐lone pair character are remarkably low in energy (e.g.: HOMO‐43, HOMO‐53, HOMO‐63). The contribution of bismuth‐centered atomic orbitals to these MOs range from 13%–35% and are exclusively of s‐type character according to an orbital composition analysis with Mulliken partition (Supporting Information). In agreement with this, intrinsic bond orbital (IBO) analyses identify the bismuth‐centered lone pair at an energy level that lies 7.22 eV below the HOMO, i.e., it has essentially core orbital character. Furthermore, its shape is close to spherical (Scheme 2b, left). So, what is the reason for the reluctance to populate the vacant binding site *trans* to the methyl group in [BiMe(thf)_5_]^2+^ in the PPY‐6 geometry? In a bonding scheme relying on significant orbital interactions, the complex [BiMe(thf)_5_]^2+^ would need to provide a suitably oriented vacant MO in order to effectively bind an additional donor ligand. In [BiMe(thf)_5_]^2+^, this would be the σ*(Bi─C) orbital. In agreement with the only weakly polar nature of the Bi─C bond and the relatively small difference in the (group) electronegativities of Bi and CH_3_, the σ*(Bi─C) orbital is high in energy. According to IBO analyses, it corresponds to the LUMO + 2 and is energetically located 5.4 eV above the LUMO (Scheme 2b, right). The electrostatic potential map of [BiMe]^2+^ further supports this analysis, as the highest values are found in the plane perpendicular to the Bi─C bond, not in the position *trans* to the methyl group (Supporting Information).

**Scheme 2:**
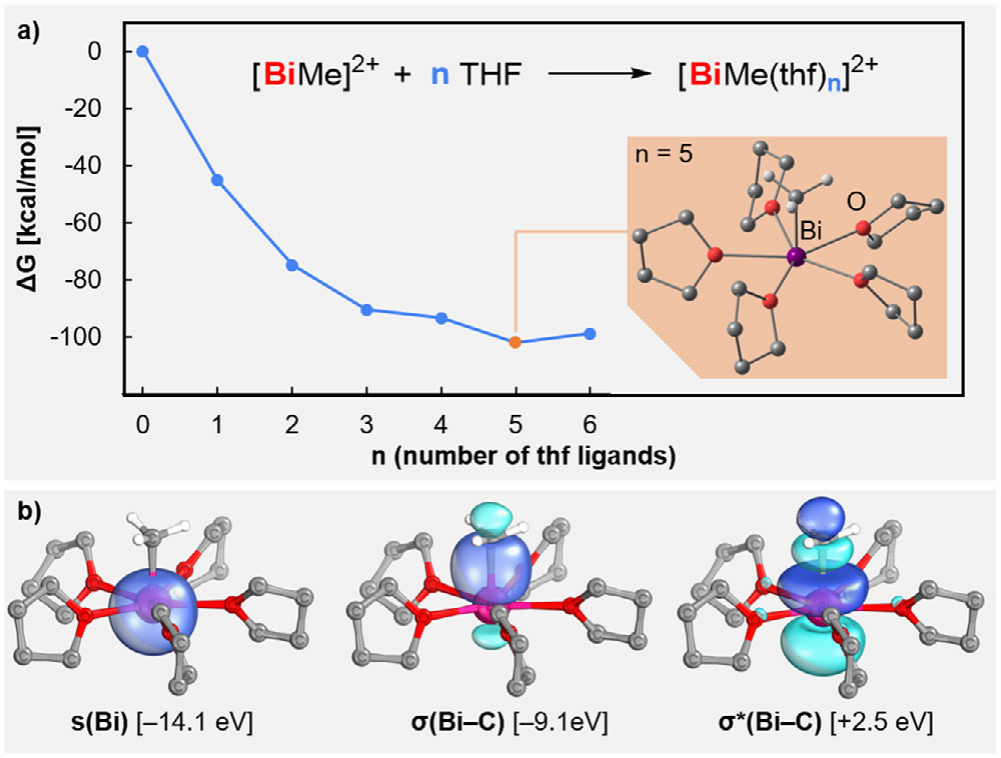
a) Gibbs energy of adduct formation between [BiMe]^2+^ and n equivalents of THF, as determined by DFT calculations including geometry‐optimization. b) Selected orbitals (threshold = 80) and orbital energies as determined by IBO analysis of [BiMe(thf)_5_]^2+^ (for comparison: energies of HOMO / LUMO are at −6.9 eV/−2.9 eV in the IBO analyses).

These investigations predict a poor Lewis acidity for compound **1** if the thf ligands are substitutionally inert. However, ^1^H NMR spectroscopic analysis of **1** in THF‐*d_8_* demonstrate rapid exchange of thf ligands and THF solvent molecules, indicating the substitutionally labile nature of the neutral ligands. This is further confirmed by the selective formation of [BiMe(py)_5_][SbF_6_]_2_ (**2**) from solutions of **1** in pyridine (for full characterization of **2** see Supporting Information). Taking into account the facile substitution of the thf ligands in **1**, its Lewis acidity was quantitatively investigated with the (modified) Gutmann–Beckett method.^[^ ^78^, ^81^, ^82^, ^83^ ^]^ This method evaluates the effective Lewis acidity using OPEt_3_, SPMe_3_, and SePMe_3_ as Lewis basic reporter molecules of varying hardness/softness according to the Pearson concept. ^31^P NMR chemical shifts of solutions containing the Lewis acid and varying amounts of the donor EPR_3_ (E = O─Se, R = Me, Et) are translated into an acceptor number (AN), where large acceptor numbers are associated with a high Lewis acidity. Several factors can have a tremendous influence on the ANs. For instance, the presence of only one equivalent of a competing Lewis base such as THF can essentially block the coordination site of archetypical examples of Lewis acids such as B(C_6_F_5_)_3_ and AlCl_3_.^[^ ^78^ ^]^ Competition of ligands (such as solvent molecules) with the probe EPR_3_ for a binding site at the Lewis acidic center can lead to high acceptor numbers being observed only with an excess of the Lewis acid (which is an unwanted or even unrealistic scenario for most potential applications).^[^ ^84^, ^85^, ^86^ ^]^ Furthermore, the simultaneous activation of two substrate molecules at one central atom can be a target scenario, as it has been reported to open up unparalleled reaction pathways.^[^ ^87^, ^88^, ^89^, ^90^, ^91^ ^]^ However, the accessibility of two or more Lewis acidic binding sites in a *cis* arrangement at one Lewis acidic center is oftentimes not ensured.^[^ ^61^ ^]^ More importantly, binding two substrate molecules to one Lewis acidic center tends to significantly decrease the substrate activation, as reflected by ANs experiencing a significant decrease when changing the molar ratio of Lewis acid / EPR_3_ from 5:1 over 1:1 to 1:2.^[^ ^61^, ^84^, ^85^, ^86^ ^]^ This poses the question of how to efficiently activate two or more Lewis basic substrate molecules in close proximity to each other at one Lewis acidic center. The Gutmann–Beckett analysis of compound **1** revealed ANs of 83–94, when one equivalent of the phosphane chalcogenide EPR_3_ was used, which is at the upper range of ANs found for cationic bismuth compounds (Scheme 3a).^[^ ^50^, ^61^, ^77^, ^78^, ^84^, ^85^, ^92^, ^93^, ^94^, ^95^ ^]^ It must be emphasized that these values are obtained for precursor **1**, which is electronically saturated through thf‐coordination. Remarkably, these high ANs are essentially unchanged when 1.5 equivalents of the donor are applied and remain at a high level, when two equivalents of the donor are used (Scheme 3a). Investigations into the coordination chemistry of adducts between the [BiMe]^2+^ complex fragment and the Gutmann–Beckett donors EPR_3_ indicate dynamic bonding scenarios with ligand exchange equilibria (Supporting Information). Products containing two to four equivalents of these donors could be unambiguously identified as isolable species **3**, **4‐MeCN**, and **5**, which could subsequently be rationally synthesized and fully characterized (Scheme 3 and Supporting Information). The analytical data indicate significant alterations of the spectroscopic and structural parameters of the coordinated molecules EPR_3_ compared to their free form (Supporting Information). They are invariably located in close proximity to each other in the basal plane of square pyramidal coordination polyhedra. Notably, even a structural snap‐shot of the three‐coordinate species [BiMe(SPMe_3_)_2_(SbF_6_)_2_] (**4**) could be obtained, in which the twofold positive charge of the [BiMe]^2+^ complex fragment is mainly compensated by two SPMe_3_ ligands.

**Scheme 3:**
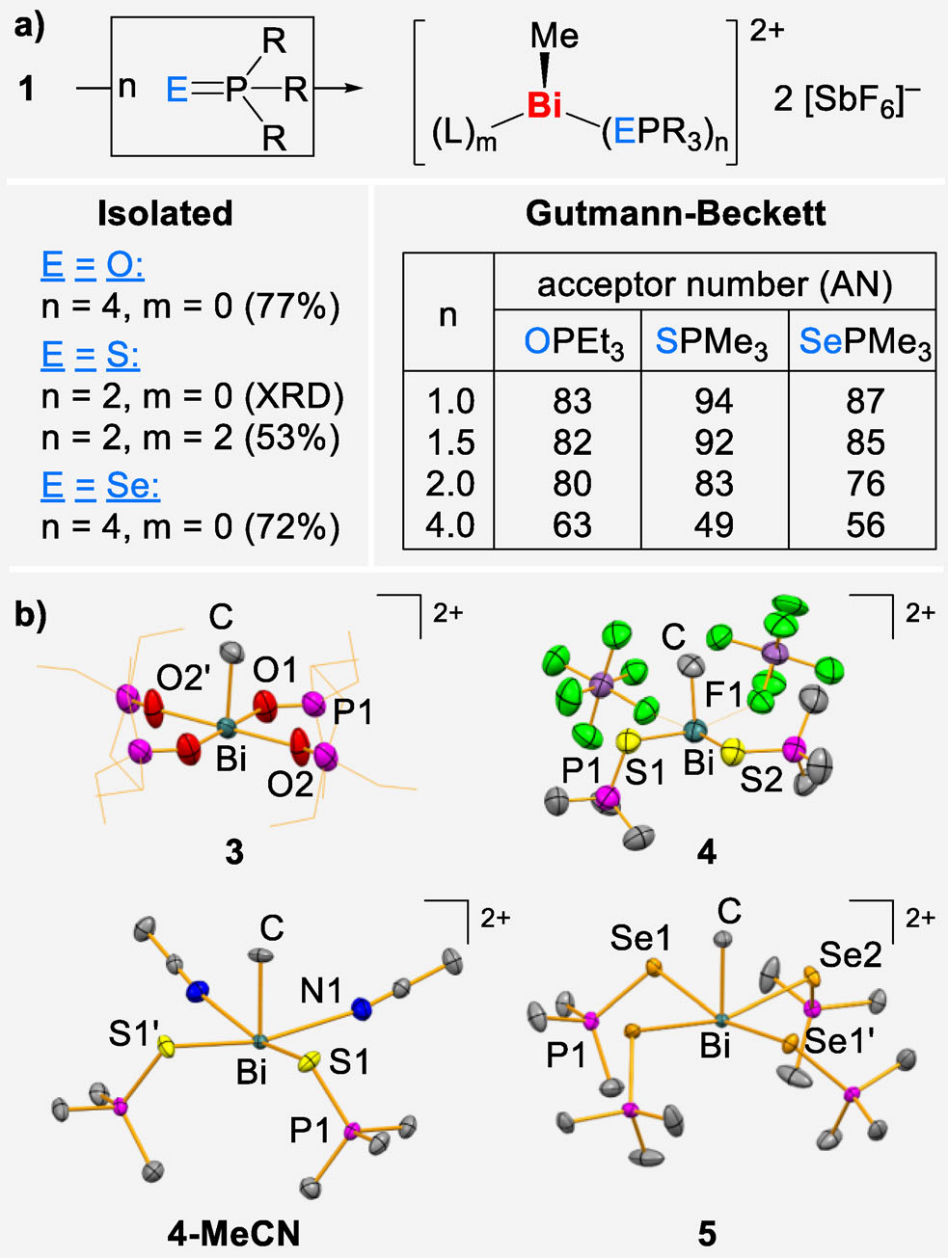
a) Reaction of **1** with the donors EPR_3_ of the (modified) Gutmann–Beckett method (E/R = O/Et, S/Me, Se/Me) and b) molecular structures of isolated products **3**, **4** (as a proof of connectivity), **4‐MeCN**, and **5**: Displacement ellipsoids are drawn at 50% probability level. Hydrogen atoms, lattice‐bound solvent molecules (for **5**), and counterions (for **3**, **4‐MeCN**, and **5**) are omitted, Ethyl groups (in **3**) shown as wireframe for clarity. Selected bond lengths [Å] and angles [°]: **3**: Bi─C 2.280(9), Bi─O1 2.314(4), Bi─O2 2.329(3), C─Bi─O1 93.0(3), O1─Bi─O2 89.82(12); **4‐MeCN**: Bi1–C1 2.224(8), Bi1─S1 2.6597(15), Bi1─N1 2.711(5), Bi1⋅⋅⋅F14 3.396(9), C1─Bi1─S1 83.16(17), C1─Bi1─N1 79.29(18), S1─Bi1─S1’ 86.51(7) S1─Bi1─N1 83.51(12); **5**: Bi─C 2.247(5), Bi─Se1 2.9476(4), Bi─Se2 2.9344(4), Bi⋅⋅⋅F 3.519(9), C─Bi─Se1 82.100(9), C─Bi─Se2 81.159(9), Se1─Bi─Se2 87.105(12), Se2─Bi─Se1’ 90.472(12).

The preference of compounds **1** and **2** to accommodate five thf or pyridine ligands in the basal plane of a pentagonal pyramidal coordination polyhedron spurred us to address the possibility of ligand‐induced enhancement of Lewis acidity,^[^ ^96^ ^]^ driven by geometrical changes in the complex. Specifically, compound **1** was reacted with two equivalents phenanthroline and 2,2′‐bipyridine. This would prohibit a simple penta‐coordination in the basal plane due to i) the bidentate character of the *N*,*N* chelating ligand and ii) the coplanar nature of the aromatic rings which prohibits a paddle‐wheel arrangement of the donor ligands. This would leave one open coordination site in the basal plane with increased Lewis acidity. Indeed, the reaction of **1** with two equivalents of the aromatic *N*,*N*‐chelating ligands yielded products with a fluoride ligand in a bridging coordination mode between two bismuth centers [(L_2_)_2_MeBi‐(μ_2_‐F)‐BiMe(L_2_)_2_][SbF_6_]_3_ with L_2_ = phenanthroline (**6**) and L_2_ = 2,2′‐bipyridine (**7**). Compounds **6** and **7** were isolated in 86%–89% yield, demonstrating the ability of **1** to abstract a fluoride ion from [SbF_6_]^–^ in the presence of *N*,*N*‐chelating ligands, lending Lewis superacidic properties to the assumed intermediates [BiMe(L_2_)_2_]^2+^. This was supported by theoretically determined fluoride ion affinities (FIAs), which show that the abstraction of F^–^ from [SbF_6_]^–^ by [BiMe(phen)_2_]^2+^ is possible, solvent‐dependent, and more effective when the formation of the experimentally observed dinuclear (rather than a mononuclear) motif is considered (Scheme 4c and Supporting Information). For instance, an FIA of 418 kJ·mol^−1^ was theoretically determined for two equivalents of [BiMe(phen)_2_]^2+^ with a diethyl ether solvent model (while an FIA of 390 kJ·mol^−1^ was calculated for SbF_5_ at the same level of theory).

**Scheme 4:**
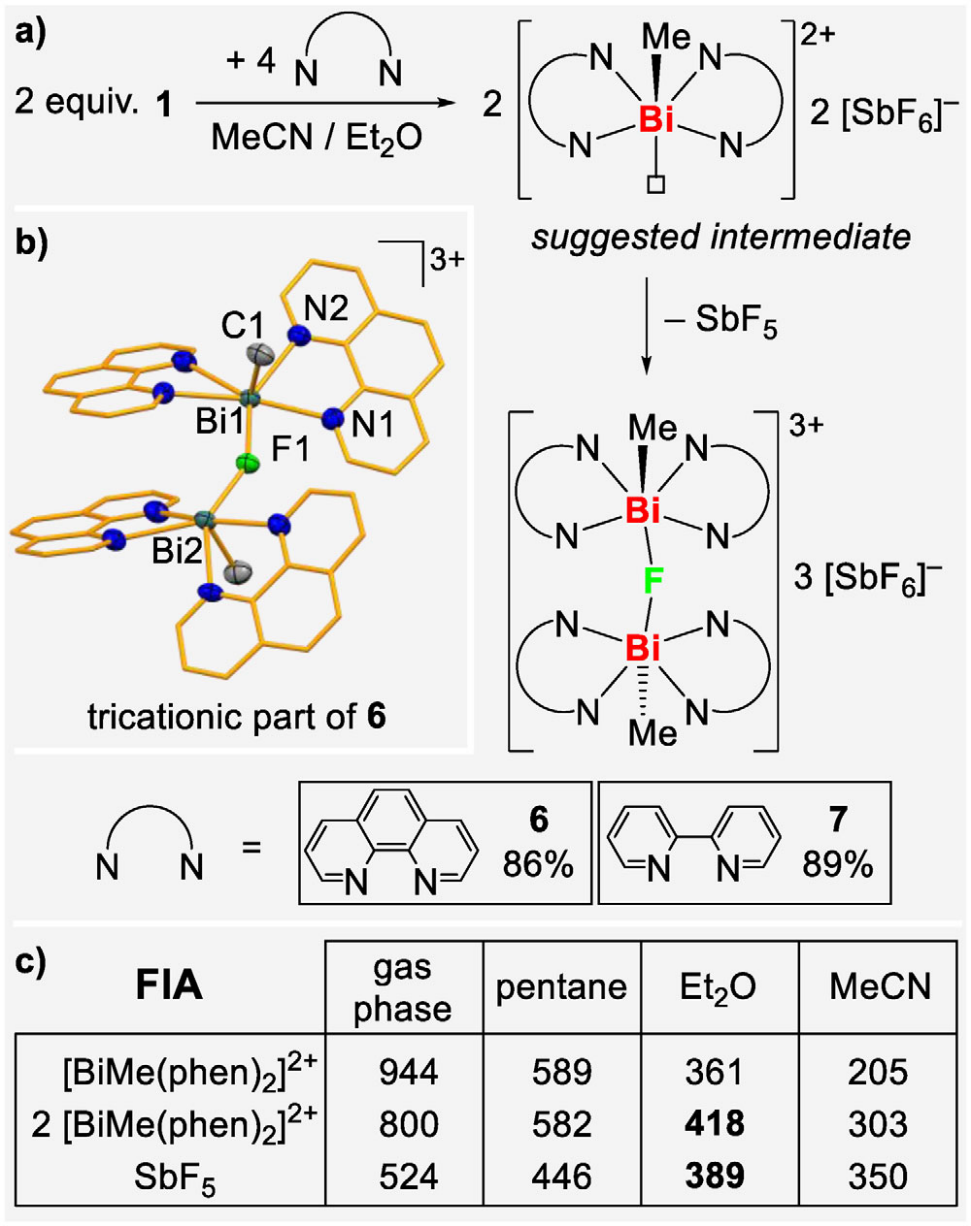
a) Reactions of **1** with phenanthroline and 2,2′‐bipyridine, to give isolated compounds **6** and **7**. b) Molecular structure of **6**; displacement ellipsoids are drawn at 50% probability level. Hydrogen atoms and three [SbF_6_]^–^ anions are omitted and carbon atoms of phenanthroline ligands are shown in the capped stick model for clarity. Selected bond lengths [Å] and angles [°]: **6**: Bi1─C1 2.221(5), Bi1─F1 2.323(3), Bi1─N1 2.456(4), Bi1─N3 2.743(4), Bi1⋅⋅⋅F8 3.331(3), Bi2─C2 2.228(5), Bi2─F1 2.313(3), Bi2─N6 2.684(4), Bi2─N8 2.481(4), Bi2⋅⋅⋅F4 3.467(5), Bi1─F1─Bi2 149.59(14), C1─Bi1─N1 89.57(18), C1─Bi1─N3 112.27(17), C1─Bi1─F1 87.52(15), N1─Bi1─N2 67.43(14), N2─Bi1─N3 82.52(13), C2─Bi2─N6 107.15(17), C2─Bi2─N7 77.78(17), C2─Bi2─F1 87.22(16). c) Fluoride ion affinity (FIA) of selected compounds in kJ·mol^−1^ calculated at the B3LYP/def2‐tzvp level of theory using SiMe_3_F as an anchor point (for further details and discussion, see Supporting Information).

In conclusion we present the synthesis and full characterization of the first alkyl‐substituted bismuth dication, stabilized by five monodentate thf ligands, [BiMe(thf)_5_][SbF_6_]_2_. This compound shows a very unusual pentagonal pyramidal coordination geometry. Remarkably, this is realized i) with simple, archetypical monoanionic or neutral ligands, ii) without extensive steric load, geometry constraints, or chelation, iii) without a decisive role of a “stereochemically active lone pair”, but rather due to iv) the vacant σ*(Bi─C) orbital being high in energy. The thf ligands in [BiMe(thf)_5_]^2+^ are substitutionally labile, leading to a pronounced Lewis acidity of this complex cation. Importantly, the Lewis acidity of this species remains high not only toward one, but even toward two equivalents of a substrate, opening up horizons for the effective activation of two substrate molecules in the coordination sphere of one metal atom. The introduction of chelation control and geometric constraints through the neutral bidentate ligands L in [BiMe(L)_2_]^2+^ confers Lewis superacidic properties to the methylbismuth dication, as verified experimentally and theoretically. It is anticipated that these findings will contribute to the conceptualization and design of heavy main group compounds in the context of synthetic building blocks with unusual symmetry elements as well as group transfer and catalysis in non‐conventional coordination geometries.

## Supporting Information

The authors have cited additional references within the Supporting Information.^[^ ^97^, ^98^, ^99^, ^100^, ^101^, ^102^, ^103^, ^104^, ^105^, ^106^, ^107^, ^108^, ^109^, ^110^, ^111^, ^112^, ^113^, ^114^, ^115^, ^116^, ^117^, ^118^, ^119^, ^120^, ^121^, ^122^, ^123^, ^124^, ^125^, ^126^, ^127^, ^128^, ^129^, ^130^, ^131^, ^132^ ^]^

## Conflict of Interests

The authors declare no conflict of interest.

## Supporting information



Supporting Informaton

Supporting Informaton

## Data Availability

The data that support the findings of this study are available in the Supporting Information of this article.
