# Supplementary material for: The Methylbismuth Dication: Pentagonal Pyramidal Coordination and Ligand‐Induced Lewis Superacidity
Source: Angew Chem Int Ed Engl. 2025 Nov 17;65(1):e16140. doi: 10.1002/anie.202516140 (PMC12759221; doi:10.1002/anie.202516140)
Supplement: Supplementary file 1 — Supporting Informaton [file ANIE-65-e16140-s001.pdf]

Table of Contents

1. **Experimental** S2
   1.1. **General considerations** S2
   1.2. **Preparation of [BiMe(thf)$_5$][SbF$_6$]$_2$ (1)** S3
   1.3. **Preparation of [BiMe(py)$_5$][SbF$_6$]$_2$ (2)** S3
   1.4. **Preparation of [BiMe(OPEt$_3$)$_4$][SbF$_6$]$_2$ (3)** S4
   1.5. **Preparation of [BiMe(SPMe$_3$)$_2$(MeCN)$_2$][SbF$_6$]$_2$ (4-MeCN)** S4
   1.6. **Preparation of [BiMe(SePMe$_3$)$_4$][SbF$_6$]$_2$ · MeCN (5)** S5
   1.7. **Preparation of [{BiMe(phen)$_2$}$_2$(μ-F)][SbF$_6$]$_3$ (6)** S6
   1.8. **Preparation of [{BiMe(bipy)$_2$}$_2$(μ-F)][SbF$_6$]$_3$ (7)** S7
2. **Literature overview of pentagonal pyramidal coordinated bismuth as well as important structures of bismuth dications** S8
3. **Discussion of the synthesis and characterization of [BiMe(py)$_5$][SbF$_6$]$_2$** S10
4. **Determination of acceptor numbers by the (modified) Gutmann-Beckett method** S11
5. **Synthesis and characterization of BiMe^{2+} adducts with EPR$_3$ (E=O, S, Se; R=Me, Et)** S12
6. **Computational Details** S14
7. **Fluoride ion affinities (FIAs)** S15
8. **NMR Spectra** S18
9. **High-resolution mass spectrometry (1:1 mixtures of 1 and GB-donors)** S28
10. **Single-crystal X-ray diffraction** S35
11. **Cartesian coordinates (Å) of compounds obtained from DFT geometry optimization** S39

1. Experimental

1.1. General considerations

All Experiments were, if not stated otherwise, conducted under an atmosphere of dry argon using Schlenk- and glovebox techniques. Solvents were degassed and purified according to standard laboratory procedures. NMR spectra were recorded on Bruker Avance spectrometers operating at 300, 400 or 500 MHz with respect to ^{1}H. ^{1}H- and ^{13}C-NMR chemical shifts are reported relative to $SiMe_4$ using the residual signal of the deuterated solvent as a secondary standard.[60] ^{19}F- and ^{31}P-NMR chemical shifts are reported relative to $CFCl_3$ and aqueous H_3PO_4 (85%), respectively, as external standards. IR spectra were recorded on a Bruker Alpha II with an ATR probe head. Mass spectrometry was conducted on a Thermo Fischer Scientific Orbitrap Q Exactive Plus using ESI as an ionization method. The samples were infused into the mass spectrometer under inert atmosphere through a syringe pump. Elemental analyses (C, H, N, S) were performed on a vario MICRO cube. Single-crystals suitable for X-ray diffraction were coated with polyisobutylene or perfluorinated polyether oil in a glovebox, transferred to a nylon loop and then transferred to the goniometer of a Bruker D8 Quest diffractometer equipped with a molybdenum (λ = 0.71073 Å) X-Ray tube or a STOE StadiVari diffractometer equipped with a copper (λ = 1.54186 Å) X-Ray tube. Using Olex2[97], the structure was solved with the XT[98] structure solution program using intrinsic phasing and refined with the XL refinement package using least squares minimization.[99] All non-hydrogen atoms were refined anisotropically. Hydrogen atoms were included in structure factors calculations. All hydrogen atoms were assigned to idealized geometric positions. Deposition numbers 2474536 (**1**), 2474537 (**2**), 2474538 (**3**), 2474539 (**4**), 2474540 (**4-MeCN**), 2474541 (**5**), 2474542 (**6**), and 2474543 (**7**) contain the supplementary crystallographic data for this paper. These data are provided free of charge by the joint Cambridge Crystallographic Data Centre and Fachinformationszentrum Karlsruhe Access Structures service www.ccdc.cam.ac.uk/structures.

1.2. Preparation of $[BiMe(thf)_5][SbF_6]_2$ (1)

$[BiMeCl_2]$ (100 mg, 0.34 mmol, 1 eq.) was dissolved in a mixture of THF and DCM (1 mL each). $AgSbF_6$ (232 mg, 0.68 mmol, 2 eq.) was dissolved in DCM (9 mL). Both solutions were combined and stirred for 18 h under exclusion of light. The resulting suspension was filtered and the colorless precipitate was extracted with DCM (3 × 5 mL). All solutions were combined and the product was crystallized by addition of *n*-pentane (30 mL) and storing at –30 °C for 12 h to complete precipitation. Filtration and drying *in vacuo* yields $[BiMe(thf)_5][SbF_6]_2$ as a colorless solid. (218 mg, 0.21 mmol, 62%)

^{1}H-NMR (500 MHz, CD_2Cl_2): δ = 2.03-2.05 (br, m, 20 H, thf), 2.40 (s, 3 H, Bi–CH_3), 4.05-4.08 (br, m, 20 H, thf) ppm.

^{1}H-NMR (300 MHz, MeCN-*d*$_3$): 1.74-1.89 (br, m, 20 H, thf), 1.99 (s, 3 H, Bi–CH_3), 3.60-3.76 (br, m, 20 H, thf) ppm.

^{1}H-NMR (300 MHz, Pyridine-*d*$_5$): 1.55-1.71 (br, m, 20 H, thf), 3.48 (s, 3 H, Bi–CH_3), 3.59-3.75 (br, m, 20 H, thf) ppm.

^{13}C{^{1}H}-NMR (126 MHz, CD_2Cl_2): δ = 25.95 (s, thf), 71.37 (s, thf), 88.7 (s, Bi–CH$_3$, detected *via* ^{1}H-^{13}C-HSQC-spectrum) ppm.

^{19}F-NMR (283 MHz, CD_2Cl_2): δ = –106 to –142 (br, m, SbF_6) ppm.

Elemental analysis: calculated for $[C_{21}H_{43}BiF_{12}O_5Sb_2]$ (1056.05 g/mol): C 23.88, H 4.10; found: C 23.60, H 3.968.

IR (ATR-Probe head): $\tilde{\nu}$ = 2980 (w, C–H), 2892 (w, C–H), 2041 (w, C–H), 1966 (w, C–H), 1461 (w, C–H), 1250 (w, C–O), 1016 (m, C–O), 922 (w, C–C), 830 (m, C–C), 744 (w, C–C), 651 (s, C–C or Sb–F) cm^{-1}.

1.3. Preparation of $[BiMe(py)_5][SbF_6]_2$ (2)

$[BiMe(thf)_5][SbF_6]_2$ (25 mg, 0.024 mmol, 1 eq.) was dissolved in pyridine (0.5 mL) and layered with a mixture of diethylether (5 mL) and *n*-pentane (2 mL). After 3 d at –30 °C colorless crystals of $[BiMe(py)_5][SbF_6]_2$ were obtained (23 mg, 0.021 mmol, 88%)

Direct Synthesis:

$BiMeCl_2$ (50 mg, 0.170 mmol, 1 eq.) was dissolved in pyridine (2 mL). $AgSbF_6$ (117 mg, 0.340 mmol, 2 eq.) was added and the reaction mixture was dried *in vacuo*. The remaining colorless solid was extracted with DCM (3 × 3 mL). The combined liquid phases were layered with *n*-pentane (12 mL). After 1 d at –30 °C, colorless crystals had formed, were isolated by filtration, and dried at 10^{-3} mbar (125 mg, 0.114 mmol, 67%). The product obtained *via* this route was confirmed to be identical with that obtained from $[BiMe(thf)_5][SbF_6]_2$ by ^{1}H NMR spectroscopy.

^{1}H-NMR (300 MHz, Pyridine-*d_5*): δ = 3.23 (s, 3 H, Bi–C*H_3*) ppm.

^{1}H-NMR (300 MHz, MeCN-*d_3*): δ = 2.05 (s, 3 H, Bi–C*H_3*), 7.50-7.54 (m, 10 H, 3,5-py), 7.92-7.98 (m, 5 H, 4-py), 8.67 (d, 10 H, 2,6-py) ppm.

^{13}C{^{1}H}-NMR (75 MHz, MeCN-*d_3*): δ = 85.39 (s, Bi–*C*H_3), 126.00 (s, 3,5-py), 140.44 (s, 4-py), 148.43 (s, 2,6-py) ppm.

^{19}F-NMR (283 MHz, MeCN-*d_3*): δ = –106 to –142 (br, m, Sb*F_6*) ppm.

Elemental analysis: calculated for [$C_{26}H_{28}BiF_{12}N_5Sb_2$] (1091.03 g/mol): C 28.62, H 2.59, N 6.42; found: C 28.28, H 2.65, N 6.23.

1.4. Preparation of [BiMe(OPEt$_3$)$_4$][SbF$_6$]$_2$ (3)

[BiMe(thf)$_5$][SbF$_6$]$_2$ (50 mg, 0.047 mmol, 1.0 eq.) was dissolved in acetonitrile (0.5 mL) and a solution of OPEt$_3$ (25 mg, 0.189 mmol, 4.0 eq.) in acetonitrile (0.5 mL) was added. After 5 min., all volatiles were removed under reduced pressure. The remaining solid was suspended in acetonitrile (1 mL), filtered, and the filtrate was layered with pentane (2 mL) and diethylether (10 mL). Storing the solution at –30 °C leads to the formation of colorless crystals after 4 d. Filtration and drying *in vacuo* yields [BiMe(OPEt$_3$)$_4$][SbF$_6$]$_2$ (44 mg, 0.036 mmol, 77%).

^{1}H-NMR (500 MHz, MeCN-*d_3*): δ = 1.14 (dt, $^3J_{PH}$ = 17.4 Hz, $^3J_{HH}$ = 7.7 Hz, 36 H, OP(CH$_2$–C*H_3*)$_3$), 1.57 (s, 3 H, Bi–C*H_3*), 1.88 (dq, $^2J_{PH}$ = 12.2 Hz, $^3J_{HH}$ = 7.7 Hz, 24 H, OP(C*H_2*–CH$_3$)$_3$) ppm.

^{13}C{^{1}H}-NMR (126 MHz, MeCN-*d_3*): δ = 5.92 (d, $^2J_{PC}$ = 4.9 Hz, OP(*C*H$_2$–CH3)$_3$), 19.58 (d, $^1J_{PC}$ = 65.1 Hz, OP(CH$_2$–*C*H3)$_3$), 91.75 (s, Bi–*C*H$_3$, detected *via* ^{1}H-^{13}C-HSQC-spectrum) ppm.

^{19}F-NMR (283 MHz, MeCN-*d_3*): δ = –106 to –142 (br, m, Sb*F_6*) ppm.

^{31}P{^{1}H}-NMR (122 MHz, MeCN-*d_3*) δ = 69.61 (s, $^1J_{31P\text{-}13C}$ = 65.4 Hz, O*P*(Et)$_3$) ppm.

Elemental analysis: calculated for [$C_{25}H_{63}BiF_{12}P_4O_4Sb_2$] (1232,15 g/mol): C 24.37, H 5.15; found: C 24.47, H 5.07.

1.5. Preparation of [BiMe(SPMe$_3$)$_2$(MeCN)$_2$][SbF$_6$]$_2$ (4-MeCN)

[BiMe(thf)$_5$][SbF$_6$]$_2$ (20 mg, 0.019 mmol, 1.0 eq.) was dissolved in acetonitrile (0.5 mL) and a solution of SPMe$_3$ (4 mg, 0.038 mmol, 2.0 eq.) in acetonitrile (0.5 mL) was added. After 5 min., all volatiles were removed under reduced pressure. The remaining solid was suspended in acetonitrile (1 mL), filtered, and the filtrate was layered with diethylether (4 mL). Storing the solution at –30 °C led to the formation of colorless crystals after 3 d. Filtration and drying *in vacuo* yielded [BiMe(SPMe$_3$)$_2$(MeCN)$_2$][SbF$_6$]$_2$ (10 mg, 0.010 mmol, 53%).

^{1}H-NMR (500 MHz, MeCN-*d_3*): δ = 2.06 (d, $^2J_{PH}$ = 13.8 Hz, 18 H, SP(C*H_3*)$_3$), 2.18 (s, 3 H, Bi–C*H_3*) ppm.

$^{13}C\{^1H\}$-NMR (75 MHz, MeCN-d_3): δ = 19.36 (d, $^1J_{PC}$ = 53.1 Hz, SP(CH$_3$)$_3$), 54.32 (s, Bi–CH$_3$, detected *via* ^{1}H-^{13}C-HSQC-spektrum) ppm.

^{19}F-NMR (283 MHz, MeCN-d_3): δ = –106 to –142 (br, m, SbF_6) ppm.

$^{31}P\{^1H\}$-NMR (122 MHz, MeCN-d_3) δ = 43.67 (s, $^1J_{31P\text{-}13C}$ = 53.3 Hz, SP(CH$_3$)$_3$) ppm.

Elemental analysis: calculated for [$C_{11}H_{27}BiF_{12}N_2P_2S_2Sb_2$] (993.90 g/mol): C 13.29, H 2.74, N 2.82, S 7.03; found: C 13.55, H 2.71, N 2.80, S 6.68.

1.6. Preparation of [BiMe(SePMe$_3$)$_4$][SbF$_6$]$_2$ · MeCN (5)

[BiMe(thf)$_5$][SbF$_6$]$_2$ (50 mg, 0.047 mmol, 1.0 eq.) was dissolved in acetonitrile (1 mL) and a solution of SePMe$_3$ (29 mg, 0.189 mmol, 4.0 eq.) in acetonitrile (1 mL) was added. After 5 min., all volatiles were removed under reduced pressure. The remaining yellow-brownish solid was suspended in acetonitrile (1 mL), filtered, and the yellow filtrate was layered with diethylether (4 mL). Storing the solution at –30 °C led to the formation of yellow crystals after 3 d. Filtration and drying *in vacuo* yielded [BiMe(SePMe$_3$)$_4$][SbF$_6$]$_2$ (46 mg, 0.034 mmol, 72%).

^{1}H-NMR (500 MHz, MeCN-d_3): δ = 2.10 (d, $^2J_{PH}$ = 13.9 Hz, 36 H, SeP(CH_3)$_3$), 2.23 (s, 3 H, Bi–CH_3) ppm.

$^{13}C\{^1H\}$-NMR (126 MHz, MeCN-d_3): δ = 20.76 (d, $^1J_{PC}$ = 48.6 Hz, SeP(CH$_3$)$_3$), 40.98 (s, Bi–CH$_3$, detected *via* ^{1}H-^{13}C-HSQC-spektrum) ppm.

^{19}F-NMR (283 MHz, MeCN-d_3): δ = –106 to –142 (br, m, SbF_6) ppm.

$^{31}P\{^1H\}$-NMR (122 MHz, MeCN-d_3) δ = 17.63 (s, $^1J_{31P\text{-}13C}$ = 48.8 Hz, $^1J_{31P\text{-}77Se}$ = 563.9 Hz, SeP(CH$_3$)$_3$) ppm.

Elemental analysis: calculated for [$C_{15}H_{42}BiF_{12}NP_4Se_4Sb_2$] (1356.77 g/mol): C 13.28, H 3.12, N 1.03; found: C 13.52, H 3.23, N 1.05. The crystalline compound contains 1 eq. of lattice-bound acetonitrile, as detected by single-crystal X-ray analysis and elemental analysis.

1.7. Preparation of [{BiMe(phen)$_2$}$_2$(μ-F)][SbF$_6$]$_3$ (6)

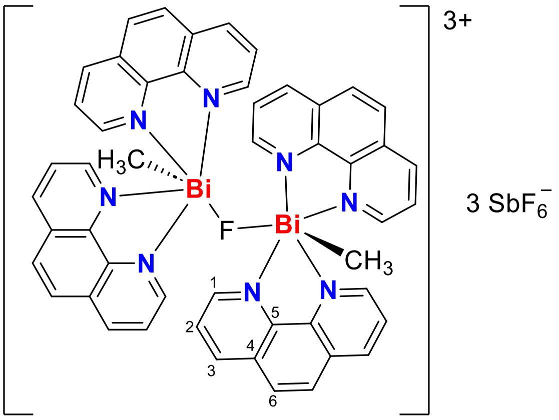

[BiMe(thf)$_5$][SbF$_6$]$_2$ (30 mg, 0.028 mmol, 2.0 eq.) was dissolved in acetonitrile (1 mL) and phenantronline (12 mg, 0.064 mmol, 4.5 eq.) was added. The product was crushed out by addition of diethylether (5 mL) and *n*-pentane (2 mL). To complete precipitation, the suspension was stored at –30 °C for 18 h. Filtration and drying *in vacuo* yielded [{BiMe(phen)$_2$}$_2$(μ-F)][SbF$_6$]$_3$ as a colorless solid (23 mg, 0.012 mmol, 86%).

^{1}H-NMR (500 MHz, MeCN-*d$_3$*): δ = 1.84 (s, 6 H, Bi–C*H*$_3$), 8.03 (dd, $^3J_{HH}$ = 4.9 Hz, $^3J_{HH}$ = 8.0 Hz, 8 H, phen-*H*2), 8.25 (s, 8 H, phen-*H*6), 8.88 (d, $^3J_{HH}$ = 8.4 Hz, 8 H, phen-*H*3), 9.20 (d, $^3J_{HH}$ = 4.4 Hz, 8 H, phen-*H*1) ppm.

^{13}C{^{1}H}-NMR (126 MHz, MeCN-*d$_3$*): δ = 73.42 (s, Bi–*C*H$_3$, detected *via* ^{1}H-^{13}C-HSQC spectrum), 126.82 (s, phen-*C*2), 128.93 (s, phen-*C*6), 132.24(s, phen-*C*4), 141.52(s, phen-*C*3), 144.87(s, phen-*C*5), 151.35 (s, phen-*C*1) ppm.

^{19}F-NMR (283 MHz, MeCN-*d$_3$*): δ = –106 to –142 (br, m, Sb*F*$_6$), –131.7 (br, s, μ-*F*) ppm.

Elemental analysis: calculated for [$C_{50}H_{38}Bi_2F_{19}N_8Sb_3$] (1895.12 g/mol): C 31.69, H 2.02, N 5.91; found: C 31.49, H 2.06, N 5.76.

1.8. Preparation of [{BiMe(bipy)$_2$}$_2$(μ-F)][SbF$_6$]$_3$ (7)

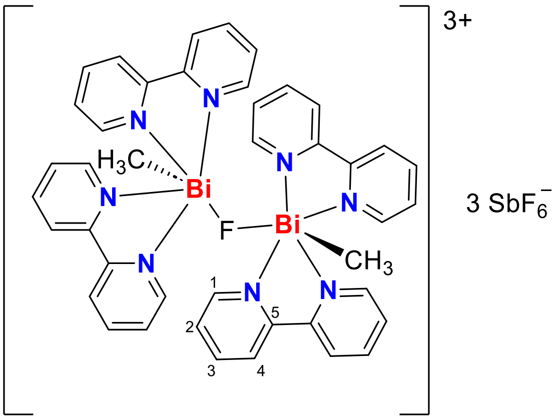

[BiMe(thf)$_5$][SbF$_6$]$_2$ (40 mg, 0.038 mmol, 2.0 eq.) was dissolved in acetonitrile (1 mL) and 2,2'-bipyridine (13 mg, 0.085 mmol, 4.5 eq.) was added. The product was crushed out by addition of diethylether (10 mL). To complete precipitation, the suspension was stored at –30 °C for 18 h. Filtration and drying *in vacuo* yields [{BiMe(phen)$_2$}$_2$(μ-F)][SbF$_6$]$_3$ as a colorless solid (30 mg, 0.017 mmol, 89%).

^{1}H-NMR (500 MHz, MeCN-*d$_3$*): δ = 1.67 (s, 6 H, Bi–C*H*$_3$), 7.77 (ddd, $^3J_{HH}$ = 7.4 Hz, $^3J_{HH}$ = 5.5 Hz, $^4J_{HH}$ = 1.0 Hz, 8 H, bipy-*H*3), 8.37 (ddd, 8 H, $^3J_{HH}$ = 7.9 Hz, $^3J_{HH}$ = 7.7 Hz, $^4J_{HH}$ = 1.4 Hz, bipy-*H*2), 8.52 (d, $^3J_{HH}$ = 8.1 Hz, 8 H, bipy-*H*4), 8.89 (d, $^3J_{HH}$ = 5.4 Hz, 8 H, bipy-*H*1) ppm.

^{13}C{^{1}H}-NMR (126 MHz, MeCN-*d$_3$*): δ = 71.64 (s, Bi–*C*H$_3$), 125.98 (s, bipy-*C*4), 128.36 (s, bipy-*C*3), 142.61 (s, bipy-*C*2), 150.70 (s, bipy-*C*1), 154.41 (s, bipy-*C*5) ppm.

^{19}F-NMR (283 MHz, MeCN-*d$_3$*): δ = –106 to –142 (br, m, Sb*F*$_6$), –129.5 (br, s, μ-*F*) ppm.

Elemental analysis: calculated for [C$_{42}$H$_{38}$Bi$_2$F$_{19}$N$_8$Sb$_3$] (1799.03 g/mol): C 28.04, H 2.13, N 6.23; found: C 27.70, H 2.28, N 6.37.

The SbF$_5$ that is released during the formation of **6** and **7** could not be detected by ^{19}F NMR spectroscopy, even within the presence of a strong donor like DMAP. Most likely, two SbF$_5$ moieties are transformed with an additional equivalent of L$_2$ to form [SbF$_4$(L$_2$)][SbF$_6$], as previously reported in the literature,[100,101] and removed during work-up.

2. Literature overview of pentagonal pyramidal coordinated bismuth as well as important structures of bismuth dications

The CCDC-Database contains over 1600 entries for octahedral coordinated bismuth centers.[102] Changing the search parameters to a pentagonal pyramid, only 63 entries are found.[102] But most of them show some type of additional secondary interaction, as mentioned in the main part. The remaining eight examples are shown in Figure S1.[69-74,103,104] In most cases (Figure S1, **I** to **VI**), the coordination polyhedron is formed by rigid multidentate ligands. This rarely leads to mononuclear species (Figure S1, **I**),[74] but more likely to di- (Figure S1, **II** and **III**),[72,104] tri- (Figure S1, **IV**),[73] or tetranuclear arrangements (Figure S1, **V** and **VI**)[71,103] in the solid state. Similar to that, the group around Gilliard published a phenylbismuth dication, stabilized by a carbodicarbene (Figure S1, **VII**).[70] One SbF_6^- molecule acts as a bidentate ligand as well as a bridging ligand between two bismuth centers. For compounds such as **V**, **VI**, or **VII**, it appears unlikely that the structure observed in the solid state is maintained in solution. A mononuclear structure with monodentate ligands was observed by Schleid *et al.* where the central bismuth atom is shielded by a bulky, *tri*anionic *closo*-borate, $[B_{12}H_{11}]^{3-}$, and coordinated by five water molecules in a plane (Figure S1, **VIII** (mentioned as compound **E** in the main part)).[69] **VIII** could not be isolated in pure form and a yield has not been reported, highlighting the challenges that are associated with the design, isolation, and detailed characterization of well-defined compounds showing a pentagonal pyramidal coordination geometry using only monodentate ligands.

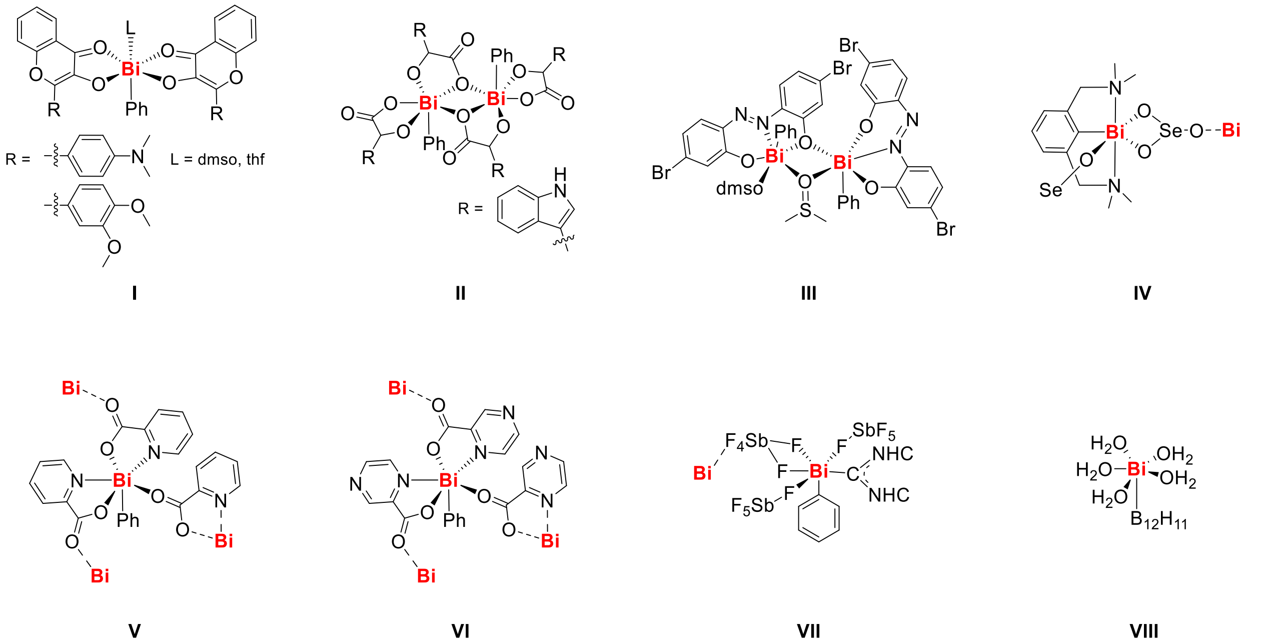

Figure S1. Bismuth compounds with a pentagonal pyramidal coordination geometry.

Moving on to cationic bismuth compounds it was shown, that they outnumber their neutral analogs when it comes to unusual coordination numbers and geometries.[50,51,105-107] With higher charges i.e. bismuth-dications, there is a wide variety of coordination numbers and geometries accessible (Figure S2, **IX**-**XIII** (four of these species are mentioned as compounds **A**-**D** in the main part)). Taking all interactions with solvent molecules or weakly coordinating anions (WCAs) into account, these range from a coordination number of nine, forming a tricapped trigonal prism (Figure S2, **IX**),[55,108] down to five, which corresponds to a (distorted) tetragonal pyramidal coordination geometry (Figure S2, **X**).[58,62] Examples with an coordination number of seven (Figure S2, **XI**) or eight (Figure S2, **XII**) are scarce, and are mostly found, when solvent molecules and / or the counteranions show bonding interactions with the central bismuth atom.[50,57,75,84,109]

For coordination number six, both structural motifs that were discussed before can be found for dicationic bismuth compounds. The octahedron again is more commonly found (Figure S2, **XIII**).[56,76,110–113] In the group of dicationic bismuth compounds, the example found by Gilliard and co-workers is the only example of a dicationic bismuth compound with a pentagonal pyramidal coordination polyhedron in the solid state (taking weak interactions with the counteranions into consideration; Figure S1, **VII**).[70]

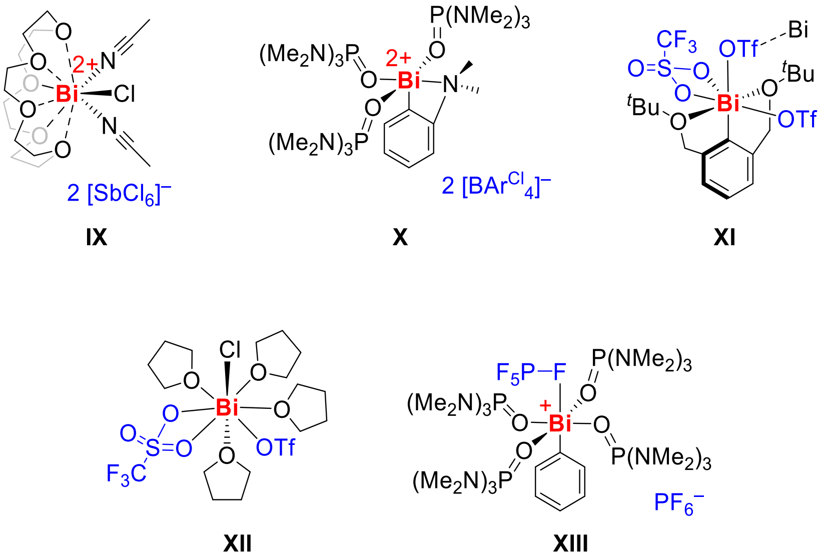

Figure S2. Examples of dicationic bismuth compounds with various coordination numbers and geometries. (Weakly) coordinating anions are shown in blue. Also see compound **VII** in Figure S1.

3. Discussion of the synthesis and characterization of [BiMe(py)$_5$][SbF$_6$]$_2$

The THF ligands around **1** can easily be replaced by stronger ligands e.g. simply by dissolving **1** in pyridine. The resulting product [BiMe(py)$_5$][SbF$_6$]$_2$ (**2**) is insoluble in DCM, so all NMR spectroscopic analyses were conducted in MeCN-d_3. The ^{1}H NMR spectrum is similar to the one recorded for **1**. There is one singlet for the methyl group at δ = 2.05 ppm (for reference: CH_3 in **1** in MeCNd_3: δ = 1.99 ppm), and three multiplets of the pyridine ligands between δ = 7.50 and 8.67 ppm. Comparing the signal intensity of the singlet and the multiplets confirms the coordination of five pyridine ligands. In the ^{13}C NMR spectrum the resonance for the methyl group is detected at δ = 85.39 ppm. The molecular structure of **2** in the solid state was confirmed *via* single-crystal X-Ray diffraction analysis (triclinic space group P$\bar{1}$, Z = 4). There are two molecules in the asymmetric unit, both show a central bismuth atom coordinated by five pyridine ligands in a plane and the methyl group on one side of the plane and one of two [SbF$_6$]$^-$ counterions on the other side. The shortest Bi–F distance is 3.199(2) or 3.292(2) Å (for the two crystallographically independent molecules in the asymmetric unit), which is significantly shorter than the sum of the van-der-Waals radii resulting in a contact ion pair in the solid state, based on distance criteria. Due to this weak Bi–F contact, the coordination geometry around bismuth results in a distorted pentagonal bipyramid with the F-atom and the methyl group in opposing apical positions. It appears somewhat counterintuitive to obtain a Bi–F contact in **2**, but not in **1**, since the pyridine ligands in **2** show a higher dipole moment (1.6 D) than the thf ligands in **1** (2.2 D). A closer analysis reveals that minor differences in steric demand may contribute to the scenario observed in the solid state: The shortest F-ligand contact in the solid state involves hydrodogen atoms of the thf and the pyridine ligands, respectively. The sp^3 (thf) vs sp^2 (pyridine) hybridization of the relevant carbon atoms leads to the relevant H···F interatomic distances of 2.32 Å to 2.58 Å in **1**, but 2.47 Å to 2.60 Å in **2**. In this context, the electronic situation revolving around potential Bi···F interactions was also analyzed in some more detail. The Bi–C bond lengths in **2** amount to 2.222(3) and 2.219(3) Å, which is slightly larger than the value observed for **1**, but still similar to other mono- and dicationic compounds. The elongation might be caused by the closer coordination of the SbF_6^- unit and subsequent population of the Bi–C σ*-orbital. NBO analyses of [BiMe(thf)$_5$]$^{2+}$ show that the second lone pair of each thf ligand is also engaged in n(O)→p(Bi) dative bonding, as deduced from second order perturbation analyses, which potentially contributes to the lack of interactions between the bismuth atom and the [SbF$_6$]$^-$ counteranions in **1**. For compound **2**, it also has to be pointed out that even though a Bi···F contact is evident in the solid state, this is most likely not persistent in solution, as suggested by ^{19}F NMR spectroscopy, showing a distinct multiplet (due to well-resolved $^1J_{FSb}$ coupling in species of octahedral symmetry), which is characteristic for non-coordinating SbF_6^-, while a broad singlet is typically observed for species that show relevant cation···[SbF$_6$]$^-$ contacts in solution.

4. Determination of acceptor numbers by the (modified) Gutmann-Beckett method

For an experimental determination of the Lewis acidity of **1** the traditional Gutmann-Beckett method (using $OPEt_3$),[81,82] and the modified version (using $SPMe_3$ and $SePMe_3$) were applied.[78] The acceptor numbers were calculated based on the ^{31}P-NMR chemical shift of the respective reaction mixtures of **1** with the Lewis base. The relevant formulae are:

$OPEt_3$: $AN = 2.21 \cdot (\delta_P - 41.0)$
$SPMe_3$: $AN = 6.41 \cdot (\delta_P - 29.2)$
$SePEt_3$: $AN = 5.71 \cdot (\delta_P - 7.8)$

NMR spectroscopic experiments were conducted *in situ* first, in a 1:1 stoichiometry, and secondly with several measurements increasing the amount of the Lewis base gradually from 0.5 to 2.0 equivalents.

General procedure for the *in situ* measurements

$[BiMe(thf)_5][SbF_6]_2$ (40 mg, 0.039 mmol, 1.0 eq.) was dissolved in CD_2Cl_2 (0.7 mL) and the required amount of the relevant donor was added (see Table S1). A capillary filled with 85% aqueous H_3PO_4 was added, and the sample was subjected to ^{31}P-NMR spectroscopic analysis. The results are summarized in Table S2.

Table S1. Amounts of the different Lewis bases for the (modified) Gutmann-Beckett method.

Equivalents of Lewis base	m ($OPEt_3$) [mg]	m ($SPMe_3$) [mg]	m ($SePMe_3$) [mg]
**0.5**	2.5	2.0	2.9
**0.6**	3.0	2.5	3.5
**0.7**	3.6	2.9	4.1
**0.8**	4.1	3.3	4.7
**0.9**	4.6	3.7	5.3
**1.0**	5.1	4.1	5.9
**1.5**	7.6	6.1	8.8
**2.0**	10	8.2	12

Table S2. ^{31}P-NMR chemical shift and calculated acceptor numbers for the measurement series.

Equivalents of Lewis base	$OPEt_3$		$SPMe_3$		$SePMe_3$	
	^{31}P-NMR chemical shift [ppm]	Acceptor number	^{31}P-NMR chemical shift [ppm]	Acceptor number	^{31}P-NMR chemical shift [ppm]	Acceptor number
0.5	79.21	84	43.55	92	22.87	86
0.6	79.16	84	43.55	92	22.96	87
0.7	79.12	84	43.57	92	22.98	87
0.8	78.97	84	43.50	92	23.09	87
0.9	78.65	83	43.77	93	23.10	87
1.0	78.32	83	43.88	94	23.06	87
1.5	78.21	82	43.58	92	22.75	85
2.0	77.23	80	42.16	83	21.16	76
4.0*	69.61	63	36.92	49	17.63	56

*These experiments were not part of the original reaction sequence, but added due to the composition of the isolated products (see above).

5. Synthesis and characterization of $BiMe^{2+}$ adducts with EPR_3 (E=O, S, Se; R=Me, Et)

To gain deeper insights into the coordination chemistry of bismuth dications, we aimed to find out which amount of each (modified) Gutmann-Beckett donor is needed to form an isolable product. Starting with one or two equivalents of each donor we layered each reaction mixture with *n*-pentane and analyzed the crystals that were formed, assuming these were the thermodynamic products. This leads to the following compounds: $[BiMe(OPEt_3)_4][SbF_6]_2$ (**3**), $[BiMe(SPMe_3)_2(SbF_6)_2]$ (**4**), and $[BiMe(SePMe_3)_4][SbF_6]_2$ (**5**). Single-crystal X-Ray diffraction shows a square pyramidal coordination geometry for **3** (monoclinic space group $P2_1/c$, Z = 2) with four donors in the basal plane and the methyl group in the apical position. For compound **5** (orthorhombic space group *Pbcn*, Z = 4), the arrangement of the neutral donors and the methyl group is analogous to that in **3**, but there is an additional weak contact (judged on the basis of distance criteria) with one of the $[SbF_6]^-$ counterions, leading to an overall distorted octahedral coordination geometry. Compound **4** (monoclinic space group $P2_1/n$, Z = 4) is coordinated by the methyl group and only two neutral donors, which results in additional bonding interactions between the central atom and both $[SbF_6]^-$ counteranions to give an overall square pyramidal coordination geometry. The methyl group is found in the apical position and the $SPMe_3$ units are found in a *cis* configuration. The quality of the crystallographic data was not sufficient for the discussion of further bonding parameters, but the connectivity is definite. Crystals of better quality were grown from an acetonitrile solution, leading to a replacement of the $[SbF_6]^-$ units by two acetonitrile ligands. This forms the complex $[BiMe(SPMe_3)_2(MeCN)_2][SbF_6]_2$ (**4-MeCN**, monoclinic space group $P2_1/m$, Z = 4, two molecules in the asymmetric unit). One of the counterions shows weak interactions with the bismuth atom (judged on the basis of distance criteria), resulting in a distorted octahedral coordination geometry.

A comparison of the bismuth–methyl bond lengths shows that those for the adducts **3** (2.280(9) Å), **4-MeCN** (2.210(9)-2.224(8) Å) and **5** (2.247(5) Å) are slightly elongated compared to the starting material **1** and closer to that of compound **2**. This could be due to the better σ-donor properties of the ligands (in the case of **3**) as well as a better HSAB pair fit between the ligands and the bismuth atom (in the case of **4**, **4-MeCN**, and **5**), compared to **1**.

For compound **3** with its $OPEt_3$ donor, a Bi–O bond length of 2.314(4)-2.329(3) Å is found, while the previously reported [Bi(Dipp)$_2$($OPEt_3$)$_2$(SbF_6)] shows a significantly longer Bi–O bond (2.437(2)-2.441(2) Å),[61] which might be due to higher steric repulsion caused by the Dipp substituent and due to the presence of four $OPEt_3$ donors in the case of **3**. Compared to literature-known compounds with dative bismuth oxygen bonds, both examples exhibit rather short bond lengths (reflecting the electron-deficient nature of the bismuth atoms), but are within the expected range (2.386(11)-2.879(15) Å).[57,114–116] In compound **5**, with its $SePMe_3$ donor, Bi–Se bond lengths of 2.9344(4)-2.9476(4) Å are found, which are longer than those reported in literature for similar compounds (2.7222(4)-2.7687(5) Å).[78,61] This was ascribed to the presence of four $SePMe_3$ donors and the resulting *trans* influence, which is not present in the literature compounds. In contrast, the Bi–S bond lengths in **4-MeCN** (2.6557(15)-2.6597(15) Å) are shorter than those reported in the literature (2.6700(8)-2.834(3) Å).[78,61] This might be due to the lower charge at the central bismuth atom in the literature examples and the fact that only two $SPMe_3$ donors are present in **4-MeCN**.

Repeating these reactions with a reasonable stoichiometry yields these products in medium to good yields (77% for **3**, 53% for **4-MeCN**, 72% for **5**). Subsequent NMR spectroscopic analyses are in agreement with the molecular structures assigned based on the single-crystal X-ray diffraction analyses. The ^{1}H NMR chemical shift for the methyl group is detected at δ = 1.57 ppm (for **3**), δ = 2.18 ppm (for **4-MeCN**) and δ = 2.23 ppm (for **5**).

While these products can be assumed to be formed in stepwise reactions, the titration of each donor to **1** did not lead to isolable intermediates featuring complex cations like $[BiMe(EPR_3)_{4-n}]^{2+}$ (E = O, S, Se; R = Me, Et; n = 1-3) and the formation of mixtures of compounds is suggested. As shown by Venugopal *et al.*, even the presence of one equivalent of $OPEt_3$ can lead to a dynamic situation with multiple compounds being present in an equilibrium scenario, especially when there are highly Lewis acidic compounds with multiple Lewis acidic sites.[84] Indeed, in the ^{31}P NMR spectra of our titration experiments of **1** with EPR_3 there is a significant signal broadening detected. The full-width at half maximum (FHWM) is 3-7 Hz for the free donors in acetonitrile, that value increases to 15-25 Hz when one or two equivalents of donor are present and decreases to 5-9 Hz when four equivalents have been added. Similarly high-resolution mass spectrometry of freshly prepared samples in a 1:1 stoichiometry revealed the presence of 2:1 and 3:1 (for $OPEt_3$ even 4:1) adducts within the same sample, underlining the formation of multiple adducts in equilibrium reactions.

6. Computational Details

All geometry optimizations were performed using the Gaussian16[117] suite of programs at the B3LYP+GD3/def2-TZVP level of theory.[118–123] The polarizable continuum model (PCM) has been used to model solvation in single point calculations.[124] A concentration correction has been added to Gibbs energies (1.894 kcal mol^{-1} for all computed systems, and 3.378 kcal mol^{-1} for THF).[125,126] Natural bond orbital (NBO) calculations were performed with the program NBO6 on optimized structures.[127] Multiwfn was utilized for the orbital composition analysis with Mulliken partition.[128,129] Intrinsic bond orbital analyses were performed with the IBO program suite.[130]

Fluoride ion affinities (FIA) were computed with the $SiMe_3F$ anchor point,[131] and using the solvation correction approach for charged species.[132]

Bi–O bonding in $[BiMe(thf)_5]^{2+}$

The analysis of the Bi–O bonding interactions in $[BiMe(thf)_5]^{2+}$ using the electron localization function (ELF) shows a near-spherical distribution of electron density around the oxygen atoms and relatively low minimum ELF values of ca. 0.15-0.20 along Bi–O bond paths. In addition, NBO analyses suggest the absence of covalent Bi–O bonds, but the presence of dative interactions according to second order perturbation analyses with n(O)→p(Bi) interaction energies of 25-33 kcal/mol. Altogether, these investigations suggest dative bonding interactions between O and Bi with significant ionic contributions.

Mapping electrostatic potential distribution in $[BiMe]^{2+}$

The electrostatic potential (ESP) distribution of $[BiMe]^{2+}$ was determined using the approach of analyzing the total electron density (determined using the self-consistent field method) mapped with ESP, as incorporated in the GaussView program. The result is shown in Figure S3, demonstrating that the highest ESP is found in the plane perpendicular to the Bi–C bond. Thus, not only a (hypothetical) purely covalent bonding situation, but also a (hypothetical) purely electrostatically controlled bonding situation would favor the coordination of ligands in the plane perpendicular to the Bi–C bond.

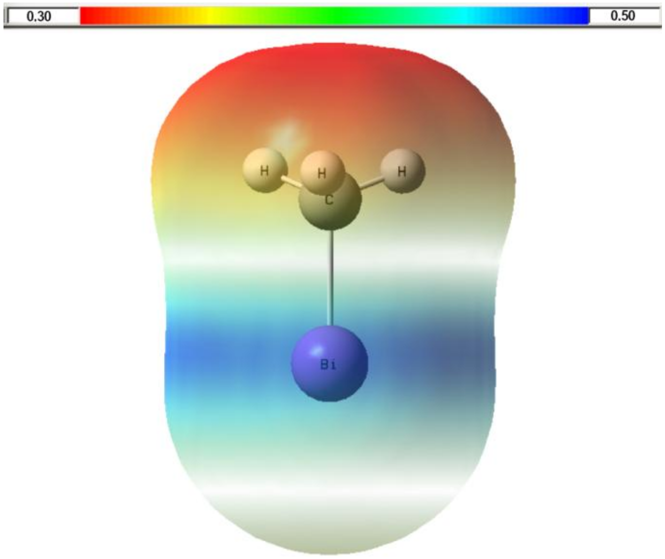

Figure S3. Electron density from total SCF density (isovalue = 0.0004) mapped with ESP for $[BiMe]^{2+}$.

7. Fluoride ion affinities (FIAs)

Tables S3 and S4 show the FIAs calculated with the $SiMe_3F$ anchor point, modeling the solvation by varying solvents with the PCM. The values obtained with the B3LYP functional (Table S3) and the BP86 functional (Table S4) show that the B3LYP approach predicts a slightly smaller difference in the FIA of the bismuth compounds and SbF_5. Both approaches predict a higher FIA for the bismuth systems in non- or weakly-polar solvents and are in agreement with experimental observations.

At the B3LYP/def2-tzvp/PCM(solvent=tetrahydrofuran) level of theory, the FIA of $[BiMe(thf)_4]^{2+}$ amounts to 279 kJ·mol^{-1} with $[BiMeF(thf)_4]^{+}$ as the product and to only 277 kJ·mol^{-1} with $[Bi_2Me_2F(thf)_8]^{3+}$ as the product, in agreement with experimental observations.

Table S3. Fluoride ion affinities (values in kJ · mol^{-1}) calculated with the $SiMe_3F$ anchor point, using the B3LYP functional, the def2-tzvp basis set, and solvation by varying solvents modeled in the PCM.

	**FIA (B3LYP/def2-tzvp)**					
	gas phase	pentane	Et_2O	CH_2Cl_2	THF	MeCN
$[BiMe(phen)_2]^{2+}$	944	589	361	269	292	205
2 $[BiMe(phen)_2]^{2+}$	800	582	418	350	362	303
SbF_5	524	446	390	366	371	350

Table S4. Fluoride ion affinities (values in kJ · mol^{-1}) calculated with the $SiMe_3F$ anchor point, using the BP86 functional, the def2-tzvp basis set, and solvation by varying solvents modeled in the PCM.

	**FIA (BP86/def2-tzvp)**					
	gas phase	pentane	Et_2O	CH_2Cl_2	THF	MeCN
$[BiMe(phen)_2]^{2+}$	941	588	361	269	286	207
2 $[BiMephen)_2]^{2+}$	810	594	431	364	377	319
SbF_5	513	435	380	358	362	342

Table S5. Gibbs free energies (not concentration-corrected) for complexes with THF solvent model (PCM).

compound	ΔG [hartree]
$[BiMe]^{2+}$	-254.132576
$[BiMe(thf)]^{2+}$	-486.663023
$[BiMe(thf)_2]^{2+}$	-719.168464
$[BiMe(thf)_3]^{2+}$	-951.651709
$[BiMe(thf)_4]^{2+}$	-1184.126697
$[BiMe(thf)_5]^{2+}$ (PPY-6)	-1416.601134
$[BiMe(thf)_5]^{2+}$ (OC-6)	-1416.586434
$[BiMe(thf)_6]^{2+}$	-1649.056967
$[BiMe(thf)_5][SbF_6]_2$ (PPY-6)*	-3096.63072
THF	-232.466083

*: no solvent model

Table S6. Thermal correction to enthalpy (H_{corr}) for compounds without a solvation model.

compound	H_{corr} **[hartree], B3LYP**	H_{corr} **[hartree], BP86**
$[BiMe(phen)_2]^{2+}$	0.407557	0.396969
$[BiFMe(phen)_2]^{+}$	0.410302	0.399562
$[Bi_2FMe_2(phen)_4]^{3+}$	0.81997	0.798566
SbF_5	0.01937	0.019004
$[SbF_6]^{-}$	0.022484	0.022068
$SiMe_3F$	0.122185	0.119355
$[SiMe_3]^{+}$	0.117284	0.112592
F^{-}	0.00236	0.00236

Table S7. Electronic energies (E_0) in hartree, as obtained from single point calculations with the B3LYP functional and a PCM solvent model, using the geometry from geometry-optimization in the gas phase.

	**E_0 (B3LYP, PCM solvent model)**					
	gas phase*	pentane	Et_2O	CH_2Cl_2	THF	MeCN
$[BiMe(phen)_2]^{2+}$	-1397.469519	-1397.973581	-1398.03417	-1398.058945	-1398.054338	-1398.076446
$[BiFMe(phen)_2]^{+}$	-1497.70296	-1498.139244	-1498.156376	-1498.163576	-1498.16223	-1498.168716
$[Bi_2FMe_2(phen)_4]^{3+}$	-2895.117677	-2896.112207	-2896.21432	-2896.255572	-2896.24789	-2896.284895
SbF_5	-739.838796	-739.8603756	-739.8626813	-739.8639285	-739.8636816	-739.8649291
$[SbF_6]^{-}$	-839.912256	-839.9716721	-839.9962585	-840.0061618	-840.0043422	-840.0129341
$SiMe_3F$	-509.196055	-509.3194319	-509.3205468	-509.3211117	-509.3210015	-509.3215518
$[SiMe_3]^{+}$	-408.948489	-409.1056766	-409.1324806	-409.1435029	-409.1414626	-409.1511698
F^{-}	-99.873974	-99.94080261	-99.98444856	-100.0019739	-99.99876423	-100.0138532

*ΔH value from gas phase geometry-optimized structure is given.

Table S8. Electronic energies (E_0) in hartree, as obtained from single point calculations with the BP86 functional and a PCM solvent model, using the geometry from geometry-optimization in the gas phase.

	**E_0 (BP86, PCM solvent model)**					
	gas phase*	pentane	Et_2O	CH_2Cl_2	THF	MeCN
$[BiMe(phen)_2]^{2+}$	-1397.562005	-1398.054574	-1398.114594	-1398.13906	-1398.134516	-1398.15629
$[BiFMe(phen)_2]^{+}$	-1497.792069	-1498.217394	-1498.23444	-1498.241604	-1498.240265	-1498.246716
$[Bi_2FMe_2(phen)_4]^{3+}$	-2895.304151	-2896.275987	-2896.377781	-2896.418846	-2896.411206	-2896.447963
SbF_5	-739.885669	-739.9065247	-739.9084592	-739.9095072	-739.9092996	-739.9103489
$[SbF_6]^{-}$	-839.952633	-840.0115049	-840.0361388	-840.0460575	-840.0442366	-840.0528237
$SiMe_3F$	-509.188832	-509.3093319	-509.310404	-509.310947	-509.3108411	-509.31137
$[SiMe_3]^{+}$	-408.947921	-409.0999785	-409.1263562	-409.1371653	-409.1351664	-409.1446686
F^{-}	-99.871709	-99.93851509	-99.98214558	-99.99966472	-99.99645621	-100.0115399

*ΔH value from gas phase geometry-optimized structure is given.

8. NMR Spectra

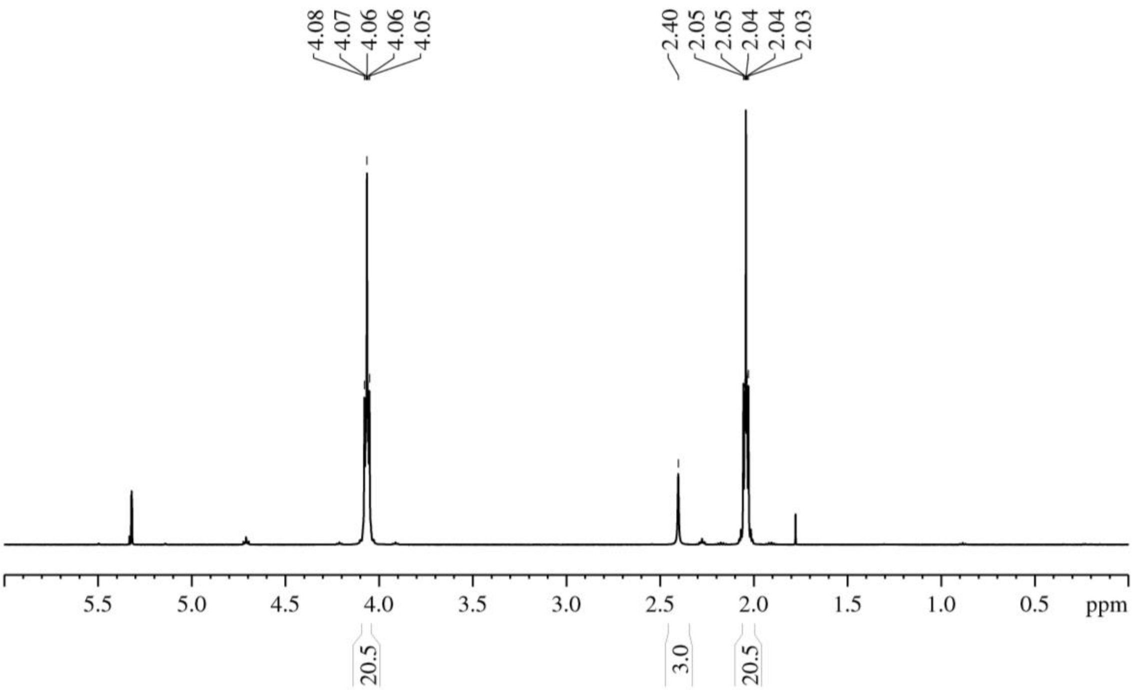

Figure S4. ^{1}H-NMR spectrum of **1** in CD_2Cl_2.

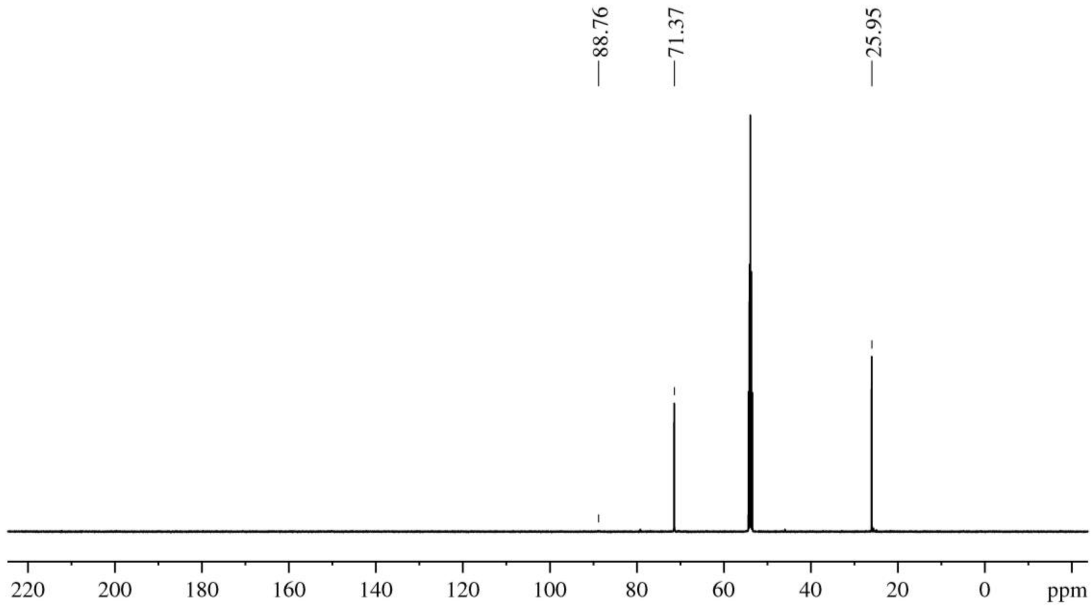

Figure S5. $^{13}C\{^{1}H\}$-NMR spectrum of **1** in CD_2Cl_2.

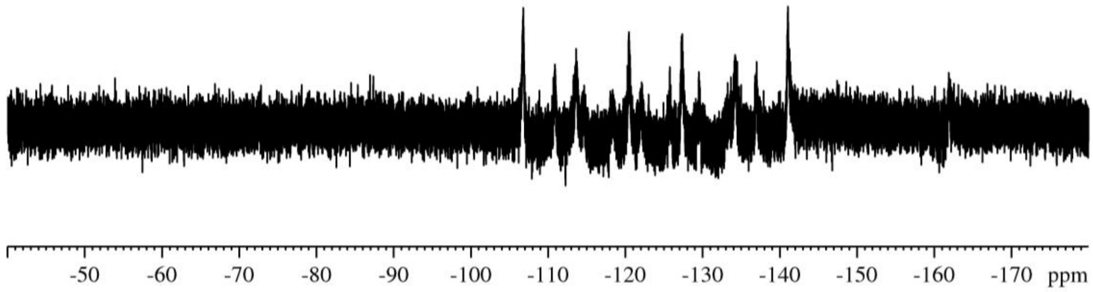

Figure S6. ^{19}F-NMR spectrum of **1** in CD_2Cl_2.

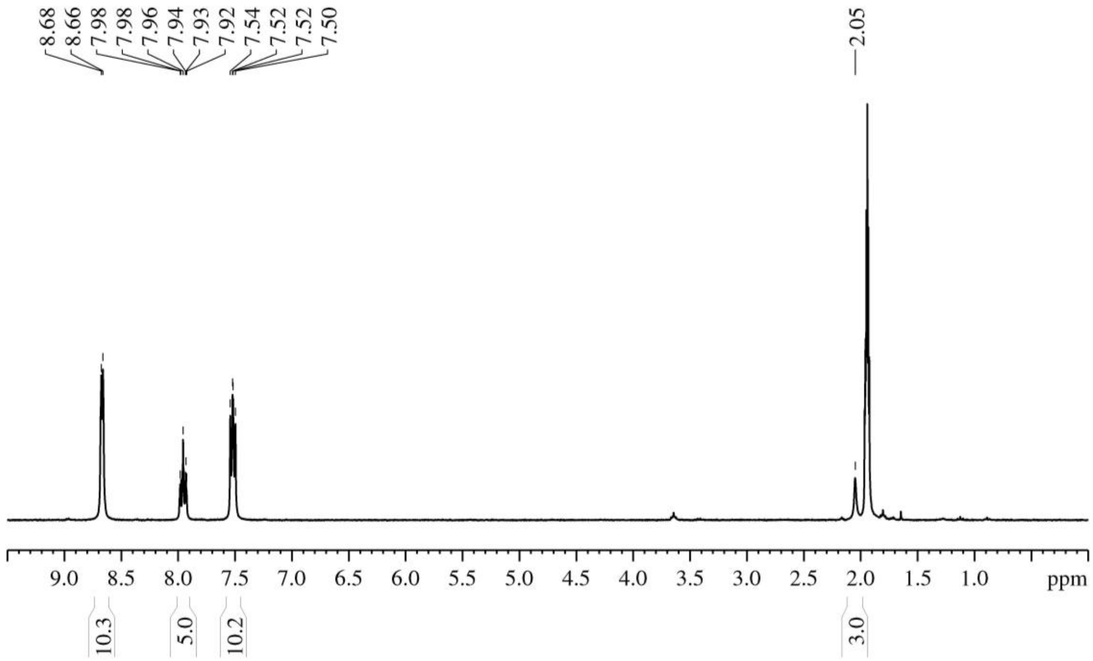

Figure S7. ^{1}H-NMR spectrum of **2** in CD_3CN.

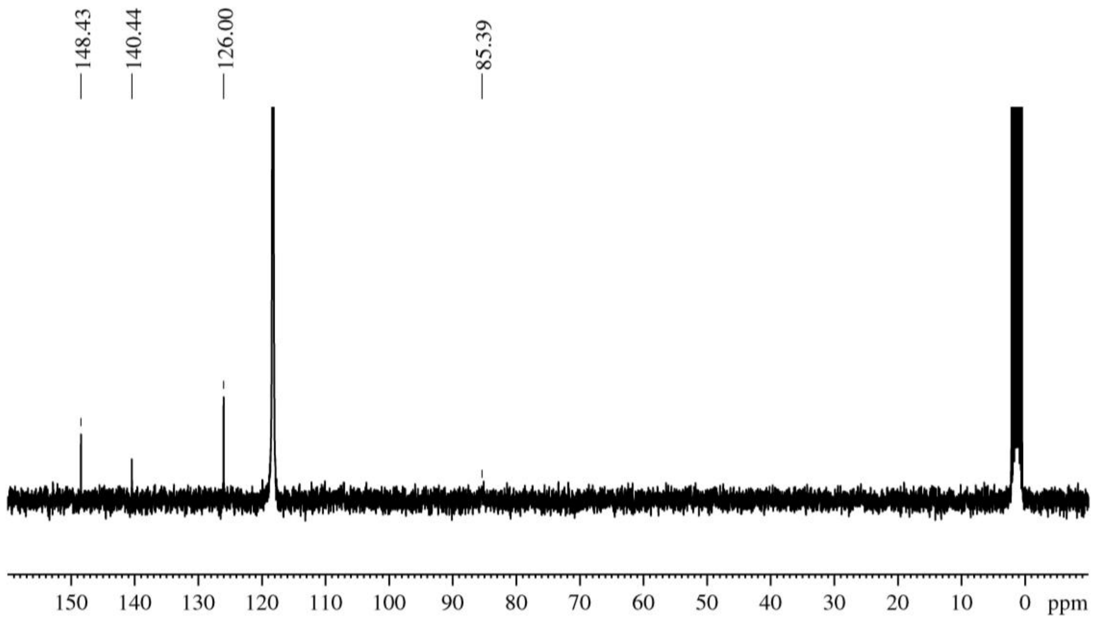

Figure S8. $^{13}C\{^{1}H\}$-NMR spectrum of **2** in CD_3CN.

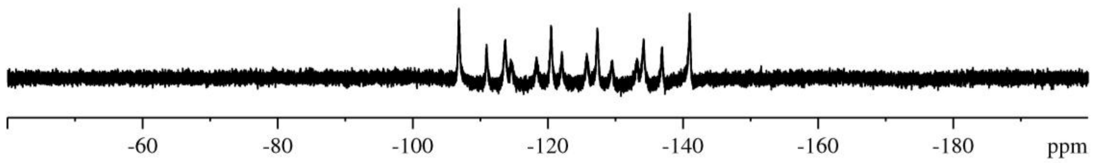

Figure S9. ^{19}F-NMR spectrum of **2** in CD_3CN.

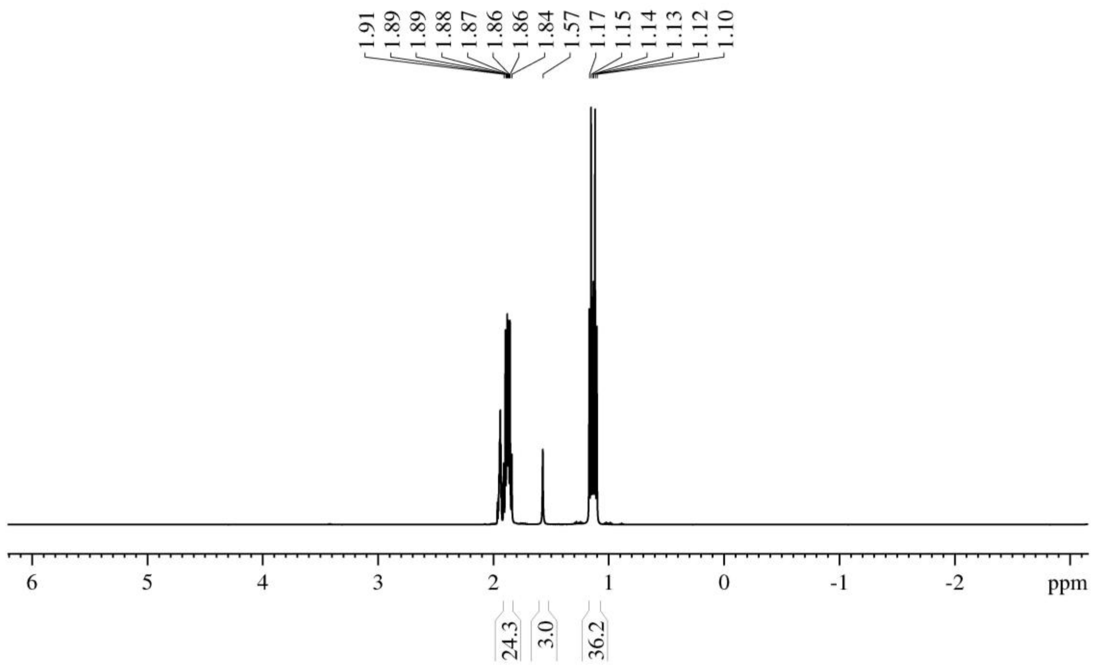

Figure S10. ^{1}H-NMR-spectrum of **3** in CD_3CN.

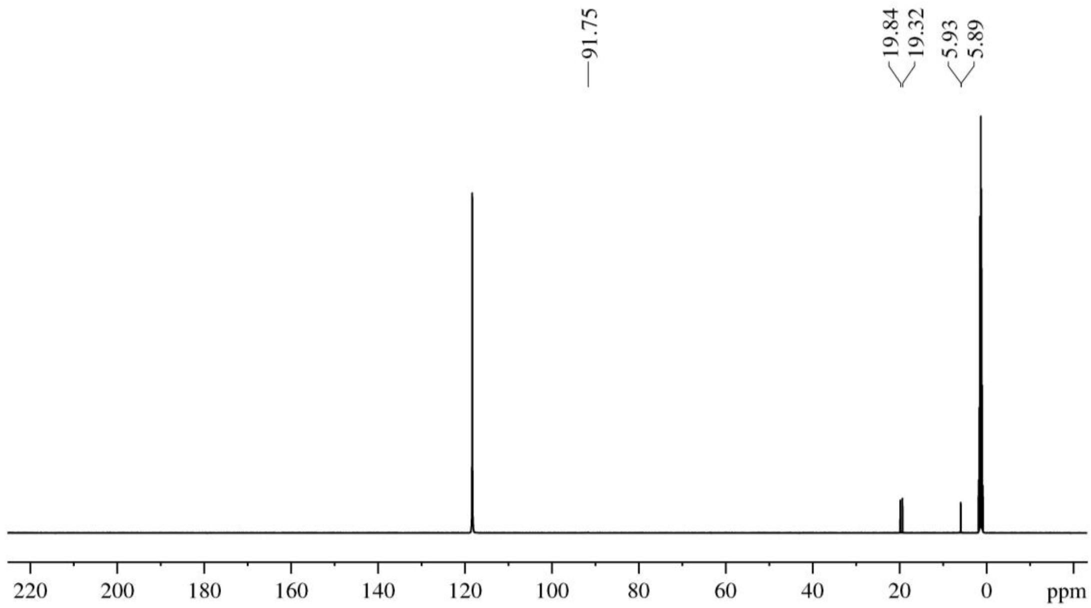

Figure S11. ^{13}C-NMR-spectrum of **3** in CD_3CN.

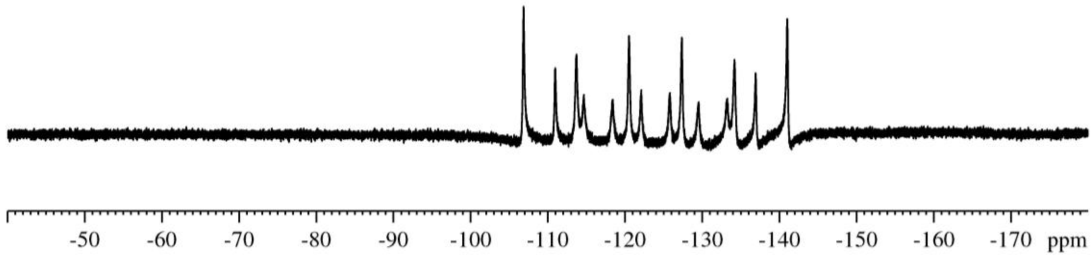

Figure S12. ^{19}F-NMR-spectrum of **3** in CD_3CN.

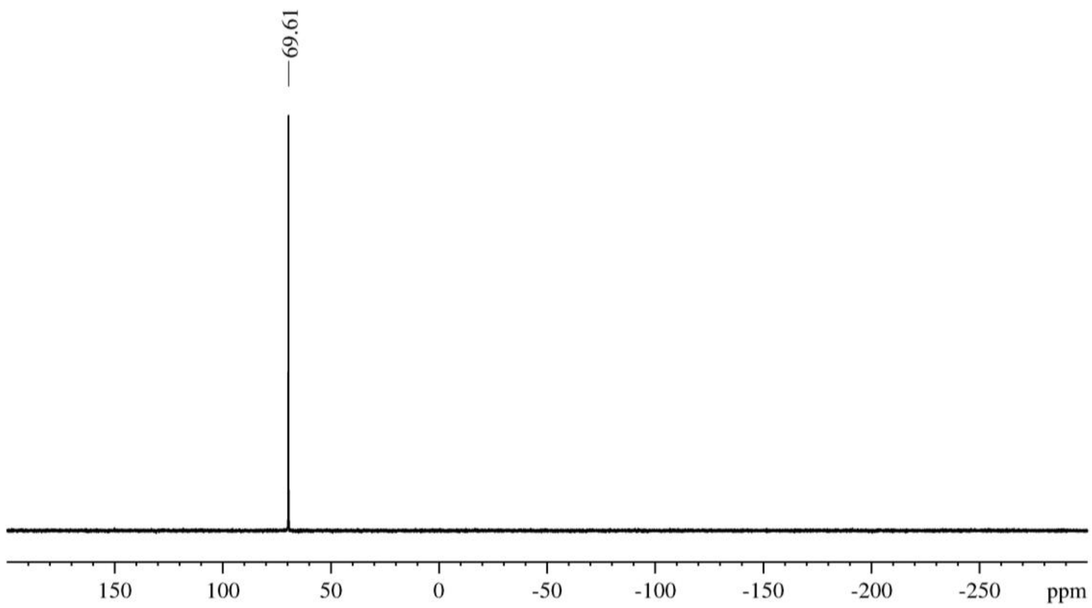

Figure S 13. ^{31}P-NMR-spectrum of **3** in CD_3CN.

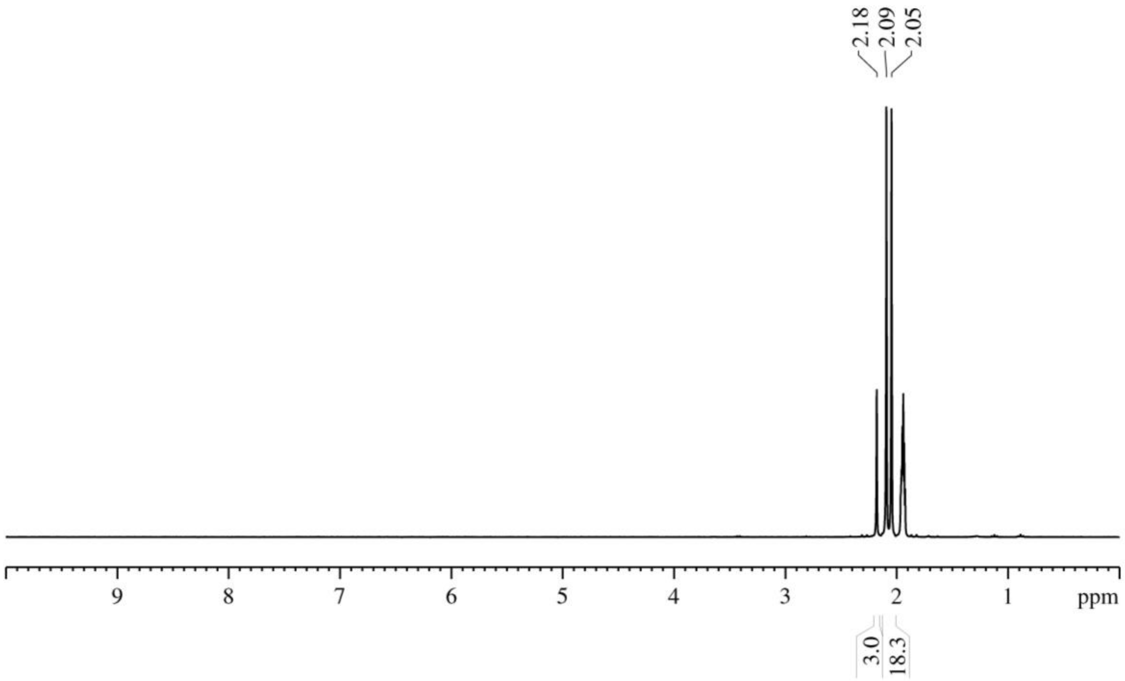

Figure S 14. ^{1}H-NMR-spectrum of **4-MeCN** in CD_3CN.

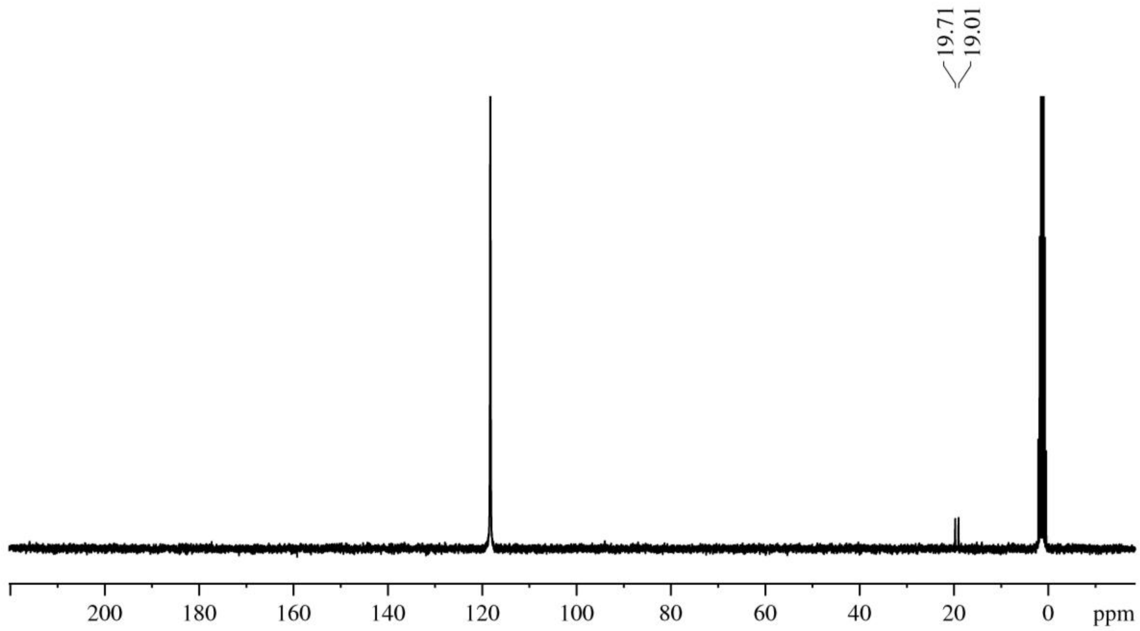

Figure S 15. $^{13}C\{^{1}H\}$-NMR spectrum of **4-MeCN** in CD_3CN.

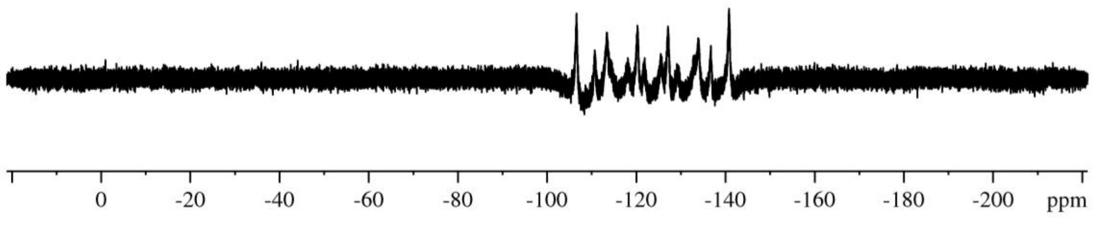

Figure S 16. ^{19}F-NMR spectrum of **4-MeCN** in CD_3CN.

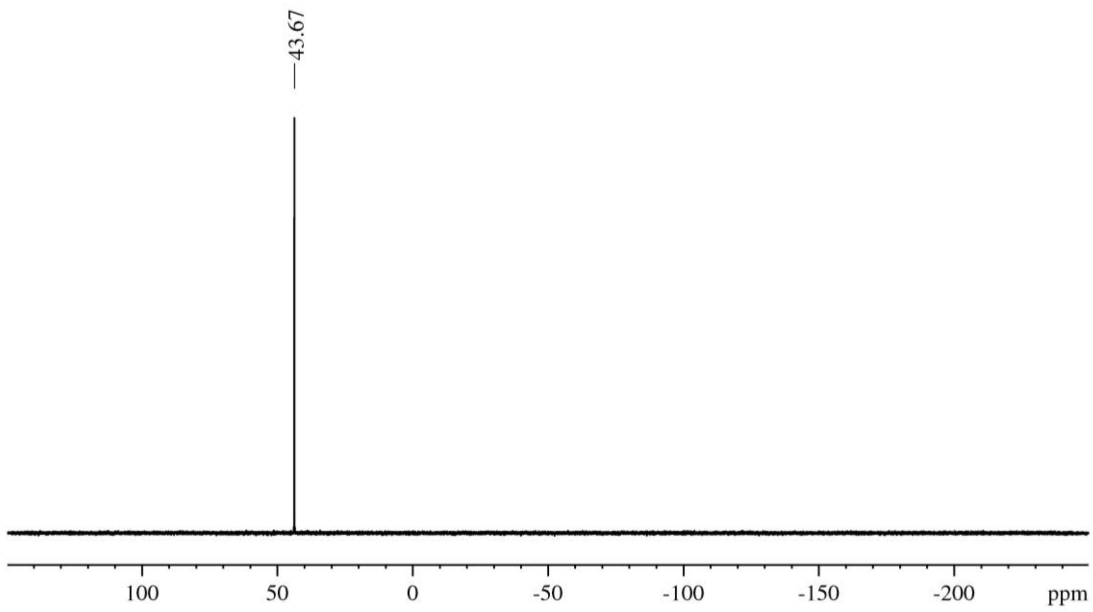

Figure S17. $^{31}P\{^1H\}$-NMR spectrum of **4-MeCN** in CD_3CN.

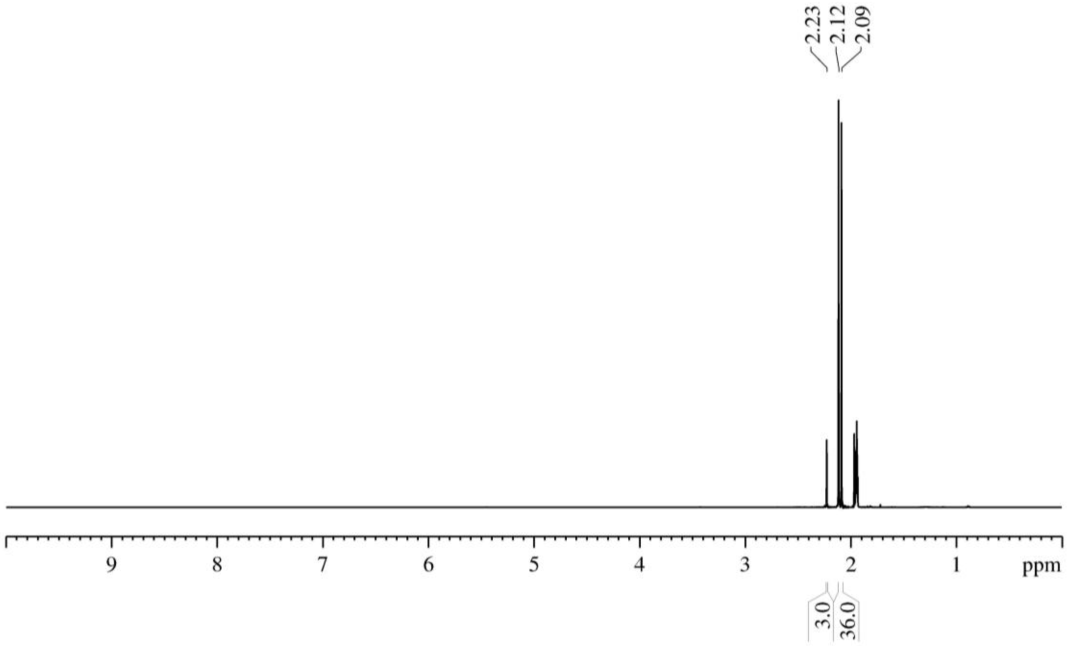

Figure S18. ^1H-NMR-spectrum of **5** in CD_3CN.

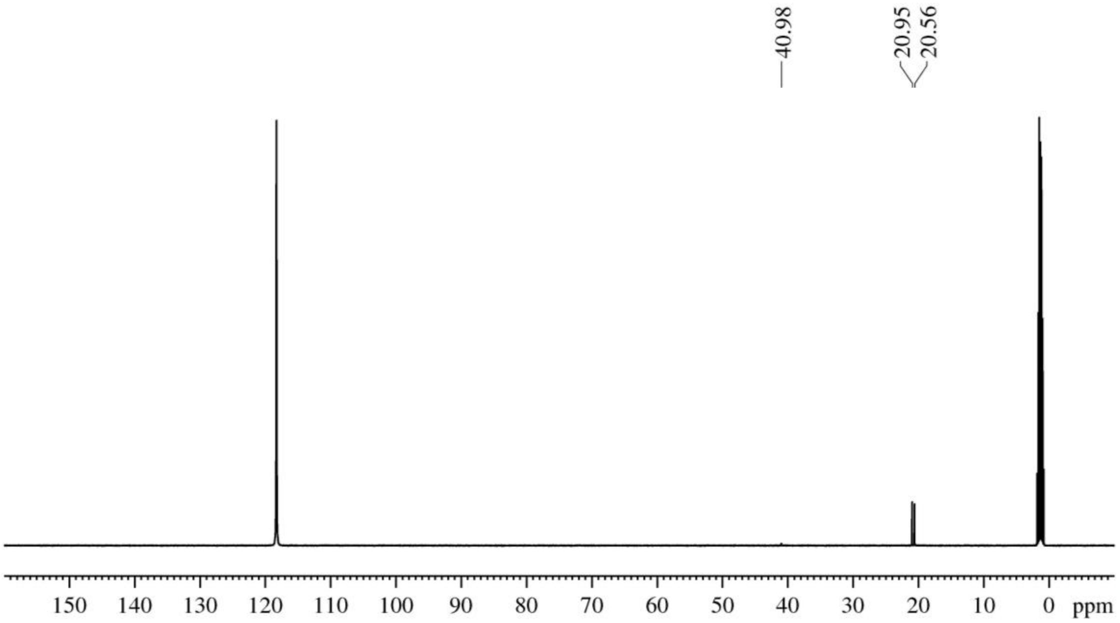

Figure S 19. $^{13}C\{^{1}H\}$-NMR spectrum of **5** in CD_3CN.

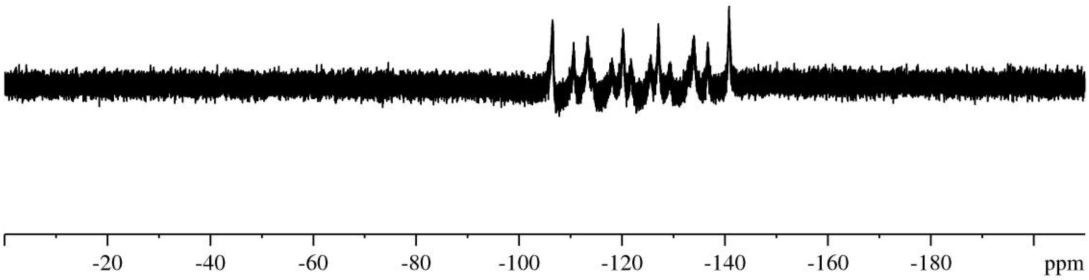

Figure S 20. ^{19}F-NMR spectrum of **5** in CD_3CN.

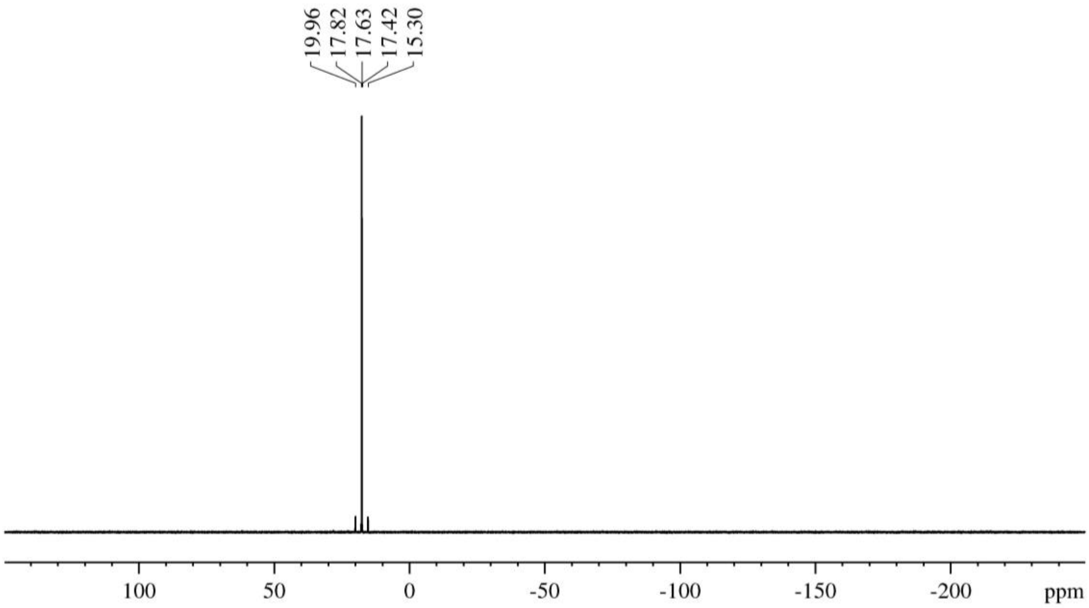

Figure S 21. $^{31}P\{^{1}H\}$-NMR spectrum of **5** in CD_3CN.

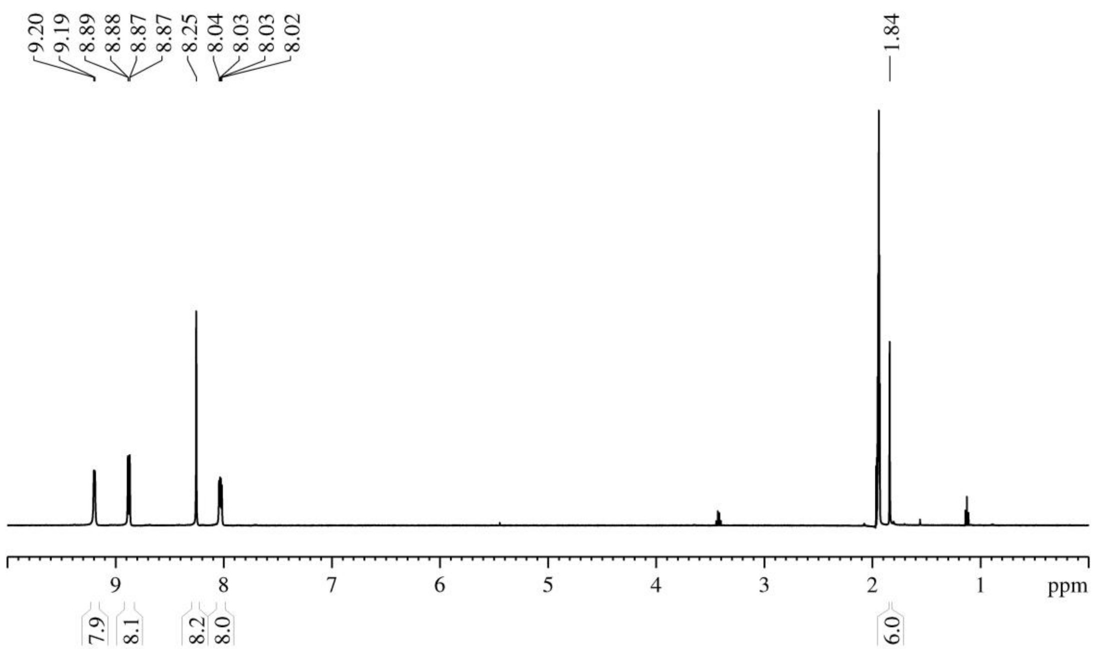

Figure S22. ^{1}H-NMR spectrum of **6** in CD_3CN.

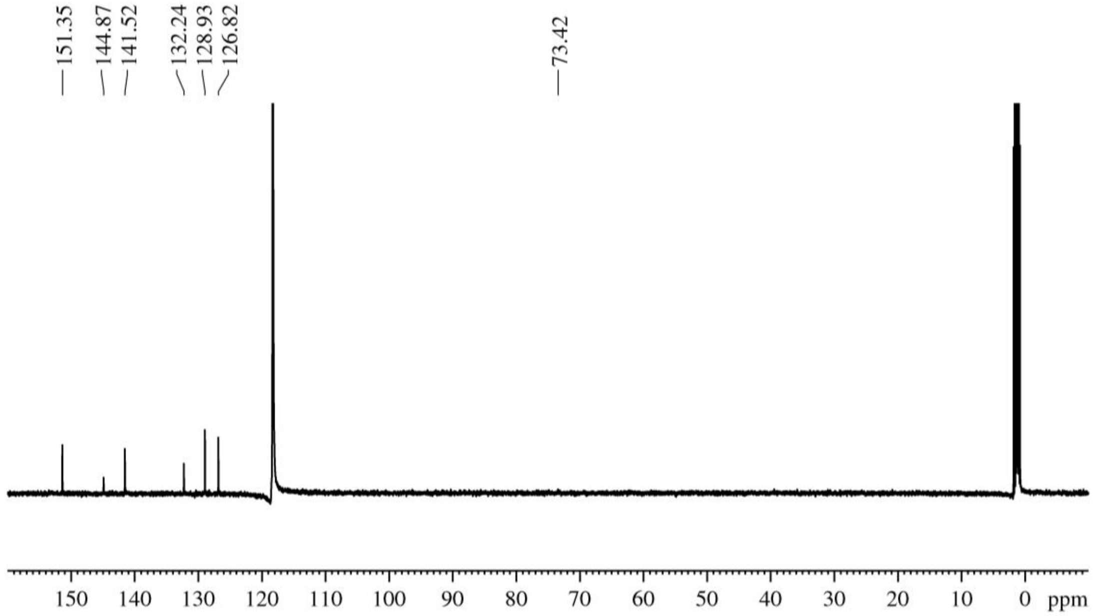

Figure S23. $^{13}C\{^{1}H\}$-NMR spectrum of **6** in CD_3CN.

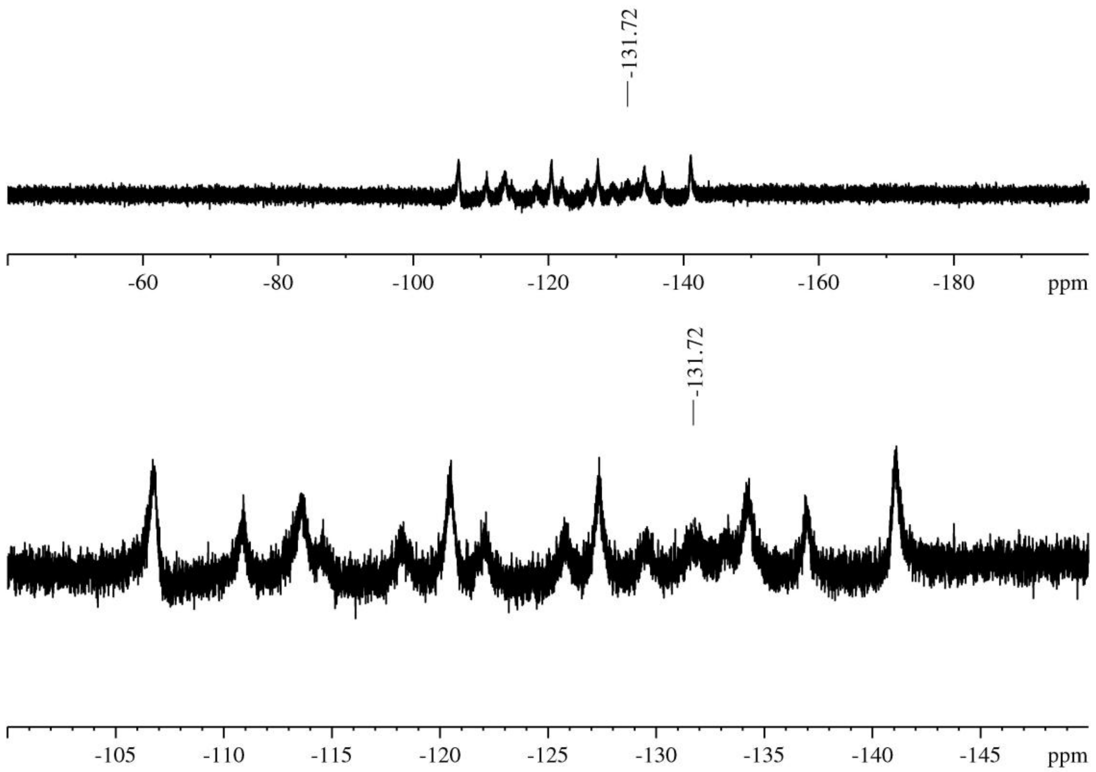

Figure S24. Top: ^{19}F-NMR spectrum of **6** in CD_3CN. Bottom: Enlargement of the area between –105 to –145 ppm.

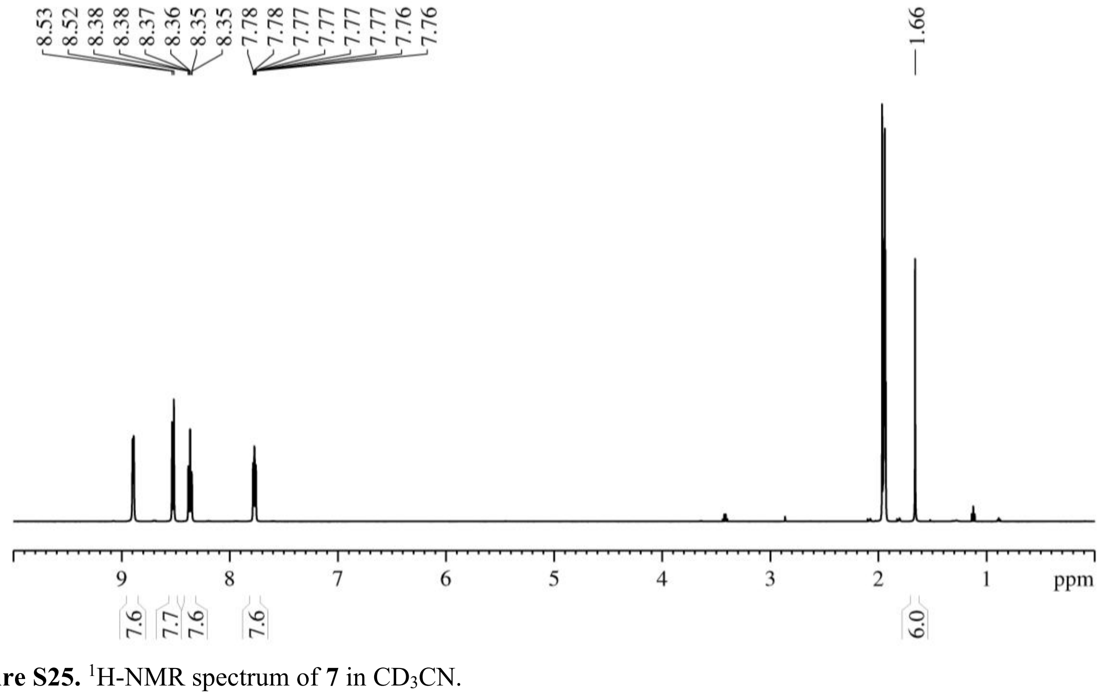

Figure S25. ^{1}H-NMR spectrum of **7** in CD_3CN.

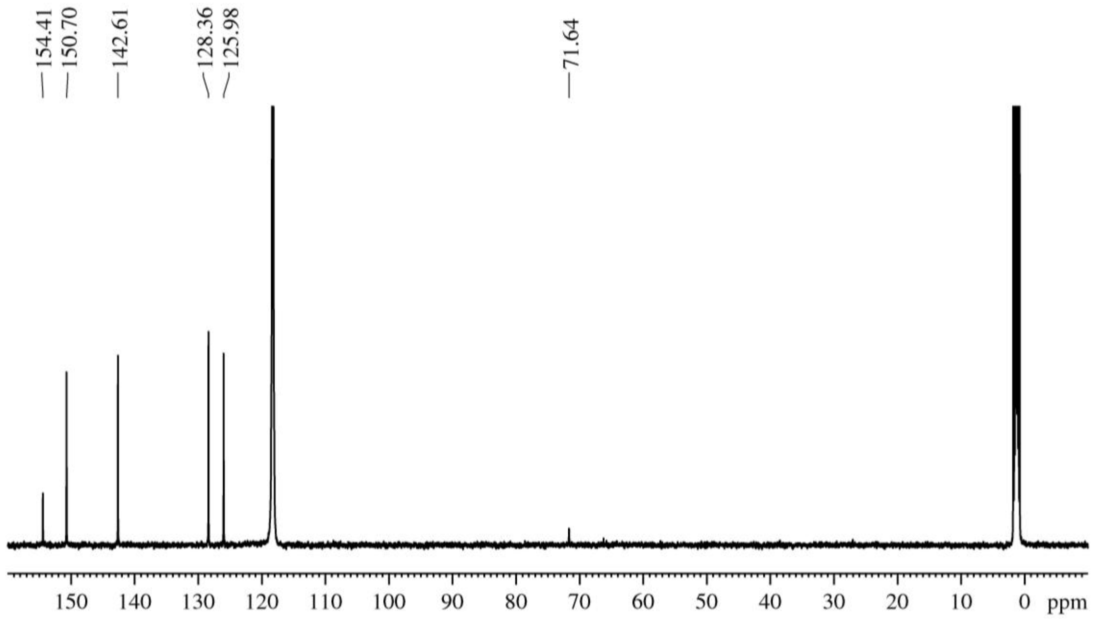

Figure S26. $^{13}C\{^{1}H\}$-NMR spectrum of **7** in CD_3CN.

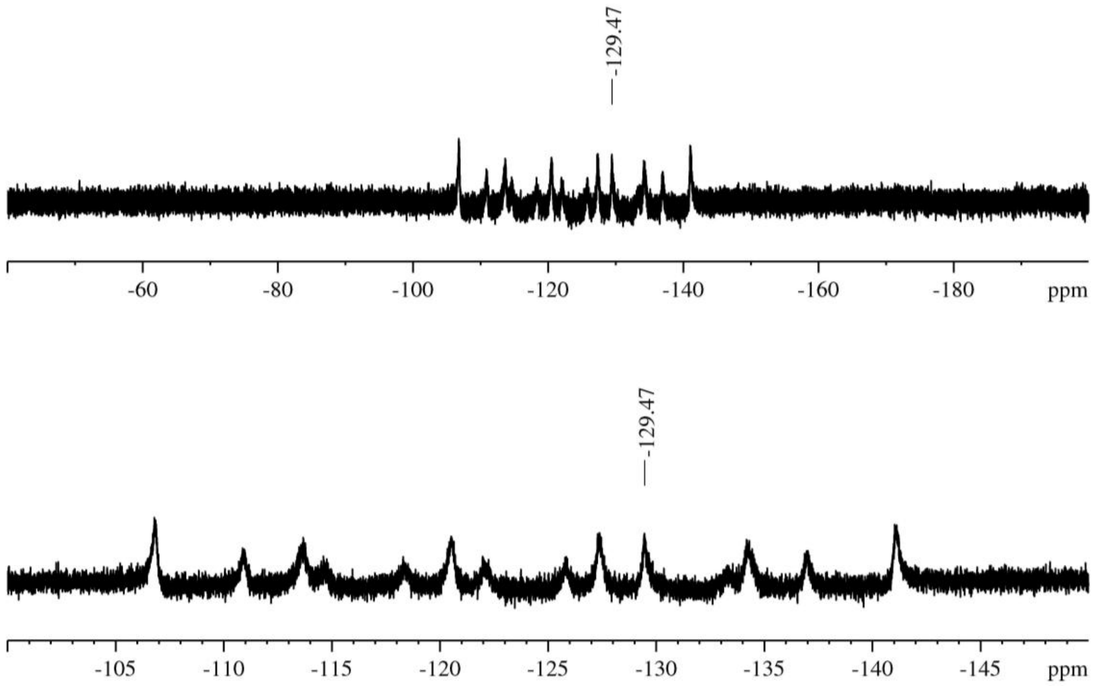

Figure S27. ^{19}F-NMR spectrum of **7** in CD_3CN. Bottom: Enlargement of the area between –105 to –145 ppm.

9. High-resolution mass spectrometry (1:1 mixtures of 1 and GB-donors)

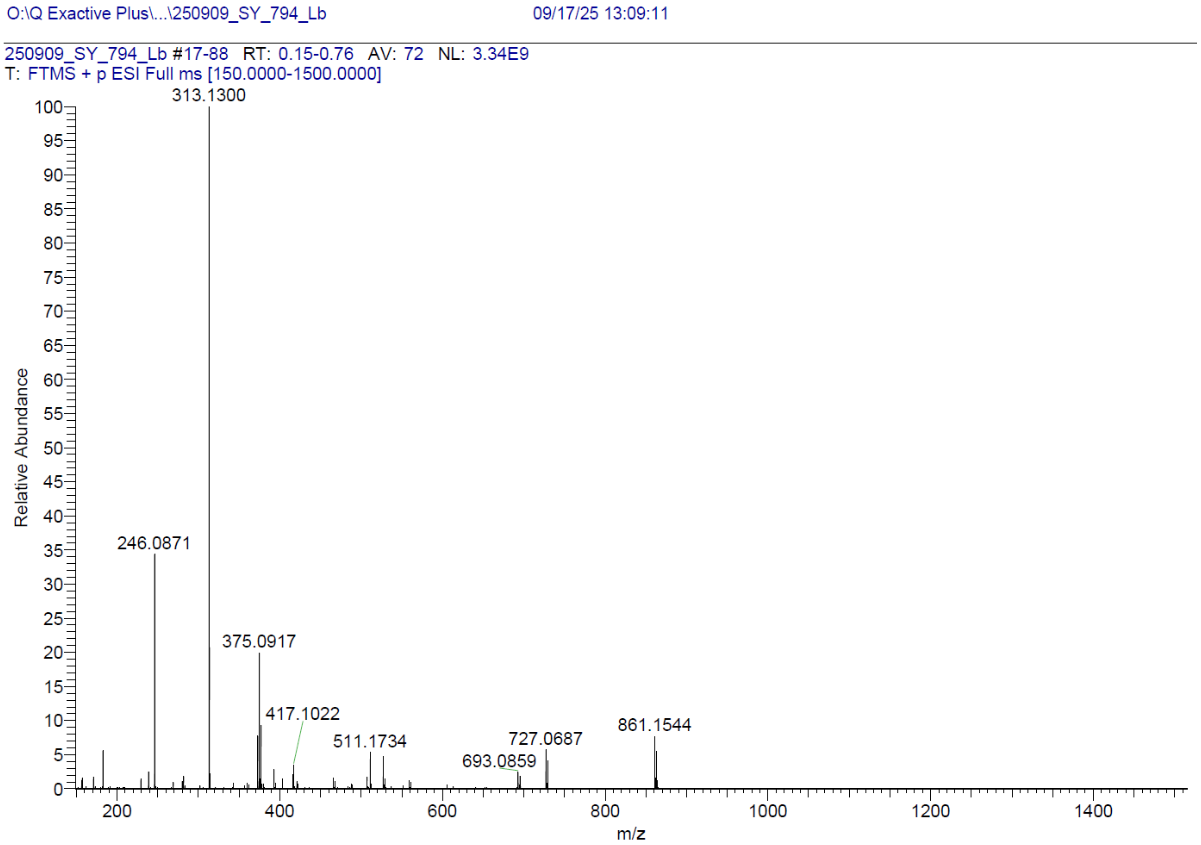

Figure S28: HRMS-ESI spectrum of the 1:1 mixture of $OPEt_3$ and **1** in MeCN.

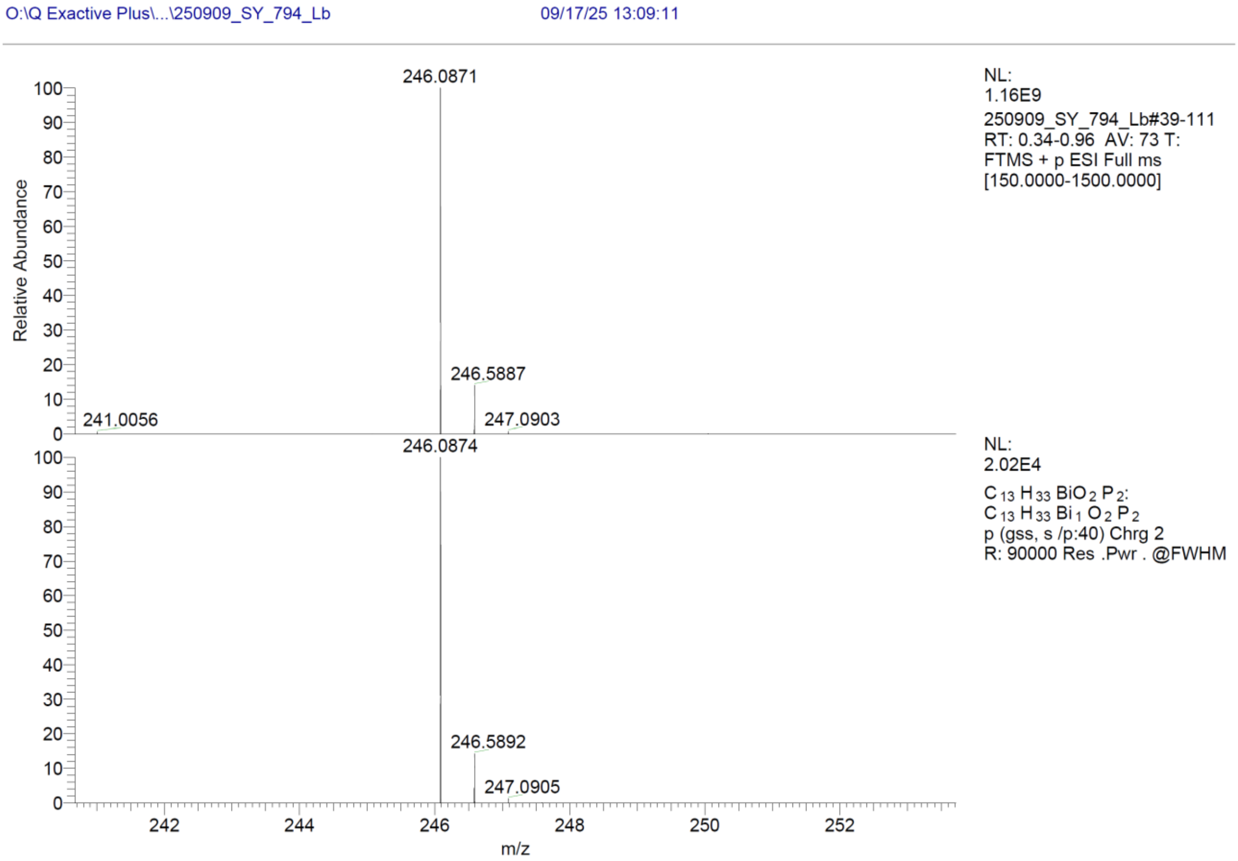

Figure S29: Top: HRMS-ESI spectrum of the 1:1 mixture of $OPEt_3$ and **1** in MeCN at m/z = 246; bottom: simulation of the signal corresponding to $[BiMe(OPEt_3)_2]^{2+}$.

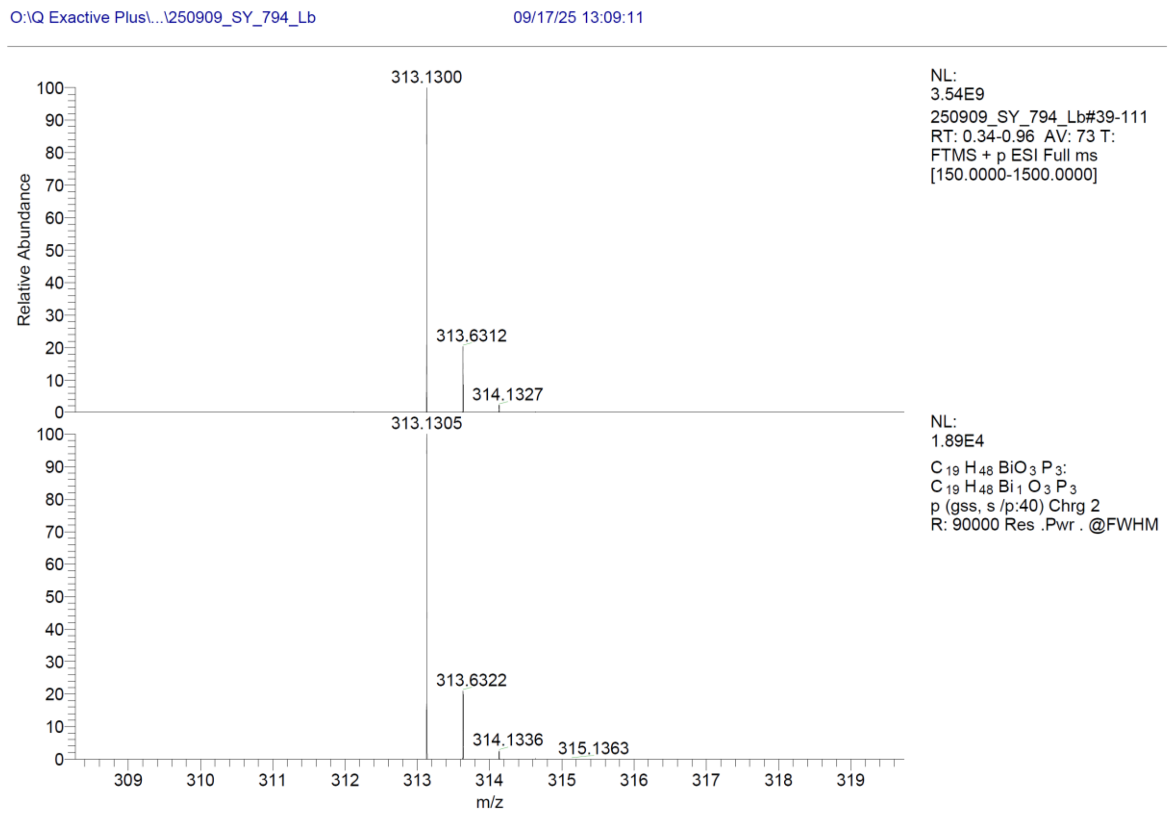

Figure S30: Top: HRMS-ESI spectrum of the 1:1 mixture of $OPEt_3$ and **1** in MeCN at m/z = 313; bottom: simulation of the signal corresponding to $[BiMe(OPEt_3)_3]^{2+}$.

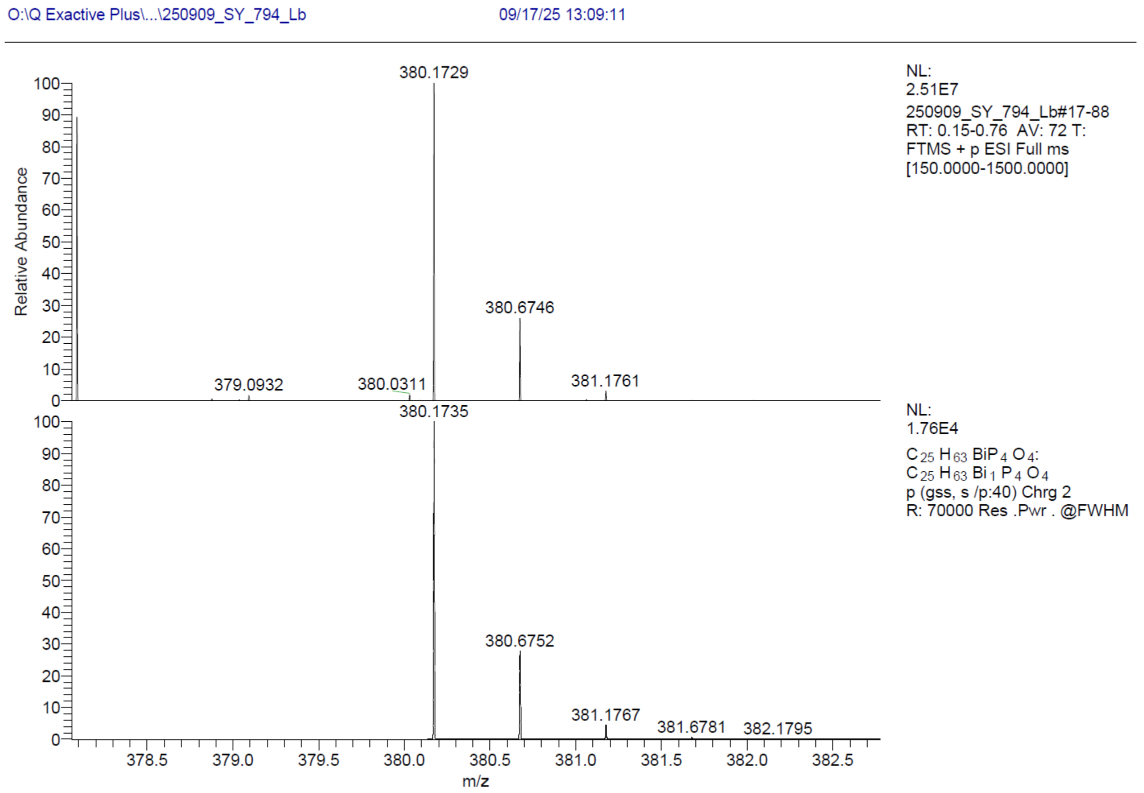

Figure S31: Top: HRMS-ESI spectrum of the 1:1 mixture of $OPEt_3$ and **1** in MeCN at m/z = 380; bottom: simulation of the signal corresponding to $[BiMe(OPEt_3)_4]^{2+}$.

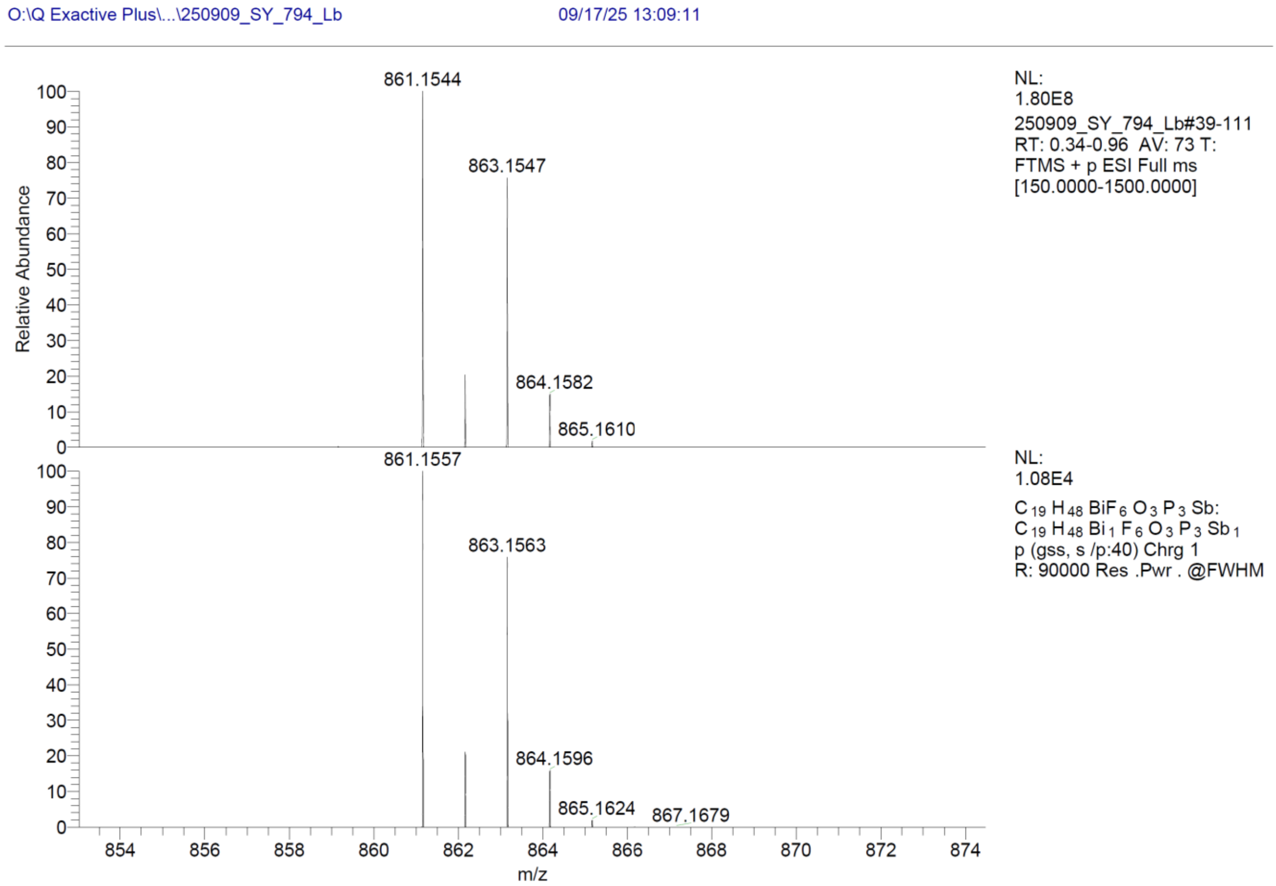

Figure S32: Top: HRMS-ESI spectrum of the 1:1 mixture of $OPEt_3$ and **1** in MeCN at m/z = 861; bottom: simulation of the signal corresponding to $[BiMe(OPEt_3)_3(SbF_6)]^+$.

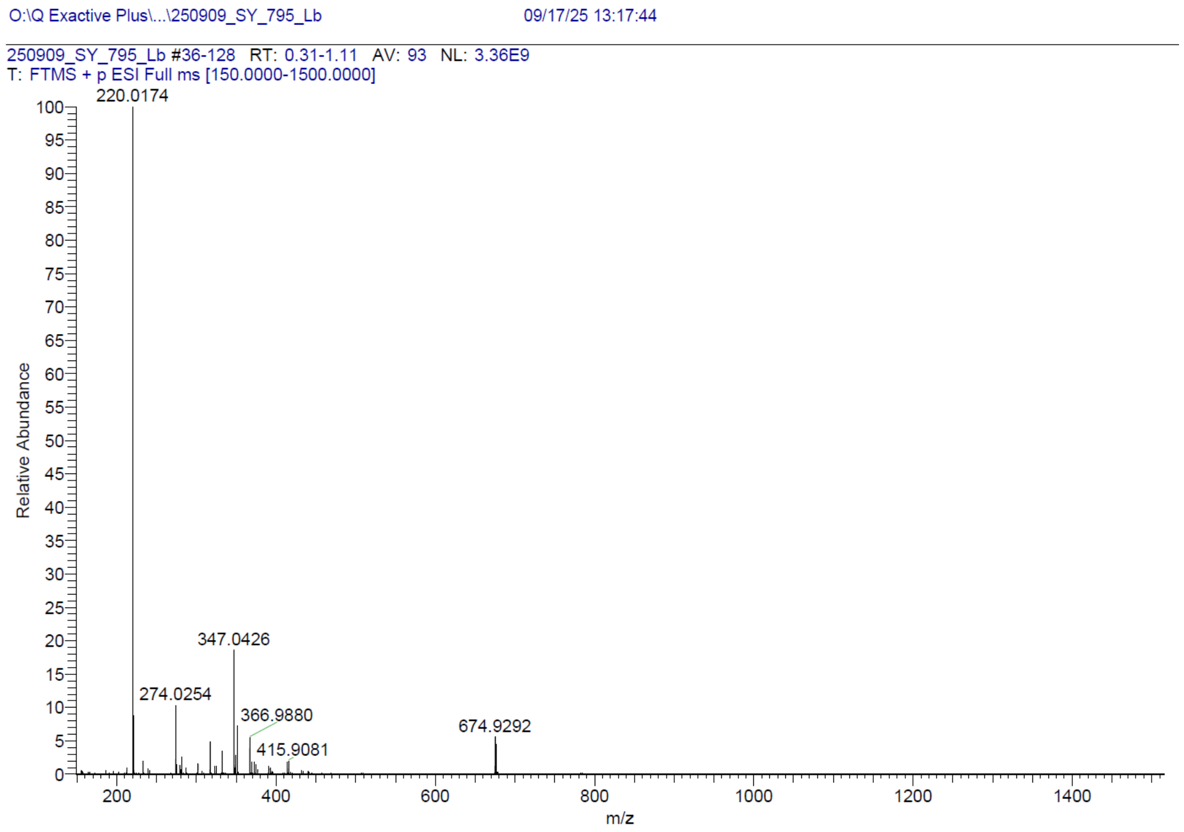

Figure S33: HRMS-ESI spectrum of the 1:1 mixture of $SPMe_3$ and **1** in MeCN.

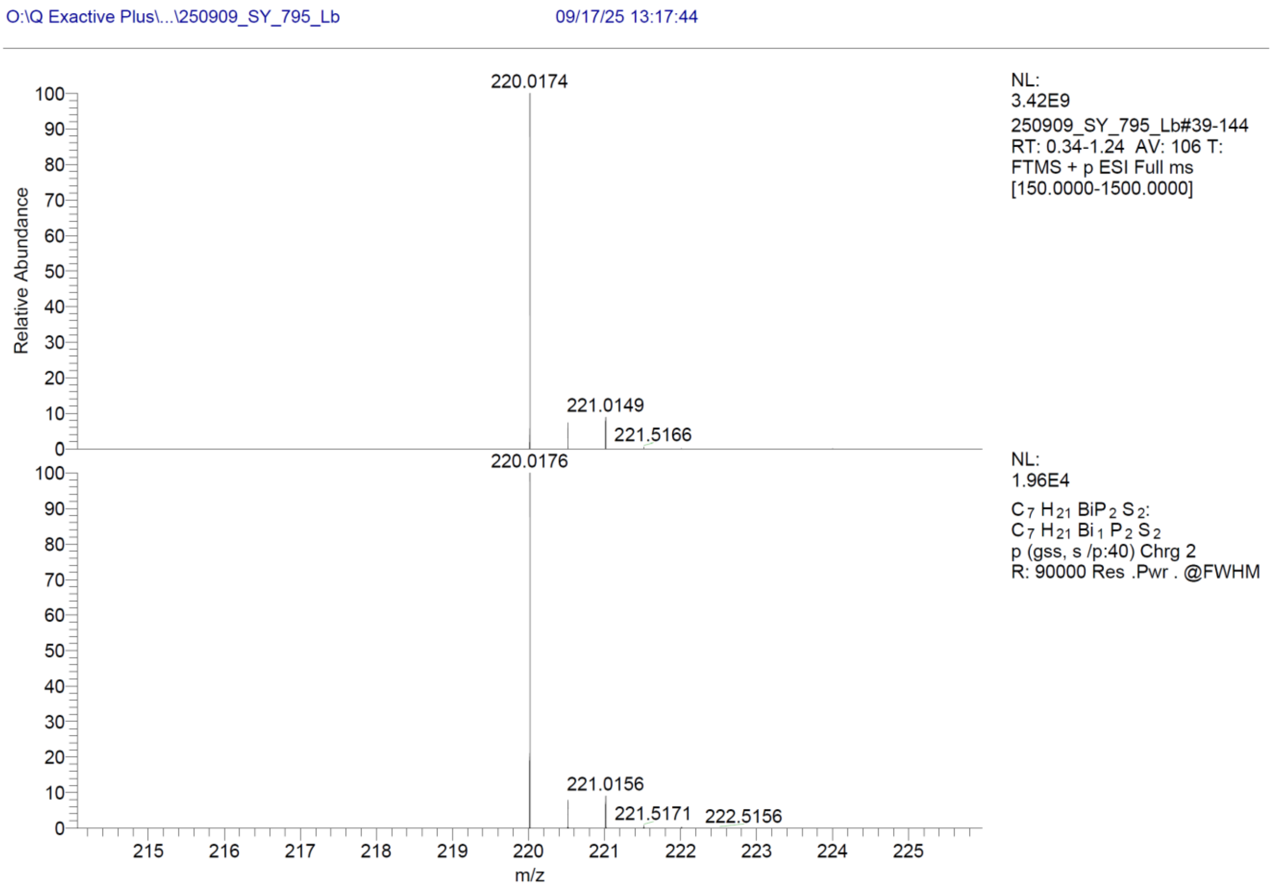

Figure S34: Top: HRMS-ESI spectrum of the 1:1 mixture of $SPMe_3$ and **1** in MeCN at m/z = 220; bottom: simulation of the signal corresponding to $[BiMe(SPMe_3)_2]^{2+}$.

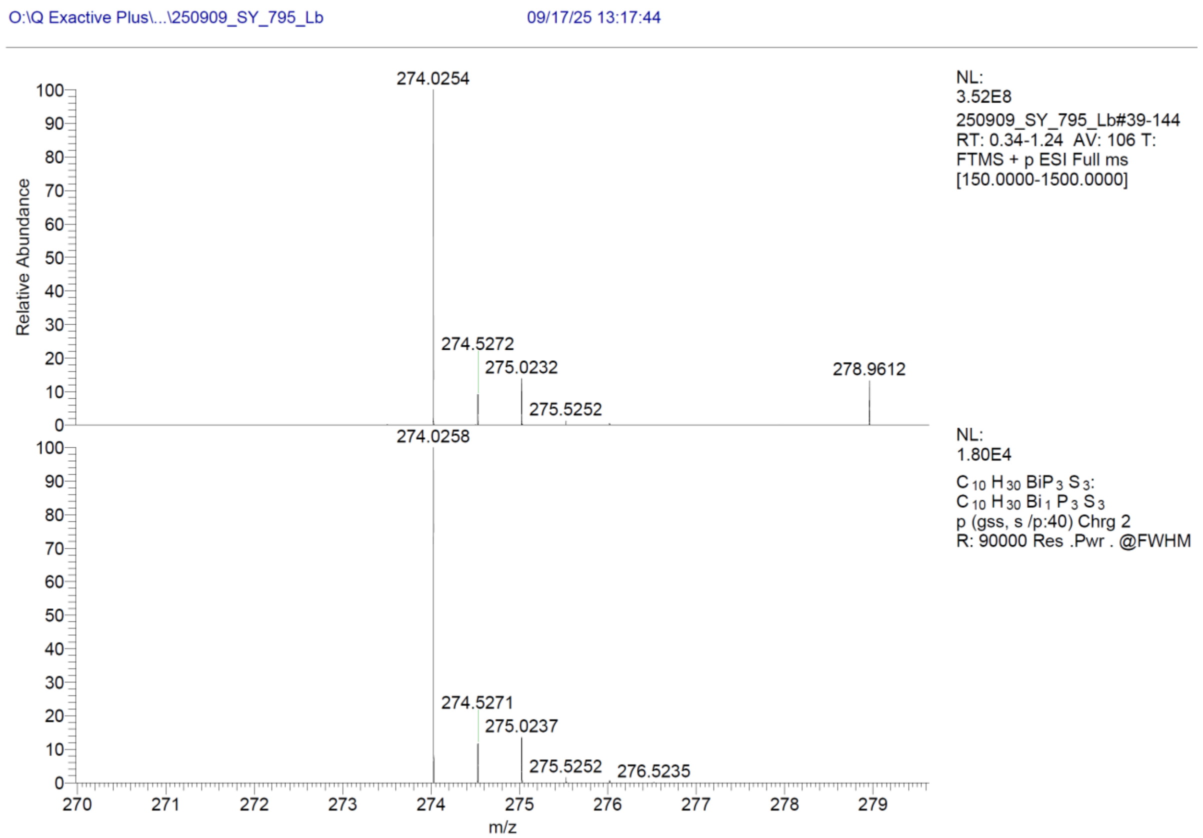

Figure S35: Top: HRMS-ESI spectrum of the 1:1 mixture of $SPMe_3$ and **1** in MeCN at m/z = 274; bottom: simulation of the signal corresponding to $[BiMe(SPMe_3)_3]^{2+}$.

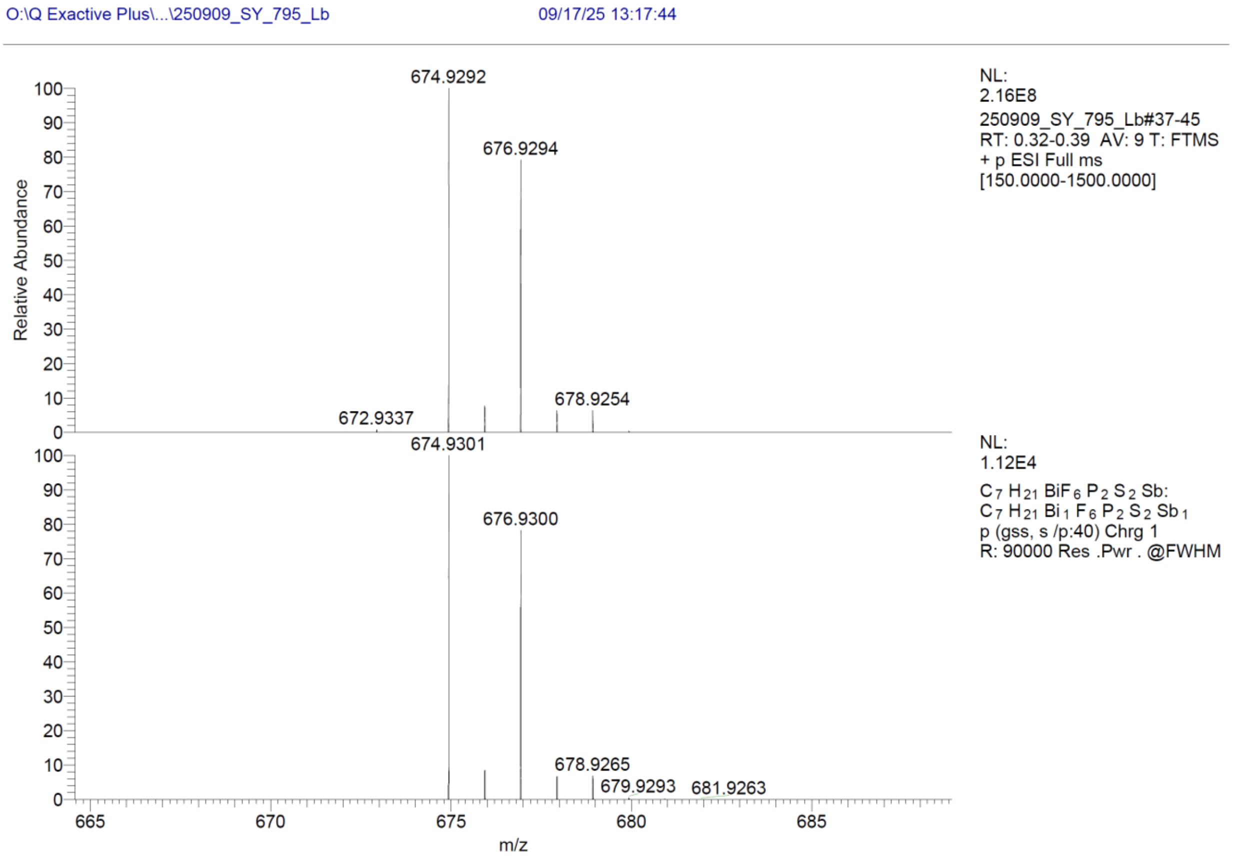

Figure S36: Top: HRMS-ESI spectrum of the 1:1 mixture of $SPMe_3$ and **1** in MeCN at m/z = 674; bottom: simulation of the signal corresponding to $[BiMe(SPMe_3)_2(SbF_6)]^+$.

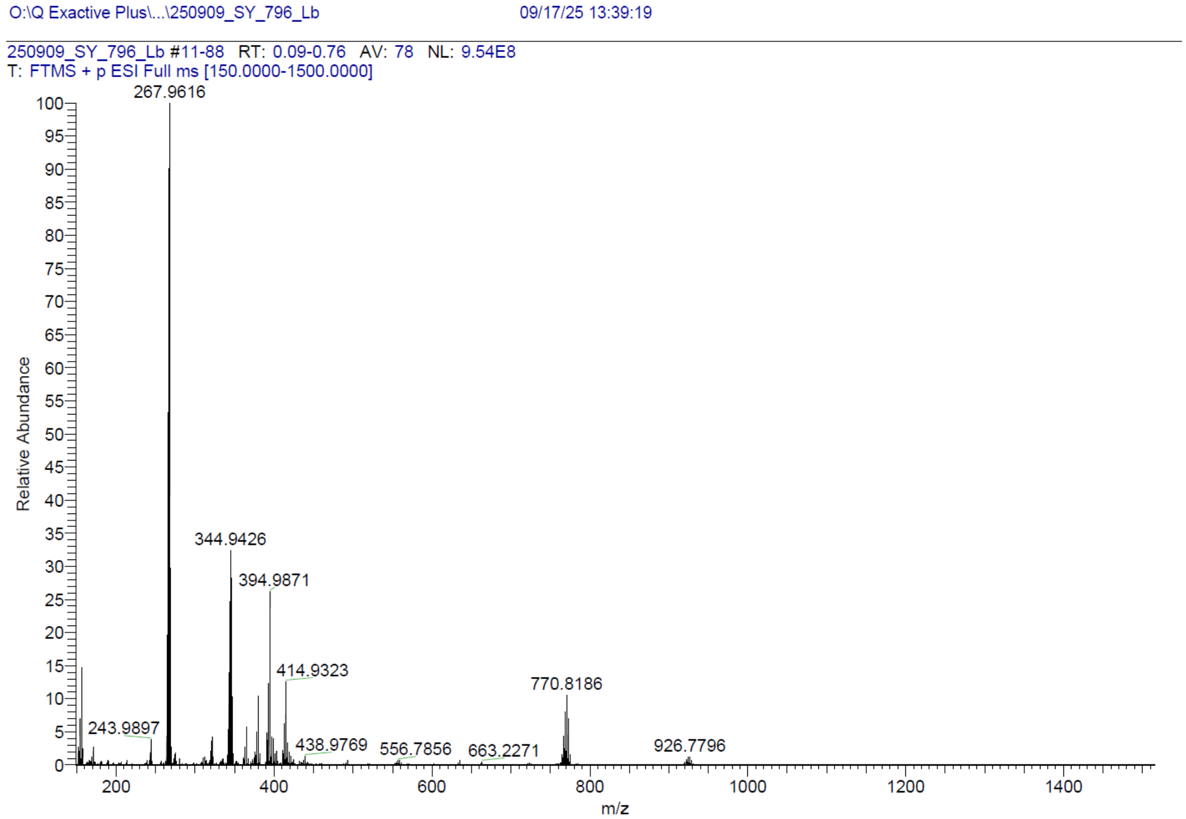

Figure S37: HRMS-ESI spectrum of the 1:1 mixture of $SePMe_3$ and **1** in MeCN.

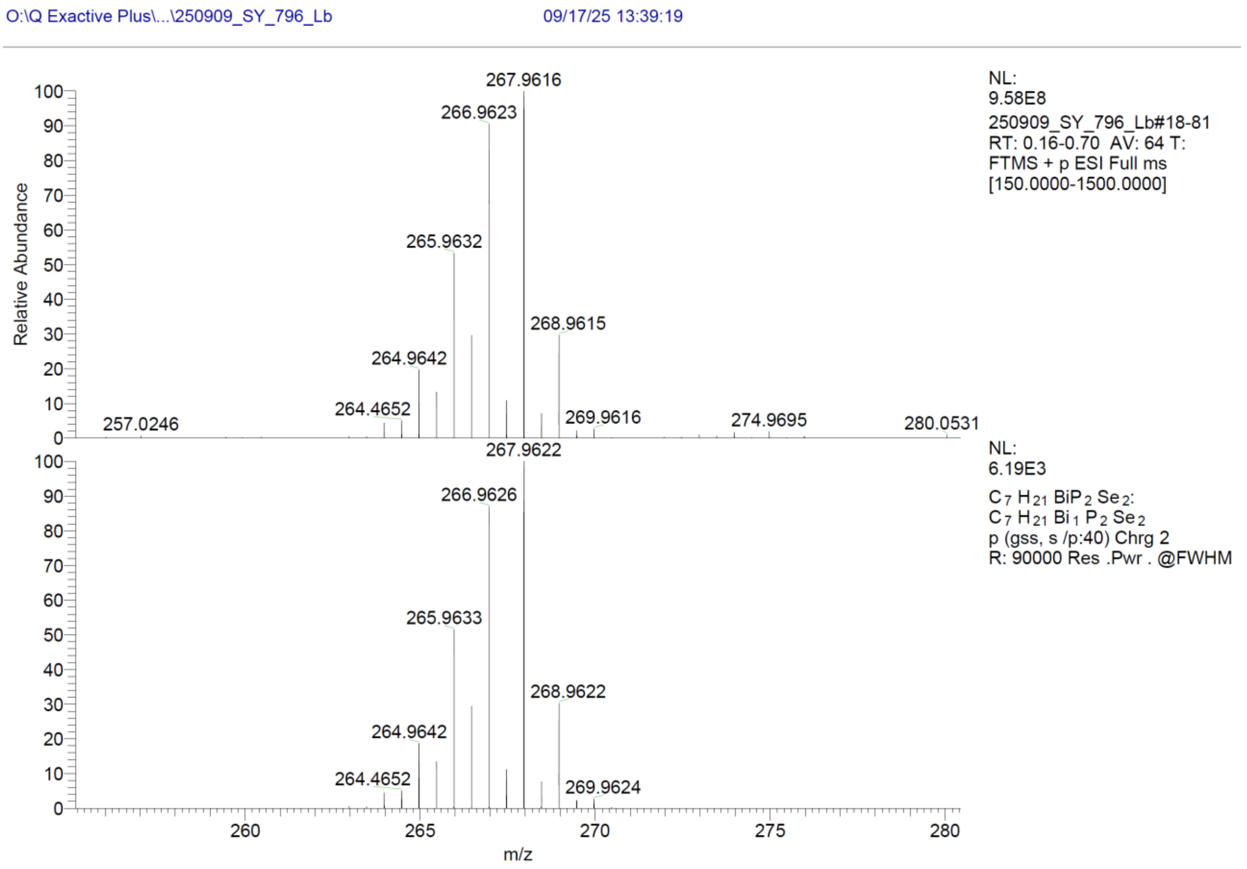

Figure S38: Top: HRMS-ESI spectrum of the 1:1 mixture of $SePMe_3$ and **1** in MeCN at m/z = 267; bottom: simulation of the signal corresponding to $[BiMe(SePMe_3)_2]^{2+}$.

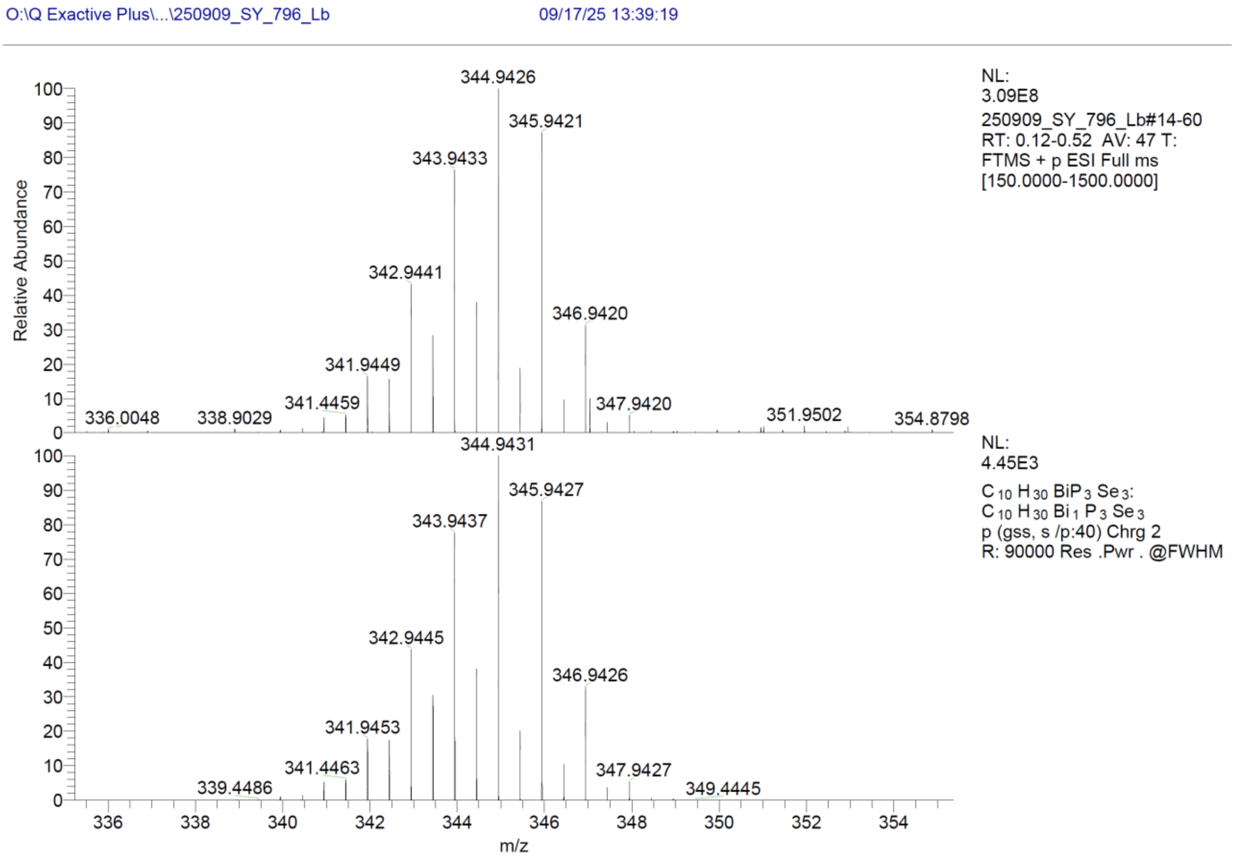

Figure S39: Top: HRMS-ESI spectrum of the 1:1 mixture of $SePMe_3$ and **1** in MeCN at m/z = 344; bottom: simulation of the signal corresponding to $[BiMe(SePMe_3)_3]^{2+}$.

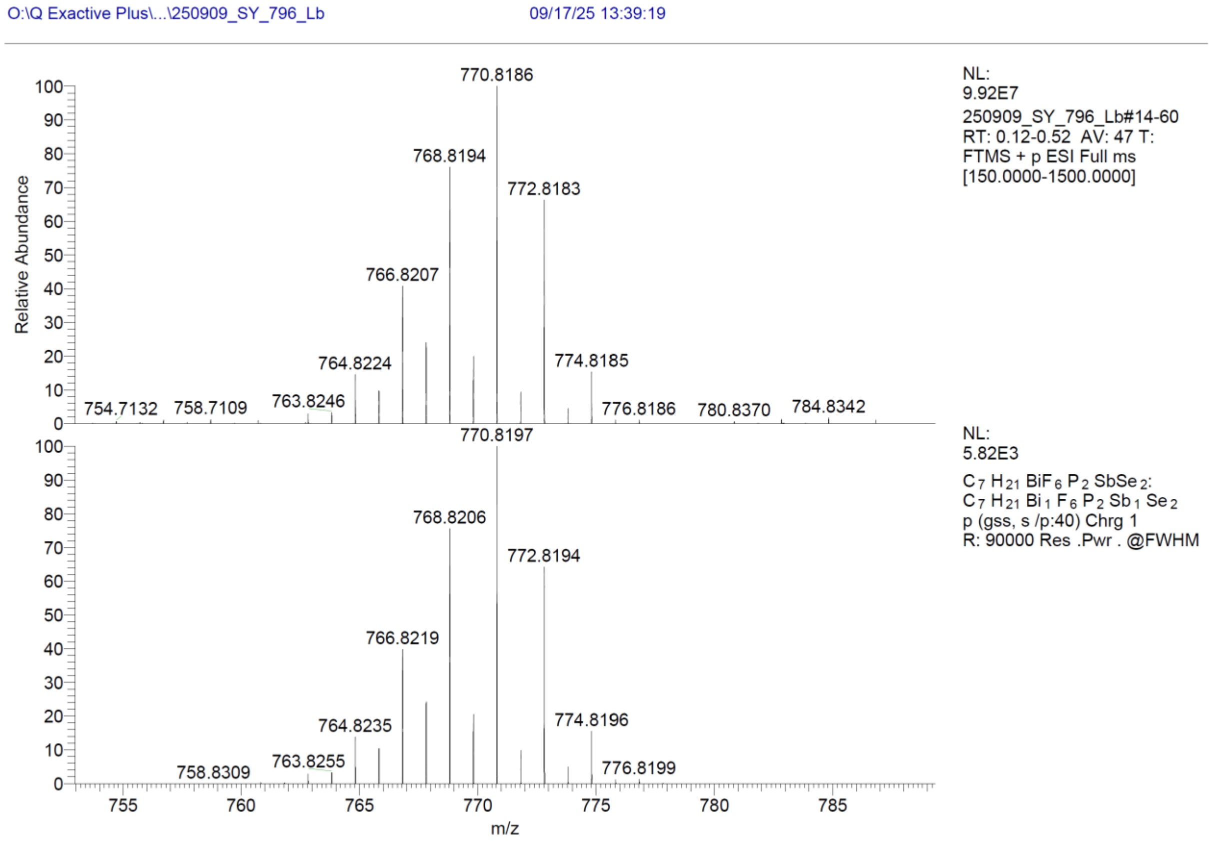

Figure S 40: Top: HRMS-ESI spectrum of the 1:1 mixture of $SePMe_3$ and **1** in MeCN at m/z = 770; bottom: simulation of the signal corresponding to $[BiMe(SePMe_3)_2(SbF_6)]^+$.

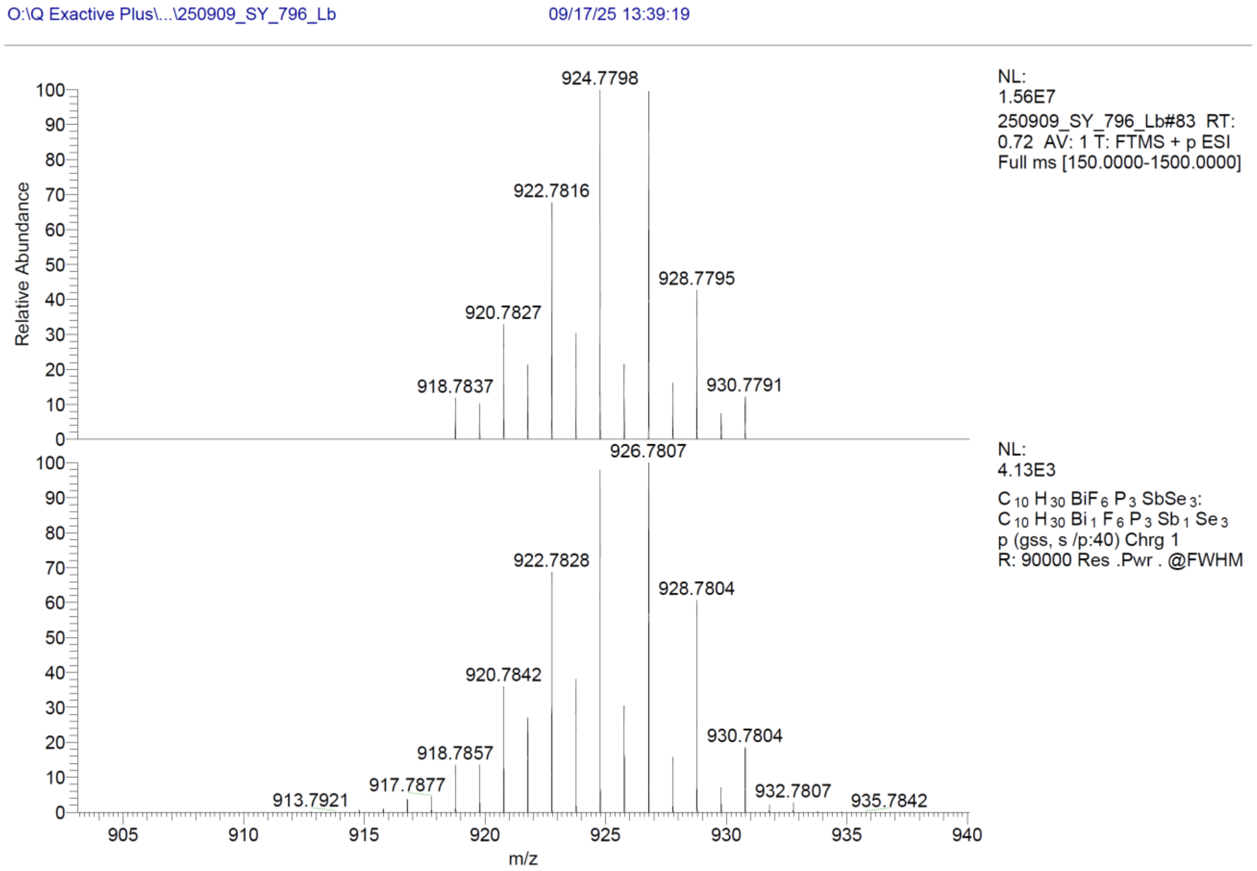

Figure S41: Top: HRMS-ESI spectrum of the 1:1 mixture of $SePMe_3$ and **1** in MeCN at m/z = 924; bottom: simulation of the signal corresponding to $[BiMe(SePMe_3)_3(SbF_6)]^+$.

10. Single-crystal X-ray diffraction

Compound	**1**	**2**
Empirical formula	$C_{21}H_{43}BiF_{12}O_5Sb_2$	$C_{41}H_{43}BiF_{12}N_8Sb_2$
Formula weight	1056.03	1328.31
Crystal color, habit	colorless, block	colorless, block
Temperature/K	100	100
Crystal system	monoclinic	triclinic
Space group	$P2_1/c$	$P\bar{1}$
a/Å	10.3514(19)	10.079(2)
b/Å	17.160(4)	21.610(6)
c/Å	18.126(5)	23.671(6)
α/°	90	69.551(11)
β/°	91.789(9)	79.459(11)
γ/°	90	85.220(14)
Volume/Å^3	3218.1(12)	4748(2)
Z	4	4
ρ_{calc}g/cm^3	2.180	1.858
μ/mm^{-1}	7.220	4.912
F(000)	2008.0	2552.0
Crystal size/mm	0.139x0.135x0.097	0.322x0.196x0.137
Diffractometer	Bruker D8 Quest	Bruker D8 Quest
Radiation	MoKα (λ = 0.71073)	MoKα (λ = 0.71073)
2Θ range for data collection/°	3.936 to 52.062	3.814 to 55.9
Reflections collected	8417	133504
Independent reflections	8417 [R_{int} = 0.0575]	22687 [R_{int} = 0.0434]
Data/restraint/parameters	8417/0/336	22687/0/1155
Goodness-of-fit on F^2	1.093	1.030
Final R indexes [all data]	R_1 = 0.0411, wR_2 = 0.0655	R_1 = 0.0322, wR_2 = 0.0538
Final R indexes [I≥2σ(I)]	R_1 = 0.0369, wR_2 = 0.0643	R_1 = 0.0254, wR_2 = 0.0518
Largest diff. peak/hole/ e Å^{-3}	1.58/–1.26	1.10/–1.17
CCDC	2474536	2474537

Compound	**3**	**4**
Empirical formula	$C_{25}H_{63}BiF_{12}O_4P_4Sb_2$	$C_7H_{21}BiF_{12}P_2S_2Sb_2$
Formula weight	1232.11	911.78
Crystal color, habit	colorless, block	colorless, plate
Temperature/K	100	100
Crystal system	monoclinic	monoclinic
Space group	$P2_1/n$	$P2_1/n$
a/Å	8.3412(17)	12.3866(4)
b/Å	12.272(2)	10.2899(4)
c/Å	21.813(5)	19.3216(5)
α/°	90	90
β/°	95.752(12)	105.338(2)
γ/°	90	90
Volume/Å^3	2221.5(7)	2374.96(14)
Z	2	4
ρ_{calc}g/cm^3	1.842	2.550
μ/mm^{-1}	5.380	35.979
F(000)	1196.0	1672.0
Crystal size/mm	0.169x0.169x0.123	0.35x0.33x0.03
Diffractometer	Bruker D8 Quest	STOE StadiVari
Radiation	MoKα (λ = 0.71073)	CuKα (λ = 1.54186)
2Θ range for data collection/°	3.812 to 57.518	7.662 to 130.178
Reflections collected	55906	24894
Independent reflections	5766 [R_{int} = 0.0339]	4038 [R_{int} = 0.0637]
Data/restraint/parameters	5766/30/361	4038/0/242
Goodness-of-fit on F^2	1.182	1.039
Final R indexes [all data]	R_1 = 0.0418, wR_2 = 0.0734	R_1 = 0.0992, wR_2 = 0.2450
Final R indexes [I≥2σ(I)]	R_1 = 0.0345, wR_2 = 0.0710	R_1 = 0.0843, wR_2 = 0.2321
Largest diff. peak/hole/ e Å^{-3}	0.84/–0.77	3.99/–1.81
CCDC	2474538	2474539

Compound	**4-MeCN**	**5**
Empirical formula	$C_{11}H_{27}BiF_{12}N_2P_2S_2Sb_2$	$C_{15}H_{42}BiF_{12}NP_4Sb_2Se_4$
Formula weight	993.88	1356.69
Crystal color, habit	colorless, needle	colorless, plate
Temperature/K	100	100
Crystal system	Monoclinic	Orthorhombic
Space group	$P2_1/m$	Pbcn
a/Å	15.0772(6)	12.6071(5)
b/Å	11.9439(5)	24.0275(10)
c/Å	16.8397(6)	12.8660(6)
α/°	90	90
β/°	110.2250(10)	90
γ/°	90	90
Volume/$Å^3$	2845.52(19)	3897.3(3)
Z	4	4
$\rho_{calc}g/cm^3$	2.320	2.312
μ/mm^{-1}	8.396	9.848
F(000)	1848.0	2512.0
Crystal size/mm	0.114x0.063x0.057	0.087x0.075x0.05
Diffractometer	Bruker D8 Quest	Bruker D8 Quest
Radiation	MoKα (λ = 0.71073)	MoKα (λ = 0.71073)
Θ range for data collection/°	4.274 to 50.048	4.638 to 50.054
Reflections collected	55787	94127
Independent reflections	5276 [R_{int} = 0.0449]	3449 [R_{int} = 0.0351]
Data/restraint/parameters	5276/0/329	3449/0/198
Goodness-of-fit on F^2	1.246	1.071
Final R indexes [all data]	R_1 = 0.0330, wR_2 = 0.0589	R_1 = 0.0222, wR_2 = 0.0482
Final R indexes [I≥2σ(I)]	R_1 = 0.0309, wR_2 = 0.0583	R_1 = 0.0209, wR_2 = 0.0476
Largest diff. peak/hole/ e $Å^{-3}$	1.09/–0.93	1.08/–0.98
CCDC	2474540	2474541

Compound	**6**	**7**
Empirical formula	$C_{58}H_{50}Bi_2F_{19}N_{12}Sb_3$	$C_{46}H_{44}Bi_2F_{19}N_{10}Sb_3$
Formula weight	2059.31	1881.12
Crystal color, habit	colorless, block	colorless, plate
Temperature/K	100	100
Crystal system	triclinic	triclinic
Space group	$P\bar{1}$	$P\bar{1}$
a/Å	12.7454(18)	13.7375(9)
b/Å	13.451(3)	14.3724(9)
c/Å	21.164(4)	15.4651(10)
α/°	89.444(13)	70.373(2)
β/°	84.381(10)	88.943(2)
γ/°	74.450(9)	83.612(2)
Volume/Å^3	3470.0(10)	2857.6(3)
Z	2	2
ρ_{calc}g/cm^3	1.971	2.186
μ/mm^{-1}	6.304	7.642
F(000)	1944.0	1760.0
Crystal size/mm	0.324x0.185x0.096	0.125x0.1x0.033
Diffractometer	Bruker D8 Quest	Bruker D8 Quest
Radiation	MoKα (λ = 0.71073)	MoKα (λ = 0.71073)
2Θ range for data collection/°	3.72 to 52.744	3.936 to 52.062
Reflections collected	103005	11537
Independent reflections	14156 [R_{int} = 0.0398]	11537 [R_{int} = 0.0477]
Data/restraint/parameters	14156/18/915	11537/36/784
Goodness-of-fit on F^2	1.051	1.052
Final R indexes [all data]	R_1 = 0.0360, wR_2 = 0.0794	R_1 = 0.0501, wR_2 = 0.0709
Final R indexes [I≥2σ(I)]	R_1 = 0.0302, wR_2 = 0.0751	R_1 = 0.0370, wR_2 = 0.0665
Largest diff. peak/hole/ e Å^{-3}	2.12/–1.51	1.36/–0.96
CCDC	2474542	2474543

11. Cartesian coordinates (Å) of compounds obtained from DFT geometry optimization

Coordinates of structures optimized with the BP86 functional are not included, as they are highly similar to those obtained by optimization with the B3LYP functional. The latter are presented here.

[BiMe]$^{2+}$

Bi	0.222927695	0.000000536	0.000008322
C	-1.934445863	0.000007585	0.000090964
H	-2.299434498	0.919628491	0.495187666
H	-2.299355016	-0.889166366	0.548040603
H	-2.297533995	-0.030552089	-1.044464770

[BiMe(thf)]$^{2+}$

Bi	1.005967464	-0.249033901	0.009342753
O	-1.121202541	-0.015127862	0.017841021
C	1.422542615	1.916598772	-0.093705501
H	2.496362001	2.043231941	-0.246942323
H	1.120921638	2.376637156	0.854075104
H	0.856472515	2.359662960	-0.917591371
C	-2.113329310	-1.218221173	0.087391149
H	-2.081427817	-1.541679089	1.125827672
H	-1.719195190	-1.973424842	-0.588497135
C	-1.952465771	1.290904880	-0.052836214
H	-1.834572330	1.640022140	-1.076986554
H	-1.503536204	1.974405873	0.662514257
C	-3.421544282	-0.591878980	-0.315269583
H	-4.254485549	-1.161262973	0.098652959
H	-3.528172095	-0.568637663	-1.400152544
C	-3.348677231	0.825030724	0.274222425
H	-3.514339677	0.815703964	1.351812176
H	-4.082862530	1.491571867	-0.179702558

[BiMe(thf)$_2$]$^{2+}$

Bi	0.004957062	-1.013701770	-0.282843762
O	-1.645783901	0.435731270	-0.029841802
O	1.615106639	0.476245231	0.001255273
C	-0.003475678	-1.576407785	1.872443424
H	0.911323092	-2.136626392	2.070983139
H	-0.868276390	-2.218019880	2.047015633
H	-0.051693456	-0.690453345	2.501613185
C	-2.311638574	1.110670892	-1.213723441
H	-2.880944429	0.338647799	-1.728953621
H	-1.516075604	1.497883073	-1.846773490
C	2.206756337	0.968343967	1.303640922
H	2.688131084	0.109028131	1.766471637
H	1.380216410	1.326501189	1.911892868
C	3.660197739	1.566347580	-0.509197905
H	4.107455460	2.370444788	-1.092063126
H	4.391261823	0.762142643	-0.418829793
C	-2.348602637	0.847335878	1.244558203
H	-1.672918401	1.531220006	1.755866713
H	-2.508558346	-0.054027201	1.829731332
C	-3.184935132	2.165224421	-0.572498688
H	-4.034942587	2.407020789	-1.209107592
H	-2.619854852	3.080910992	-0.394621677
C	2.391623260	1.065608798	-1.158978981
H	1.773264659	1.864619046	-1.565328052
H	2.532968583	0.271243929	-1.888990574
C	-3.612843259	1.515002459	0.750299963
H	-4.399158905	0.777182464	0.587599365
H	-3.974734261	2.245513411	1.472852983
C	3.183877682	2.041095235	0.870631795

H	4.003037226	2.129368270	1.583154657
H	2.687722406	3.009506444	0.799159095

[BiMe(thf)$_3$]$^{2+}$

Bi	-0.014142669	-0.768801191	-0.137176390
O	2.347810596	-0.583454377	-0.035521204
O	-2.364762953	-0.433398004	-0.076775585
O	0.070942337	1.464226426	0.034538035
C	-0.086963369	-1.011974372	2.081484196
H	-0.929294967	-0.451312057	2.478213804
H	0.846180044	-0.654541482	2.508680549
H	-0.210488209	-2.073199974	2.300053990
C	3.190604816	-0.054538422	1.063688255
H	3.212990548	-0.808776367	1.850278967
H	2.717218957	0.855111847	1.429996897
C	-3.338528796	-0.829538854	0.975969781
H	-3.333337435	-0.043530475	1.732108163
H	-2.998041856	-1.766555659	1.411224318
C	-4.537781320	0.153772192	-0.835685644
H	-5.236772421	0.010981556	-1.657837241
H	-4.705984023	1.146657533	-0.415512247
C	-3.099396524	0.001281375	-1.293312858
H	-2.983331829	-0.779858540	-2.045597866
H	-2.639416651	0.926115368	-1.639166164
C	-0.692439289	2.299889003	1.010734488
H	-0.184366324	2.203469654	1.969139369
H	-1.697577384	1.890012341	1.067018405
C	0.749618177	3.706041549	-0.282233735
H	0.828339701	4.498242243	-1.024960249
H	1.562800971	3.828817201	0.434438937
C	3.215683081	-1.001287288	-1.172128067
H	3.074851632	-0.273366174	-1.972388972
H	2.885602999	-1.985507175	-1.499606698
C	-4.664533664	-0.924625808	0.246595779
H	-5.503001632	-0.750924848	0.918994405
H	-4.785984060	-1.911783619	-0.201347019
C	4.552031936	0.155846299	0.428915589
H	5.350212338	0.102786450	1.167354449
H	4.605444230	1.130720709	-0.059007144
C	0.808874651	2.336736562	-0.927661757
H	0.277312156	2.291778870	-1.878289605
H	1.809202202	1.922808916	-1.028994390
C	4.621808459	-0.975627965	-0.603365804
H	4.857949674	-1.925110128	-0.121415311
H	5.363422878	-0.795122404	-1.379525903
C	-0.609753217	3.697632817	0.429567418
H	-0.677729633	4.454279113	1.209567648
H	-1.419629770	3.867671019	-0.281116526

[BiMe(thf)$_4$]$^{2+}$

Bi	-0.130080590	-0.105567919	0.112695890
O	1.171579014	-2.120015878	-0.059478501
O	-0.922542157	2.166152154	0.046326666
O	2.037584154	0.944146633	-0.047909200
O	-2.328946031	-1.103645419	0.022162767
C	2.738116220	-3.676493678	0.795748605
H	2.832432158	-4.626041629	1.319455246
H	3.581242128	-3.045669396	1.083130746
C	-0.201652371	-0.135561874	-2.119158901
H	0.786040134	0.103786022	-2.504260628
H	-0.934470691	0.583244649	-2.474622886
H	-0.489537386	-1.138216801	-2.425801893

C	2.670459914	-3.839095522	-0.727166101
H	3.647993059	-3.991473735	-1.181320061
H	2.037401778	-4.685992897	-0.994819012
C	-1.086418784	2.928737089	1.301218872
H	-0.090187636	3.203181925	1.653191631
H	-1.567386519	2.276547624	2.029134903
C	2.360926778	2.175714180	-0.787981707
H	1.749493741	2.980911769	-0.377435631
H	2.101133997	2.012094088	-1.832145132
C	2.036592772	-2.535389844	-1.183655405
H	2.769821785	-1.742565695	-1.338555487
H	1.410777746	-2.633851214	-2.067414759
C	4.024833413	1.850721280	0.883512896
H	5.058473598	1.597915940	1.112329895
H	3.683830244	2.580110349	1.619920289
C	3.128517344	0.624138905	0.891830842
H	3.632682088	-0.264494036	0.510274053
H	2.687221015	0.405467746	1.864275541
C	-3.070509764	-1.422255568	1.260918585
H	-3.462206690	-0.485761097	1.661293160
H	-2.367944960	-1.862151937	1.967821267
C	1.424343903	-2.986843285	1.109157900
H	0.593545444	-3.689786806	1.182788136
H	1.451436847	-2.354688514	1.996514464
C	-4.498078759	-1.852186291	-0.591899342
H	-5.012725886	-2.591716053	-1.202897157
H	-5.121225224	-0.957891790	-0.544278354
C	-1.310204768	3.010146256	-1.102998669
H	-2.253277002	2.622388658	-1.489884532
H	-0.533773561	2.919059437	-1.859671964
C	3.842639819	2.390904654	-0.539882409
H	4.121252868	3.439013370	-0.634181689
H	4.436655108	1.816029673	-1.251881926
C	-1.913813226	4.138984582	0.910411764
H	-1.738833572	4.981197325	1.577395870
H	-2.977979244	3.899960515	0.936063176
C	-3.126709552	-1.518662556	-1.152247190
H	-2.634247187	-2.387912003	-1.589648064
H	-3.124300510	-0.695495076	-1.863351952
C	-1.451499533	4.407829728	-0.526087258
H	-0.490273396	4.924558818	-0.531585360
H	-2.160004363	5.007739031	-1.094515079
C	-4.174937508	-2.362814518	0.816832234
H	-5.031020030	-2.326338758	1.488163261
H	-3.812386089	-3.391363350	0.782618966

$[BiMe(thf)_5]^{2+}$ (PPY-6)

Bi	-0.000419214	0.009160418	-0.072068377
O	1.091487360	-2.217395212	0.032675111
O	-2.193656353	1.187820046	-0.064419576
O	2.468565606	0.357057763	-0.006715306
O	0.430841835	2.439441399	0.085899992
O	-1.784488291	-1.715686803	0.081741559
C	1.827699127	-4.273396243	-0.878400361
H	2.664546591	-4.746594297	-1.388678171
H	0.909571986	-4.753126762	-1.221814323
C	-0.027320854	0.029929969	2.161449308
H	-0.212864585	-0.978414088	2.519160931
H	-0.820905607	0.696338918	2.488597066
H	0.933061354	0.387006289	2.521596257
C	1.938781650	-4.343147747	0.648887536
H	1.629550114	-5.304297269	1.055399276

H	2.966180565	-4.158700726	0.966959290
C	3.160612044	0.946233989	-1.154217978
H	3.089262483	2.032249873	-1.068306713
H	2.650634034	0.624379707	-2.062732414
C	1.203867462	3.139669341	1.115587060
H	2.224187393	2.754922937	1.085159093
H	0.759675884	2.908305185	2.082097719
C	1.028811913	-3.212717265	1.105514362
H	-0.011676621	-3.525433301	1.208488756
H	1.355440977	-2.734170653	2.027198892
C	1.023006950	4.555850553	-0.794018413
H	0.604359170	5.462671817	-1.226476417
H	2.006157614	4.393236173	-1.239209078
C	0.127711965	3.349509833	-1.020962214
H	-0.931920612	3.604051265	-0.964181019
H	0.320827389	2.823962724	-1.956463774
C	-2.891912787	-1.770914323	1.040014585
H	-3.505766302	-0.881094587	0.895194748
H	-2.468333329	-1.756149558	2.042390798
C	-2.708239587	2.147449522	0.914766241
H	-1.883444056	2.791639911	1.212928591
H	-3.063294083	1.585010577	1.780746929
C	1.775673944	-2.776566138	-1.133821937
H	2.771386652	-2.331421612	-1.186893176
H	1.211267318	-2.499174382	-2.024391416
C	-3.436628082	-3.158019854	-0.815905459
H	-3.587780853	-4.166230659	-1.196998080
H	-4.122424196	-2.492494160	-1.343479862
C	3.443677485	-0.187917470	0.940245967
H	3.384458741	-1.275882083	0.892781906
H	3.165538928	0.143605154	1.939225106
C	1.121905638	4.609400777	0.734810331
H	1.985915801	5.170065490	1.086801141
H	0.227393199	5.066254636	1.161281632
C	-3.840040066	2.868816936	0.200719027
H	-4.588577075	3.242424556	0.897044984
H	-3.457044852	3.716592550	-0.370201401
C	4.594103236	0.454265115	-1.045942778
H	5.297995826	1.136868611	-1.518372720
H	4.701013144	-0.522417587	-1.521389819
C	-1.999965949	-2.692466268	-0.987987746
H	-1.283476882	-3.503416728	-0.848917144
H	-1.805378066	-2.202729562	-1.942803709
C	4.788704460	0.343779637	0.470345613
H	4.985458890	1.325354356	0.904506849
H	5.607733166	-0.316979020	0.748544270
C	-4.382273044	1.787105850	-0.740358072
H	-5.028051229	1.094992556	-0.196895670
H	-4.949996172	2.192627204	-1.575735412
C	-3.113904527	1.085923315	-1.199040106
H	-2.654799855	1.585584248	-2.055072626
H	-3.247745867	0.029134117	-1.425823377
C	-3.637837068	-3.050266322	0.699837393
H	-4.686230863	-3.000187294	0.988284937
H	-3.187716122	-3.903716060	1.209533966

[BiMe(thf)$_5$]$^{2+}$ (OC-6)

Bi	-0.183007208	0.489436580	0.194852823
O	-2.455969610	1.216737656	-0.430765349
O	1.205382571	-1.627853593	-1.655091341
O	-1.335171446	-1.408144004	1.120938132
O	1.637462436	-0.483548517	1.381655169

O	1.425026499	2.222392228	-0.353532565
C	-4.274533715	1.572300322	-1.911435288
H	-5.077520809	1.060448278	-2.438770381
H	-3.951310563	2.417195174	-2.521402451
C	-0.556354724	1.647980902	2.082133068
H	-0.897293327	2.645397513	1.810632288
H	0.377891514	1.714668655	2.634032024
H	-1.314351570	1.157181868	2.686888073
C	-4.680215763	2.039796332	-0.509179295
H	-5.260696461	2.960585669	-0.519636431
H	-5.270498766	1.273730183	-0.003138174
C	-1.285616780	-2.709232889	0.431425964
H	-0.583663344	-3.341751766	0.977915926
H	-0.905792978	-2.537076883	-0.573438280
C	1.551726593	-1.225915865	2.649861895
H	1.017478731	-2.154673590	2.449985828
H	0.981732141	-0.622481406	3.353894733
C	-3.340168986	2.225440321	0.181613350
H	-2.906172378	3.207068404	-0.013540792
H	-3.361987732	2.042235321	1.253830547
C	3.718254743	-1.551502497	1.705094209
H	4.789343722	-1.373770539	1.783450072
H	3.567542628	-2.532861568	1.252452278
C	3.023842835	-0.478985843	0.887559445
H	3.436363181	0.513484731	1.075551520
H	2.988237953	-0.671931967	-0.181561052
C	1.977722909	3.289186823	0.501432975
H	2.473989292	2.807726878	1.345562264
H	1.147263838	3.891668445	0.862669863
C	1.873923035	-2.896348366	-1.443666593
H	1.596952917	-3.261596230	-0.453813162
H	2.957019607	-2.739382779	-1.468540120
C	-3.105334342	0.647751265	-1.621247381
H	-3.432861790	-0.363448995	-1.368898087
H	-2.365772407	0.602696045	-2.420415667
C	3.435529166	2.959268250	-1.361382648
H	3.857864343	3.368619450	-2.277319895
H	4.191818088	2.328087409	-0.891086883
C	-2.548186567	-1.337676957	1.957833084
H	-3.195085889	-0.571317591	1.528965759
H	-2.242635131	-1.046047370	2.960505589
C	2.997373950	-1.459417326	3.055548754
H	3.106939131	-2.359032350	3.658524745
H	3.373771239	-0.615228264	3.635182943
C	1.421455040	-3.792599977	-2.585803719
H	2.125921998	-4.596614516	-2.791486746
H	0.451502753	-4.242023705	-2.359877272
C	-2.704817620	-3.234097233	0.509113439
H	-2.745620842	-4.318503560	0.422892961
H	-3.315928996	-2.806932932	-0.288715671
C	2.168829648	2.167620441	-1.625652406
H	1.546694737	2.630069243	-2.393562261
H	2.333501109	1.118073304	-1.867529166
C	-3.163442232	-2.727466834	1.881423493
H	-2.769155529	-3.364333516	2.674200371
H	-4.246580805	-2.694330030	1.984254539
C	1.297887823	-2.788191522	-3.736616876
H	2.279865936	-2.577884508	-4.163345621
H	0.649009463	-3.130839929	-4.540590833
C	0.746543361	-1.552095927	-3.031384536
H	-0.349096038	-1.546353878	-3.027382713
H	1.098174488	-0.615057488	-3.466570111

C	2.949940343	4.043059240	-0.391513899
H	3.758068542	4.493421259	0.182142930
H	2.434680349	4.837975334	-0.932272404

$[BiMe(thf)_6]^{2+}$

Bi	0.129614648	0.033521104	-0.465373142
O	2.405157487	-0.998653276	-0.285313187
O	-1.081182469	2.194372541	-0.563345033
O	-0.157736856	-2.434511314	-0.609044235
O	-2.204375510	-0.433366028	-1.122103994
O	1.823660150	1.879958370	-0.266611269
C	4.368413204	-1.674556104	0.857569769
H	4.796869819	-2.508447986	1.410492000
H	4.790476257	-0.752186157	1.260498932
C	0.491593715	0.145109387	-2.677289152
H	1.507460678	0.488003144	-2.851528181
H	-0.216419942	0.845482049	-3.113619358
H	0.352426942	-0.841724234	-3.111827300
C	4.613099742	-1.765460241	-0.652752207
H	5.607622991	-1.433231784	-0.944829886
H	4.484999651	-2.791712080	-1.001213145
C	-0.985888452	-3.121336027	0.380696237
H	-2.003756070	-3.185784419	-0.011432519
H	-0.993183335	-2.517859726	1.287487083
C	-2.648196035	-1.190054682	-2.293469527
H	-2.227846892	-2.194753586	-2.224339304
H	-2.255125587	-0.695630498	-3.179474028
C	3.522431257	-0.871300278	-1.220395430
H	3.816235186	0.179894053	-1.250996110
H	3.171422903	-1.176461515	-2.204436662
C	-4.411125364	-1.187107678	-0.692354425
H	-5.402723549	-0.828598511	-0.422173784
H	-4.294659395	-2.192524940	-0.283990468
C	-3.313022929	-0.270597848	-0.177581389
H	-3.609214606	0.779750228	-0.195822598
H	-2.945499360	-0.525031174	0.813399069
C	1.977486933	3.051586725	-1.128950707
H	1.052714119	3.628446114	-1.082518795
H	2.129169580	2.700426565	-2.147883140
C	-1.893788640	2.752272965	-1.644001394
H	-2.436985652	1.931305460	-2.107334277
H	-1.217696853	3.200372624	-2.374997824
C	2.850265569	-1.650459225	0.944440999
H	2.420750832	-2.654357971	0.967327957
H	2.466598723	-1.080140096	1.789767744
C	3.045390345	3.506614007	0.947639161
H	3.977383041	3.659269885	1.488697312
H	2.280981209	4.134921141	1.408915841
C	0.584262764	-3.414135684	-1.404909112
H	1.645213200	-3.249240894	-1.220058328
H	0.371444765	-3.229368769	-2.457326924
C	-4.165231207	-1.187247582	-2.205265285
H	-4.604857870	-2.045769759	-2.709790192
H	-4.572572734	-0.283082732	-2.660782241
C	-2.779974357	3.788775957	-0.971667967
H	-3.077129634	4.579303530	-1.658355809
H	-3.686337163	3.323325687	-0.580042729
C	-0.349830824	-4.490974010	0.518997678
H	-1.047516980	-5.234901615	0.899563567
H	0.504418500	-4.448256017	1.197772780
C	2.613152706	2.048734284	0.950636627
H	3.463666896	1.366942787	0.896300077

H	1.989699048	1.774279965	1.802720649
C	0.105756440	-4.777770087	-0.917184439
H	-0.734962338	-5.126631193	-1.518424447
H	0.891901064	-5.528305566	-0.974969070
C	-1.894208129	4.290497172	0.174631887
H	-1.162560410	5.011067645	-0.195261796
H	-2.459101975	4.765090672	0.974917422
C	-1.203033114	3.018198704	0.638894881
H	-1.794510294	2.463362190	1.369489077
H	-0.201556029	3.176892743	1.036705486
C	3.160619887	3.810154162	-0.550437675
H	3.114065127	4.874154896	-0.775364809
H	4.098291132	3.420810874	-0.950497020
O	-1.147419718	-0.296332986	2.584974631
C	-1.774201228	0.262428311	4.804398730
H	-2.534479464	0.090660873	5.564354496
H	-1.399977085	1.281460468	4.922885115
C	-0.622012068	-0.745337090	4.863235657
H	0.115384343	-0.514657167	5.630264633
H	-1.004106294	-1.750031994	5.053037836
C	-0.047634245	-0.652003543	3.453284571
H	0.718269711	0.129794433	3.385431252
H	0.384094065	-1.592876421	3.103599318
C	-2.301688280	0.048247348	3.392873993
H	-3.018811333	-0.778039033	3.355558487
H	-2.768635634	0.934924127	2.961371127

[BiMe(thf)$_5$][SbF$_6$]$_2$ (PPY-6)

Bi	0.605642864	0.517371722	0.480359571
O	2.152510007	2.461467275	1.499171810
O	-0.624317614	-1.393547211	1.309334218
O	0.899296681	2.402049137	-1.121830516
O	-1.003184923	0.078002883	-1.233394362
O	1.767138273	-0.229921383	2.703509308
C	4.304974144	3.238013964	2.048617214
H	5.091183075	3.940664575	1.775461559
H	4.775890364	2.325103950	2.417806760
C	-0.903280158	1.747906072	1.602251971
H	-0.866418221	1.440422435	2.646400298
H	-1.900405483	1.585821240	1.200952039
H	-0.618393660	2.793074373	1.520009767
C	3.321484092	3.800575567	3.080514215
H	3.689375651	3.744468681	4.104223600
H	3.100218781	4.846633726	2.859412652
C	1.743232675	2.280229433	-2.311653502
H	1.206296534	1.677732713	-3.045239217
H	2.649245827	1.752751790	-2.027709516
C	-1.783101344	1.035630674	-2.031877851
H	-1.083900572	1.779993317	-2.408343288
H	-2.516127343	1.494683614	-1.374469831
C	2.081006322	2.933889116	2.869171744
H	2.068392975	2.059811535	3.520648290
H	1.147037636	3.480535331	2.999824776
C	-1.438808328	-0.972145121	-3.293050938
H	-1.904782652	-1.838458555	-3.760489025
H	-0.549653403	-0.702704883	-3.866643172
C	-1.056153174	-1.251050435	-1.854487000
H	-1.822251740	-1.821567968	-1.332801843
H	-0.075065614	-1.705485853	-1.746200195
C	1.225052581	-0.912615565	3.856012350
H	0.206424007	-1.199812181	3.610734361
H	1.202472927	-0.210406628	4.694501326

C	-2.046588031	-1.332360467	1.697056192
H	-2.552998418	-0.680040714	1.000563098
H	-2.092449246	-0.906601634	2.700556885
C	3.384856878	2.914313352	0.884129699
H	3.169672252	3.806669175	0.286982151
H	3.748778629	2.124234606	0.234132287
C	3.301881293	-1.946066785	3.102898598
H	4.280935516	-2.183141426	3.516503938
H	3.140989168	-2.576762165	2.230156068
C	0.352772382	3.742002895	-1.014193561
H	0.848753840	4.241577778	-0.178759657
H	-0.711870164	3.660325085	-0.799644996
C	-2.401661054	0.213154053	-3.154778654
H	-2.486989552	0.797651347	-4.070363167
H	-3.393594753	-0.125254507	-2.866663010
C	-2.525053256	-2.770044383	1.652071257
H	-3.343574247	-2.934507745	2.348735185
H	-2.889480466	-3.005633449	0.652415259
C	1.960974735	3.711596746	-2.773435054
H	2.149010663	3.769021495	-3.844397835
H	2.814277394	4.153399095	-2.254941026
C	3.190658099	-0.489908023	2.685620953
H	3.676503425	0.180811657	3.404546024
H	3.562370524	-0.280990536	1.686919945
C	0.658880233	4.402043191	-2.351632277
H	-0.135611739	4.187716139	-3.068974727
H	0.751965083	5.483546484	-2.262581467
C	-1.255371871	-3.561388064	1.983446776
H	-1.061861107	-3.549264603	3.059282100
H	-1.298421546	-4.600191987	1.658471355
C	-0.180580642	-2.786330993	1.242117314
H	-0.111246328	-3.075806024	0.194300482
H	0.812487588	-2.838839992	1.677304746
C	2.163006126	-2.104825427	4.134207164
H	1.647544416	-3.057522157	4.021567992
H	2.542311118	-2.057564448	5.154712609
Sb	3.557156138	-1.570921831	-1.523246813
F	3.256406033	-3.209555405	-2.403296519
F	4.655363113	-0.901328955	-2.902772001
F	5.024017380	-2.241784680	-0.539430169
F	3.728748608	0.113736485	-0.583861886
F	2.007247883	-0.798749120	-2.354903233
F	2.381555504	-2.106339006	-0.091487673
Sb	-5.348049803	-0.041401753	-0.057854996
F	-3.880253192	1.135412146	0.381361323
F	-5.429515952	0.797917994	-1.762922440
F	-4.032567005	-1.248351657	-0.785256460
F	-5.078800767	-0.883281926	1.632978717
F	-6.564908127	1.195968820	0.686102249
F	-6.725121460	-1.252485495	-0.507058476

THF

O	0.00000351	-1.24917522	-0.00001403
C	-1.16597061	-0.42750984	0.13252350
H	-1.52826564	-0.47802248	1.16677916
H	-1.94644042	-0.81905460	-0.52347480
C	1.16597832	-0.42750099	-0.13250584
H	1.52831352	-0.47802180	-1.16674617
H	1.94642600	-0.81903357	0.52352675
C	-0.73140952	0.99269367	-0.22832842
H	-1.33905658	1.75609085	0.25781493
H	-0.79059333	1.14649258	-1.30852975

C	0.73139749	0.99270472	0.22832054
H	0.79057754	1.14652538	1.30851887
H	1.33903672	1.75610001	-0.25783545

[BiMe(phen)$_2$]$^{2+}$

Bi	0.017490067	-0.612997793	-0.216558258
N	1.618001471	1.155262569	0.402637157
N	-1.678789533	1.172954197	-0.529275824
N	2.462706106	-1.403136029	-0.278318386
C	-2.734086725	-2.667066771	0.448401185
H	-1.940244555	-3.405013795	0.469312423
C	-2.984637156	0.870946190	-0.285566263
C	-3.352991285	-0.467831860	0.089299379
C	3.384557131	-0.420323401	-0.134065261
C	-0.054997950	-0.854649849	2.021824652
H	0.943081680	-0.706669553	2.423621884
H	-0.420607537	-1.854486582	2.247006622
H	-0.746978270	-0.116534178	2.423996234
C	-4.000657987	1.844161122	-0.447913108
C	2.947628251	0.895064629	0.250195728
C	2.859320490	-2.622567864	-0.612301004
H	2.090520675	-3.379179785	-0.720200615
C	-4.048292655	-3.045653769	0.743494056
H	-4.267261078	-4.073994879	0.994353431
C	-5.698354558	0.263119058	0.249161593
H	-6.728671234	0.015726659	0.469656101
C	-3.630062883	3.112065244	-0.932685263
H	-4.388492279	3.872641782	-1.069904569
C	4.202846491	-2.953799463	-0.820554429
H	4.470873704	-3.965726399	-1.089878455
C	4.765642535	-0.669150659	-0.309219272
C	3.477636848	3.132595321	1.008316910
H	4.201326223	3.908691262	1.223736226
C	1.238200519	2.321702356	0.915393877
H	0.179678772	2.462351753	1.083513569
C	-5.035778064	-2.091048995	0.698182127
H	-6.065050473	-2.348903570	0.913728435
C	-5.358979165	1.512735104	-0.149005998
H	-6.113233278	2.280984019	-0.258939642
C	2.137502296	3.338427734	1.237010691
H	1.768993045	4.266273988	1.651074510
C	3.919514869	1.890532485	0.517140110
C	-4.710075526	-0.763853320	0.356392983
C	-1.369105323	2.364388455	-1.033051327
H	-0.328632947	2.542406904	-1.270459402
C	-2.316820876	3.364033997	-1.254103534
H	-2.005992812	4.314430256	-1.664359870
C	5.154990400	-1.974691512	-0.668465977
H	6.204661772	-2.196304779	-0.814950273
C	5.305602855	1.606738786	0.311300153
H	6.025556334	2.392628523	0.498941848
C	5.711733005	0.380852154	-0.097859423
H	6.762475183	0.170415501	-0.249621116
N	-2.395588333	-1.425262364	0.134353132

[BiFMe(phen)$_2$]$^{+}$

Bi	0.028122538	-0.707712657	-0.264063166
N	1.774517015	1.368479947	0.371761970
N	-1.881663739	1.356072852	-0.574020084
N	2.564346206	-1.215589765	-0.221069592
C	-2.785074922	-2.511304554	0.422741651
H	-1.958884517	-3.210508538	0.401928171

C	-3.159846171	1.034610611	-0.275727650
C	-3.471515913	-0.325586899	0.098887889
C	3.503176328	-0.249743315	-0.075763489
C	-0.041308757	-0.623073074	1.985722244
H	0.977093452	-0.573333000	2.361711056
H	-0.532180298	-1.532641667	2.322582992
H	-0.602973403	0.255462093	2.298445709
C	-4.206894607	1.986804946	-0.352363717
C	3.092945578	1.096457498	0.247942932
C	2.941845068	-2.459137726	-0.479485674
H	2.144291064	-3.185686320	-0.567917024
C	-4.081423194	-2.922764302	0.757898097
H	-4.263897167	-3.958691644	1.006921682
C	-5.829151510	0.342922994	0.367503248
H	-6.842147316	0.059689909	0.623890319
C	-3.879898750	3.284011781	-0.789165635
H	-4.658319224	4.033616369	-0.860206771
C	4.286112563	-2.826339174	-0.622155570
H	4.537493828	-3.856220576	-0.833279512
C	4.883200837	-0.536805254	-0.199325343
C	3.653360557	3.370711573	0.843214704
H	4.386460037	4.148952561	1.015649852
C	1.405891635	2.576729150	0.759549897
H	0.343014950	2.739514327	0.888534809
C	-5.092463213	-1.995089856	0.757042872
H	-6.107665804	-2.276836867	1.007846107
C	-5.542104021	1.612146511	-0.007610347
H	-6.320773685	2.362459455	-0.059906239
C	2.311954091	3.615400725	1.009757357
H	1.948994644	4.583760753	1.325136430
C	4.084042616	2.086564679	0.461865775
C	-4.810075950	-0.658911259	0.416118223
C	-1.614035325	2.574935218	-1.008886194
H	-0.583196097	2.778352291	-1.275002077
C	-2.583675369	3.578223859	-1.136103416
H	-2.303518519	4.557994732	-1.497380677
C	5.255029115	-1.863859063	-0.486452242
H	6.304298757	-2.111602483	-0.589316486
C	5.466678218	1.760025252	0.306400920
H	6.202947714	2.538659572	0.460159987
C	5.849130809	0.501296064	-0.015682390
H	6.897537145	0.255080121	-0.126605786
F	0.056351006	-2.812233145	-0.189382605
N	-2.489385741	-1.258553099	0.109463252

$[Bi_2FMe_2(phen)_4]^{3+}$

Bi	-2.278007439	-0.319664151	-0.161036143
Bi	2.278828061	-0.318784586	0.160949507
N	1.226163584	1.856705301	1.281073793
N	2.596737363	-2.695915064	-0.560964032
N	3.601297347	2.073423011	-0.131778880
N	-4.645710877	-1.132874768	-0.437491996
N	-3.603050363	2.071704000	0.131741805
N	-2.595618619	-2.696533043	0.561710802
C	0.133889218	1.760558642	2.022777976
H	-0.211672051	0.759360102	2.245358801
C	2.934708632	3.197285573	0.222358980
C	-0.102770230	4.125696258	2.196899955
H	-0.618234055	5.011075213	2.546766790
C	1.716897449	3.084524368	0.990243576
C	-0.135482089	1.760519382	-2.022997203
H	0.210711032	0.759531672	-2.245536218

N	4.646833855	-1.131454869	0.436800353
C	-0.563600367	2.869923948	2.511127771
H	-1.443031586	2.727201860	3.123324215
C	-2.936861277	3.195854048	-0.222248167
C	-1.719059768	3.083647071	-0.990272257
C	3.399118099	4.484318505	-0.143608302
C	-3.851164922	-3.206210205	0.508939694
C	-2.225629311	-0.988079913	-2.314035842
H	-2.685991589	-1.970329324	-2.380226597
H	-1.187599167	-1.032225694	-2.634049201
H	-2.775409425	-0.271309760	-2.920385259
C	1.058778083	4.265220359	1.415805718
C	5.606644330	-0.418352046	1.012260016
H	5.341766087	0.577956369	1.341185323
C	-3.401693160	4.482646136	0.143993693
C	3.852407722	-3.205293750	-0.508647138
C	-4.912380078	-2.402108447	-0.035665321
C	6.905869887	-0.901467022	1.193227311
H	7.647644943	-0.276869140	1.670844831
C	-1.611743368	-3.425791638	1.070458375
H	-0.634599575	-2.960484728	1.089920709
C	1.612760712	-3.425565283	-1.068952886
H	0.635501336	-2.960485866	-1.088077029
C	0.561404355	2.870233840	-2.511398578
H	1.440848492	2.727976533	-3.123686220
C	-1.577469156	5.544865056	-1.044798295
H	-1.057709785	6.430863455	-1.386043001
C	2.227707136	-0.985839006	2.314344840
H	2.778634491	-0.269160740	2.919743435
H	2.687371752	-1.968418545	2.380690405
H	1.189936326	-1.029004227	2.635367895
C	-4.570226727	4.562821818	0.925281343
H	-4.953174072	5.532831142	1.217249550
C	4.687726704	2.184119261	-0.882412249
H	5.182248183	1.261124181	-1.161143120
C	4.913671213	-2.400734915	0.035167493
C	-1.810182953	-4.722637220	1.553821182
H	-0.977409001	-5.276068028	1.964487895
C	-4.135220755	-4.516823122	0.959880380
C	1.811253537	-4.722542741	-1.551949123
H	0.978398901	-5.276328584	-1.961970017
C	-7.208647488	-2.169360920	-0.752579336
H	-8.208450164	-2.569757414	-0.864908092
C	7.210257192	-2.167173471	0.750825913
H	8.210212430	-2.567293766	0.862773704
C	1.574285393	5.545662791	1.045048246
H	1.054114275	6.431403745	1.386328286
C	-5.605382613	-0.420166685	-1.013687594
H	-5.340623117	0.576133401	-1.342727261
C	0.099995224	4.125763961	-2.197065276
H	0.615009698	5.011404827	-2.546931650
C	-2.693941196	5.649482075	-0.284886831
H	-3.080917945	6.620877882	-0.004247154
C	-6.904380836	-0.903691615	-1.195185632
H	-7.646113134	-0.279418376	-1.673294599
C	-6.206695453	-2.960122385	-0.157983356
C	-1.061498679	4.264676307	-1.415797278
C	6.208194929	-2.958356002	0.156963842
C	6.457893882	-4.287336862	-0.308687839
H	7.459345368	-4.687335891	-0.216356030
C	-4.689434936	2.181886922	0.882486736
H	-5.183662123	1.258659284	1.161032633

C	5.209247086	3.411484410	-1.307372082
H	6.104801950	3.436759085	-1.912493093
C	4.136563559	-4.515980286	-0.959319510
C	-5.211335199	3.408991388	1.307765044
H	-6.106835949	3.433841773	1.912983815
C	2.690853595	5.650822534	0.285347020
H	3.077516579	6.622407098	0.004925709
C	5.465548266	-5.032293294	-0.853606570
H	5.661467199	-6.035910057	-1.208207261
C	3.072646878	-5.268079690	-1.493396538
H	3.259075790	-6.271858815	-1.854346488
C	-3.071417504	-5.268492083	1.494791598
H	-3.257808866	-6.272185847	1.855997833
C	4.567705907	4.565037393	-0.924734808
H	4.950367618	5.535230167	-1.216469533
C	-6.456269096	-4.289032203	0.307914922
H	-7.457552621	-4.689349913	0.215151520
C	-5.464010636	-5.033537570	0.853602803
H	-5.659839027	-6.037107850	1.208390467
F	0.000462339	-0.946245197	0.000703971
N	-1.227766334	1.856070399	-1.281213184

SbF_5

Sb	0.00000000	0.00000000	0.00003900
F	0.00000000	0.00000000	-1.86306700
F	0.00000000	1.87284200	0.00014500
F	0.00000000	-1.87284200	0.00014500
F	1.61366600	0.00000000	0.93127700
F	-1.61366600	0.00000000	0.93127700

$[SbF_6]^-$

Sb	0.00000000	0.00000000	0.00000000
F	0.00000000	0.00000000	1.90876100
F	0.00000000	1.90876100	0.00000000
F	0.00000000	-1.90876100	0.00000000
F	0.00000000	0.00000000	-1.90876100
F	-1.90876100	0.00000000	0.00000000
F	1.90876100	0.00000000	0.00000000

$SiMe_3F$

Si	0.000001323	0.000001732	0.027155979
C	0.986101991	-1.485906664	-0.523843064
H	1.025179794	-1.544878283	-1.614699368
H	2.012702113	-1.435098997	-0.154280774
H	0.540502282	-2.412122259	-0.154204468
C	-1.779893757	-0.111048247	-0.523826581
H	-2.249163326	-1.025553610	-0.154325077
H	-2.359245561	0.737915977	-0.154125481
H	-1.850505513	-0.115328857	-1.614683398
C	0.793769551	1.596939788	-0.523860105
H	0.236430306	2.460594678	-0.154349334
H	1.818675819	1.674178118	-0.154172451
H	0.825353527	1.660236722	-1.614716667
F	0.000020590	0.000013665	1.646505757

$[SiMe_3]^+$

Si	-0.000000732	0.000002839	-0.000000728
C	0.207656294	-1.816700141	-0.000000601
H	0.795047525	-2.117041663	0.875715141
H	-0.740346118	-2.353523477	-0.000030399
H	0.795106465	-2.117040917	-0.875676299
C	1.469485400	1.088183611	0.000001032

H	2.408384976	0.535592645	0.000026941
H	1.435898102	1.747052234	-0.875713156
H	1.435870717	1.747095298	0.875681025
C	-1.677141305	0.728514006	-0.000000242
H	-1.668045780	1.817919810	-0.000015093
H	-2.230962047	0.369947703	-0.875684808
H	-2.230945923	0.369973769	0.875705700
